# Extensive rewiring of epithelial-stromal co-expression networks in breast cancer

**DOI:** 10.1186/s13059-015-0675-4

**Published:** 2015-06-19

**Authors:** Eun-Yeong Oh, Stephen M Christensen, Sindhu Ghanta, Jong Cheol Jeong, Octavian Bucur, Benjamin Glass, Laleh Montaser-Kouhsari, Nicholas W Knoblauch, Nicholas Bertos, Sadiq MI Saleh, Benjamin Haibe-Kains, Morag Park, Andrew H Beck

**Affiliations:** Cancer Research Institute, Beth Israel Deaconess Cancer Center, Boston, MA 02215 USA; Department of Pathology, Beth Israel Deaconess Medical Center, Boston, MA 02215 USA; Harvard Medical School, Boston, MA 02215 USA; Goodman Cancer Research Centre, McGill University, Montreal, QC Canada; Princess Margaret Cancer Centre, University Health Network, Toronto, ON M5G 1L7 Canada; Department of Medical Biophysics, University of Toronto, Toronto, Ontario M5G 1L7 Canada

## Abstract

**Background:**

Epithelial-stromal crosstalk plays a critical role in invasive breast cancer pathogenesis; however, little is known on a systems level about how epithelial-stromal interactions evolve during carcinogenesis.

**Results:**

We develop a framework for building genome-wide epithelial-stromal co-expression networks composed of pairwise co-expression relationships between mRNA levels of genes expressed in the epithelium and stroma across a population of patients. We apply this method to laser capture micro-dissection expression profiling datasets in the setting of breast carcinogenesis. Our analysis shows that epithelial-stromal co-expression networks undergo extensive rewiring during carcinogenesis, with the emergence of distinct network hubs in normal breast, and estrogen receptor-positive and estrogen receptor-negative invasive breast cancer, and the emergence of distinct patterns of functional network enrichment. In contrast to normal breast, the strongest epithelial-stromal co-expression relationships in invasive breast cancer mostly represent self-loops, in which the same gene is co-expressed in epithelial and stromal regions. We validate this observation using an independent laser capture micro-dissection dataset and confirm that self-loop interactions are significantly increased in cancer by performing computational image analysis of epithelial and stromal protein expression using images from the Human Protein Atlas.

**Conclusions:**

Epithelial-stromal co-expression network analysis represents a new approach for systems-level analyses of spatially localized transcriptomic data. The analysis provides new biological insights into the rewiring of epithelial-stromal co-expression networks and the emergence of epithelial-stromal co-expression self-loops in breast cancer. The approach may facilitate the development of new diagnostics and therapeutics targeting epithelial-stromal interactions in cancer.

**Electronic supplementary material:**

The online version of this article (doi:10.1186/s13059-015-0675-4) contains supplementary material, which is available to authorized users.

## Background

Carcinomas are composed of malignant epithelial cells and a complex milieu of stromal cells in the tumor microenvironment (including endothelial cells, fibroblasts, myofibroblasts, smooth muscle cells, adipocytes, and inflammatory cells) [1, 2]. Stromal expression patterns and morphologic phenotypes are correlated with disease outcome [3–12], and the tumor microenvironment plays essential roles in supporting the initiation, progression, and metastatic spread, as well as drug resistance, in cancer [1, 2, 13–25]. Communication between the epithelium and stroma is mediated through physical interactions between epithelial and stromal cells, through physical interactions of epithelial and stromal cells with the intermediating extracellular matrix, and through the expression of signaling molecules that are relayed between the epithelium and stroma in a process known as epithelial-stromal crosstalk [15, 24]. Well-characterized classes of molecules involved in epithelial-stromal crosstalk include cytokines, adipokines, proteases, angiogenic factors, and growth factors [13, 16].

Despite an increasing appreciation of the critical role of epithelial-stromal crosstalk in carcinogenesis, little is known on a systems level about the evolution of epithelial-stromal crosstalk network connectivity during the process of carcinogenesis. The increasing availability of tissue region- and cell type-specific tissue sampling methods [26–30] and the recent development of methods for spatially resolved transcriptomics [31–34] and highly multiplexed *in situ* assessment of protein expression [35–37] have created new opportunities for comprehensively characterizing tissue region- and cell type-specific molecular features of the cancer epithelium and stroma. Several cell type- or tissue region type-specific transcriptional profiling studies have been completed in the setting of breast carcinogenesis [38–43]. In each of these analyses, the investigators isolated RNA from stromal and epithelial cell populations [38] or stromal and epithelial tissue regions [39–43] at various stages of breast carcinogenesis, and subsequently performed statistical analyses to identify genes and biological pathways within each tissue compartment associated with breast cancer progression and/or clinical outcome. A limitation of this differential expression analytic approach is that it does not allow direct evaluation of epithelial-stromal co-expression relationships, e.g., increased expression of gene *X* in the stroma is associated with decreased expression of gene *Y* in the epithelium in breast cancer. A further limitation of differential expression-based analytic approaches is they do not permit a systems-based analysis of global patterns of network connectivity and rewiring in disease progression. Network models offer important new opportunities for identifying prognostic and predictive network features driving disease [44–46]. For example, a recent network-based analysis of factors associated with late-onset Alzheimer’s disease identified an overlap of only 6 % between standard differential gene expression-based signatures and network connectivity-based signatures of disease progression [47]. We expect that systems-based approaches will be particularly well-suited to the study of epithelial-stromal interactions in carcinogenesis, because the process of epithelial-stromal crosstalk possesses the core characteristics that fuel the emergence of complex adaptive systems, defined as systems comprising interdependent, diverse, connected entities, that adapt to local and global environmental forces [48].

Thus, we developed a computational approach for evaluating genome-wide epithelial-stromal co-expression networks from high-dimensional molecular measurements obtained from paired epithelial and stromal samples. While co-expression networks have been previously used to identify prognostic pathways [49] and to infer cellular phenotypes from bulk expression profiling samples in cancer [50], no prior studies have modeled epithelial-stromal interactions genome-wide using an epithelial-stromal co-expression network-based approach. Here, we apply our method to laser capture microdissection (LCM)-derived gene expression data obtained from histologically normal breast, estrogen receptor (ER)-positive invasive breast cancer (IBC), and ER-negative IBC. To construct epithelial-stromal co-expression networks, we computed all pairwise co-expression interactions between epithelial and stromal mRNA levels, generating an epithelial-stromal co-expression network, where each node in the network is a gene and each edge represents an epithelial-stromal co-expression relationship. We then applied network analytics to identify network hubs, to determine network functional enrichment, and to assess global differences in network connectivity in normal breast, ER-positive IBC, and ER-negative IBC. Lastly, we used an independent LCM dataset and a large collection of breast cancer immunohistochemistry images provided by the Human Protein Atlas [51, 52] to validate predictions made by the epithelial-stromal co-expression network analyses.

## Results

### A systems approach to the analysis of epithelial-stromal co-expression in breast cancer

**Fig. 1:**
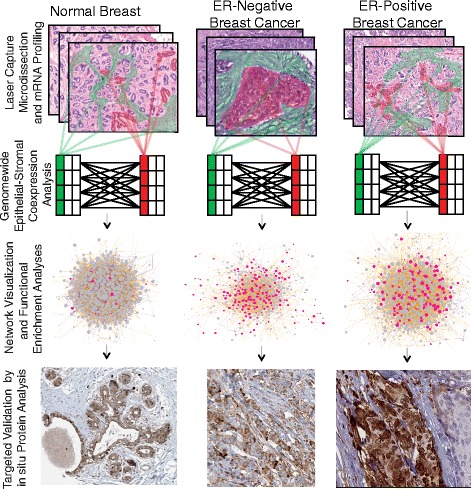
Overview of the epithelial-stromal co-expression network approach. Using laser capture microdissection gene expression profiling data from paired epithelial and stromal samples from cases of normal breast, ER-negative -IBC, and ER-positive -IBC (top panel), we constructed genome-wide epithelial-stromal co-expression networks (second panel), performed network visualization and functional enrichment analyses (third panel), and validated network predictions by several approaches, including measurement of protein expression by computational image analysis in the epithelium and stroma (bottom panel). In the top and second panels, red indicates epithelial tissue and data (respectively) and green indicates stromal tissue and data (respectively)

Our approach has four basic stages (Fig. 1):

i. Data preparation: Obtain tissue region-specific transcriptional profiling data from epithelial and stromal tissue compartments from samples at various stages of carcinogenesis.
ii. Co-expression analysis: Perform comprehensive computation of co-expression relationships between epithelial and stromal mRNA levels from patient-matched epithelial and stromal samples.
iii. Network analysis: Perform network analyses to identify network hubs, differential network features, and differential functional enrichment between normal breast, ER-positive IBC, and ER-negative IBC.
iv. Validation: Validate predicted epithelial-stromal co-expression relationships by additional approaches, including independent LCM data and *in situ* analyses of protein expression by immunohistochemistry.

### Assembly of an LCM dataset of paired epithelial and stromal samples in normal breast, ER-negative invasive breast cancer, and ER-positive invasive breast cancer

We searched the NCBI Gene Expression Omnibus (GEO) [53] to identify breast cancer LCM datasets. The search keywords used to identify the studies were “breast cancer,” “epithelium and stroma,” and “laser capture microdissection.” We identified five datasets containing at least five epithelial-stromal pairs of LCM samples captured from histologically normal breast and/or IBC [GEO:GSE4823, GEO:GSE5847, GEO:GSE10797, GEO:GSE14548, and GEO:GSE35019]. These datasets come from previously published studies [39–41, 43, 54]. Together, these datasets contain 82 epithelial-stromal pairs from IBC and 41 pairs from histologically normal breast tissue. The five LCM datasets were generated by one of four expression profiling platforms, namely Affymetrix U133A2.0, Affymetrix U133X3P, Agilent Whole Human Genome Oligo Microarray G4112A, and Illumina Whole Genome DASL. To identify common gene symbols measured across the four platforms, we used the Array Information Library Universe Navigator (AILUN) platform comparison tool [55]. We restricted our analyses to gene symbols measured across all four platforms, resulting in a total of 11,700 genes. For gene symbols with multiple probe sets, we selected the probe with the greatest variance within each dataset. We centered and scaled gene expression values in each dataset within each tissue compartment by subtracting out the population mean expression and dividing by the population standard deviation, with the population defined as samples with the same pathological diagnosis (normal or IBC) from the same dataset. We stratified the IBC cases into ER-positive (n = 54) and ER-negative (n = 28) groups based on the estrogen receptor 1 (*ESR1*) gene expression levels in the epithelium, using univariate Gaussian mixture model-based clustering via the *mclust* package in R.

### Assessment of batch effect prior to data integration

To assess for the presence of batch effects across datasets, we used the procedure recommended by [56]. First, using the merged dataset we visually inspected the epithelial expression of *ESR1* in breast cancer (Additional file 1); the results of unsupervised hierarchical clustering of samples using all genes (Additional file 2); and a scatterplot of samples along the first two principal components of the gene expression data (Additional file 3). In each of these analyses, we did not see a strong association of dataset with sample cluster, providing no strong evidence of overall batch effects. However, these analyses represent exploratory methods to visualize batch effect and do not directly address the impact of batch on epithelial-stromal co-expression relationships. To directly and statistically assess the influence of dataset on epithelial-stromal co-expression relationships in normal breast, ER-positive IBC, and ER-negative IBC, we again followed an analysis strategy suggested by [56]. We selected the two largest datasets from each diagnostic category and performed the epithelial-stromal co-expression analyses separately within each dataset. For each of normal breast, ER-positive IBC, and ER-negative IBC, we then assessed the overall agreement in direction of associations for co-expression relationships identified as significant using a raw *p*-value threshold of 0.001. After computing agreement with the true dataset labels, we then shuffled the “dataset” label and repeated this procedure for 100 iterations and assessed whether the agreement tended to be significantly higher with the dataset labels shuffled as compared with the agreement obtained with the true dataset labels (Additional file 4). This analysis demonstrated significant evidence of batch effect across the two normal breast datasets, but no significant batch effect across the ER-positive and ER-negative IBC datasets (Additional file 4).

The significant batch effect in the normal datasets could be due to a variety of different pre-analytic factors, which may have an especially large impact on studies of normal breast, including the significant heterogeneity in the proportions of normal cell types (e.g., epithelial, fat, stroma, immune) encountered in the normal breast across a population of patients, and the relative lack of standardized methods for sampling and handling normal breast tissue. This latter point is in contrast to IBC specimens, which are much more frequently profiled using transcriptional profiling approaches. Given the extent of the batch effect in the normal samples, we chose to exclude the GSE14548 normal samples (n = 14) from the analysis and to focus our epithelial-stromal co-expression analysis on the GSE4823 dataset, which was the largest normal breast dataset (n = 22) and the only dataset to include technical replicates (as part of a dye-swap experimental setup). We confirmed strong intra-replicate correlation for all normal epithelial and stromal samples from GSE4823 (Additional file 5), which further supported the quality of this dataset for constructing a normal breast epithelial-stromal co-expression network.

### Genome-wide computation of epithelial-stromal co-expression relationships in normal breast, ER-negative invasive breast cancer, and ER-positive invasive breast cancer

For each gene within each diagnostic class (normal breast, ER-negative IBC, and ER-positive IBC), we used simple linear regression to build univariate models linking gene expression in the stroma with gene expression in the epithelium. We exhaustively computed all pairwise associations of epithelial and stromal gene expression in normal breast, ER-negative IBC, and ER-positive IBC. In total, we computed 11,700 × 11,700 = 136,890,000 pairwise associations between epithelial and stromal gene expression in each of normal breast, ER-negative IBC, and ER-positive IBC, resulting in the evaluation of approximately 411 million epithelial-stromal co-expression associations. We performed the co-expression analysis and computed false discovery rates (FDRs) using the matrixEQTL package [57]. To enable quantitative comparisons of epithelial-stromal co-expression networks in normal breast, ER-negative IBC, and ER-positive IBC, we standardized the number of edges in each network by constructing networks from the most significant 10,000 interactions in each of the three diagnostic categories. Network visualization and analyses were performed using the *igraph* [58], *RedeR* [59], and *SANTA* [60] software packages in R [61].

### Epithelial-stromal co-expression network connectivity and emergence of self-loops

**Fig. 2:**
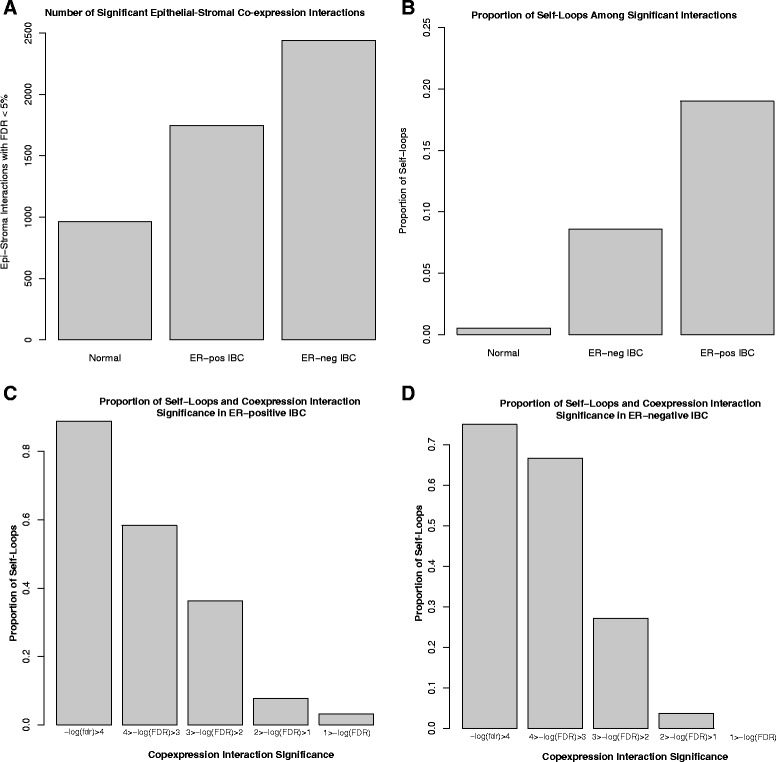
Invasive breast cancer is associated with increased self-loops. **a** Number of significant epithelial-stromal co-expression interactions. The *y-axis* indicates the number of significant epithelial-stromal co-expression relationships (FDR < 5 %) for epithelial-stromal co-expression networks in normal breast, ER-negative IBC, and ER-positive IBC. **b** Proportion of self-loops among significant epithelial-stromal co-expression interactions. The *y-axis* indicates the proportion of significant relationships (FDR < 5 %) that are self-loop relationships in normal breast, ER-negative IBC, and ER-positive IBC. There is a significantly higher proportion of self-loop relationships among statistically significant interactions in IBC as compared with normal breast (*p* < 2.2e-16). **c**, **d** Proportion of self-loops and co-expression interaction significance in ER-positive IBC (c) and ER-negative IBC (d). The *y-axis* indicates the proportion of self-loop relationships among epithelial-stromal crosstalk interactions at progressive levels of statistical significance. The windows of statistical significance are indicated on the *x-axis*. There is a significant trend for a higher proportion of self-loops among the most significant co-expression relationships in ER-positive IBC (c) and ER-negative IBC (d) (both *p* < 2.2e-16). *ER* estrogen receptor; *IBC* invasive breast cancer; *FDR* false discovery rate

We compared the overall number of network edges with FDR < 5 % in the normal breast, ER-positive IBC, and ER-negative IBC epithelial-stromal co-expression networks. Overall, we identified the highest number of significant connections in ER-negative IBC, followed by ER-positive IBC, and then normal breast (Fig. 2a). A much larger proportion of network edges represented epithelial-stromal “self-loops” in breast cancer, as compared with normal breast (Fig. 2b), and the most significant network edges in breast cancer represented epithelial-stromal self-loops in both ER-positive (Fig. 2c, Table 1) and ER-negative (Fig. 2d, Table 1) IBC networks.

**Table 1:** Top-ranked epithelial-stromal co-expression relationships in normal breast, ER-negative IBC, and ER-positive IBC

Normal breast				
Epithelium	Stroma	T-Stat	*p*-value	FDR
*IPCEF1*	*SPINK1*	29.29	6.70e-18	9.17e-10
*HSPA12A*	*PNMA2*	21.15	3.71e-15	1.74e-07
*ALDOB*	*PNMA2*	21.11	3.85e-15	1.74e-07
*SULT1E1*	*PNMA2*	20.80	5.09e-15	1.74e-07
*DPT*	*SPINK1*	19.98	1.10e-14	2.90e-07
*SFTPB*	*PNMA2*	19.83	1.27e-14	2.90e-07
*ADAM28*	*PLCL1*	18.80	3.52e-14	6.88e-07
*SCN11A*	*SYNPO2L*	18.10	7.18e-14	1.23e-06
*HSPA12A*	*CFTR*	17.45	1.43e-13	2.18e-06
*IPCEF1*	*SLC4A10*	−16.73	3.17e-13	4.34e-06
**ER-positive IBC**				
Epithelium	Stroma	T-Stat	*p*-value	FDR
*CEACAM5*	*CEACAM5*	18.05	4.84e-24	6.65e-16
*S100A7*	*S100A7*	14.78	2.93e-20	2.01e-12
*FAM5C*	*FAM5C*	14.24	1.42e-19	6.49e-12
*BEX1*	*BEX1*	12.70	1.46e-17	5.01e-10
*IFIH1*	*IFIH1*	11.00	3.55e-15	9.74e-08
*AGT*	*AGT*	10.74	8.34e-15	1.91e-07
*BAMBI*	*BAMBI*	10.62	1.26e-14	2.48e-07
*PCDH8*	*PCDH8*	10.29	3.80e-14	6.53e-07
*S100A8*	*S100A8*	10.06	8.42e-14	1.28e-06
*ATHL1*	*ATHL1*	9.95	1.25e-13	1.72e-06
**ER-negative IBC**				
Epithelium	Stroma	T-Stat	*p*-value	FDR
*ORM1*	*ORM1*	19.29	6.32e-17	8.68e-09
*PCP4*	*PCP4*	13.95	1.40e-13	9.62e-06
*MMP10*	*MMP10*	13.65	2.29e-13	1.05e-05
*DSC3*	*DSC3*	13.39	3.53e-13	1.21e-05
*NPY5R*	*CPB1*	12.46	1.82e-12	4.99e-05
*IMPA2*	*IMPA2*	12.10	3.46e-12	7.91e-05
*ASPM*	*SRPK1*	12.02	4.07e-12	7.99e-05
*LCN2*	*LCN2*	11.83	5.72e-12	9.81e-05
*KRT16*	*KRT16*	11.37	1.36e-11	2.08e-04
*SH3GL2*	*SH3GL2*	11.31	1.55e-11	2.13e-04

*ER* estrogen receptor, *FDR* false discovery rate, *IBC* invasive breast cancer

### Epithelial-stromal co-expression network hubs in normal breast and breast cancer

To identify genes with the most connections in the epithelial-stromal co-expression networks, we computed the network degree for each gene in each network. The ten most highly connected genes in each network are presented in Table 2 and the network degrees of all genes in the networks are provided in Additional file 6. In the normal breast, ER-positive IBC, and ER-negative IBC networks, the most highly connected genes contributed to the co-expression networks primarily through their stromal expression (Table 2) (*p* < 0.03 in each network).

**Table 2:** Top-ranked genes by epithelial-stromal co-expression network degree in normal breast, ER-negative IBC, and ER-positive IBC

Normal breast				
	Gene	Stromal degree	Epithelial degree	Self-loop
1	*GABRA6*	56	11	No
2	*FGF22*	63	0	No
3	*POU3F1*	54	6	No
4	*FPR3*	58	0	No
5	*RPE65*	20	32	No
6	*ASPM*	51	0	No
7	*ARL3*	50	0	No
8	*HHIPL2*	45	0	No
9	*HTR2A*	12	32	No
10	*ABI3BP*	43	0	No
**ER-positive IBC**				
	Gene	Stromal degree	Epithelial degree	Self-loop
1	*BDNF*	61	2	No
2	*IFIH1*	37	19	Yes
3	*FUT5*	35	18	No
4	*KIF20A*	29	23	Yes
5	*UBE2C*	26	26	Yes
6	*FOXM1*	33	16	Yes
7	*CCNB2*	34	12	No
8	*KIF4A*	28	11	Yes
9	*DSC3*	33	5	Yes
10	*MGAM*	1	35	Yes
**ER-negative IBC**				
	Gene	Stromal degree	Epithelial degree	Self-loop
1	*NTS*	79	0	No
2	*MYRF (C11orf9)*	63	0	No
3	*SRPK1*	61	0	No
4	*DENND5B*	46	6	No
5	*BUB1*	0	48	No
6	*EZH2*	45	2	Yes
7	*DAP3*	38	8	Yes
8	*GRM1*	45	0	No
9	*KIF20A*	6	37	No
10	*DLGAP5*	11	32	Yes

The columns indicate each gene’s connections due to stromal expression (stromal degree), epithelial expression (epithelial degree), and whether it is in a self-loop in the table’s tissue (normal, ER-positive IBC, or ER-negative IBC). *ER* estrogen receptor, *IBC* invasive breast cancer

In normal breast, the set of most highly connected genes included genes involved in cell surface receptor linked signal transduction, including *HTR2A*, *FGF22*, *FPR3*, *GABRA6*, and *RPE65*; several of these are involved in neuroactive ligand receptor interaction (*HTR2A*, *FPR3*, *GABRA6*).

In the ER-positive IBC epithelial-stromal co-expression network, the most connected gene was brain-derived neurotrophic factor (*BDNF*) (Table 2), which is a secreted growth factor most highly expressed by smooth muscle in normal tissues [62]. *BDNF*’s contributions to the network were predominantly stromal (61/63, 97 % of interactions). *BDNF* expression has been shown to significantly impact breast cancer cell survival [63–65], to be a pro-oncogenic target of microRNAs in breast cancer [66, 67], and to be associated with decreased patient survival in breast cancer [68]. The second most connected gene in ER-positive IBC was the *IFIH1* transcript, encoding the melanoma differentiation-associated protein 5 (MDA5), and the next most connected gene was *FUT5*, encoding the alpha-(1,3)-fucosyltransferase enzyme. The next five most connected genes in ER-positive IBC (*KIF20A*, *UBE2C*, *FOXM1*, *CCNB2*, *KIF4A*) are all associated with cell division, cell cycle, and proliferation (Table 2).

In ER-negative IBC, neurotensin (*NTS*) was the most connected gene in the epithelial-stromal co-expression network and contributed to the network primarily through its stromal expression. The most-connected gene with epithelial expression was the mitotic checkpoint serine/threonine-protein kinase *BUB1* (Table 2). Stromal expression of the glutamate receptor, metabotropic 1 (*GRM1*) contributed to 45 epithelial-stromal co-expression interactions in ER-negative IBC, making it one of the top-ranked genes in this network (Table 2). *GRM1* has recently been identified as a therapeutic target in ER-negative breast cancer [69]. Our analysis further supports the importance of *GRM1*-mediated signaling in ER-negative breast cancer. The most connected gene involved in a self-loop in ER-negative IBC was the histone-lysine N-methyltransferase *EZH2*, whose co-expression interactions were primarily due to its stromal expression (Table 2). *EZH2* overexpression has been previously associated with aggressive ER-negative IBC [70, 71].

### Visualization and functional enrichment analyses of epithelial-stromal co-expression networks

**Fig. 3:**
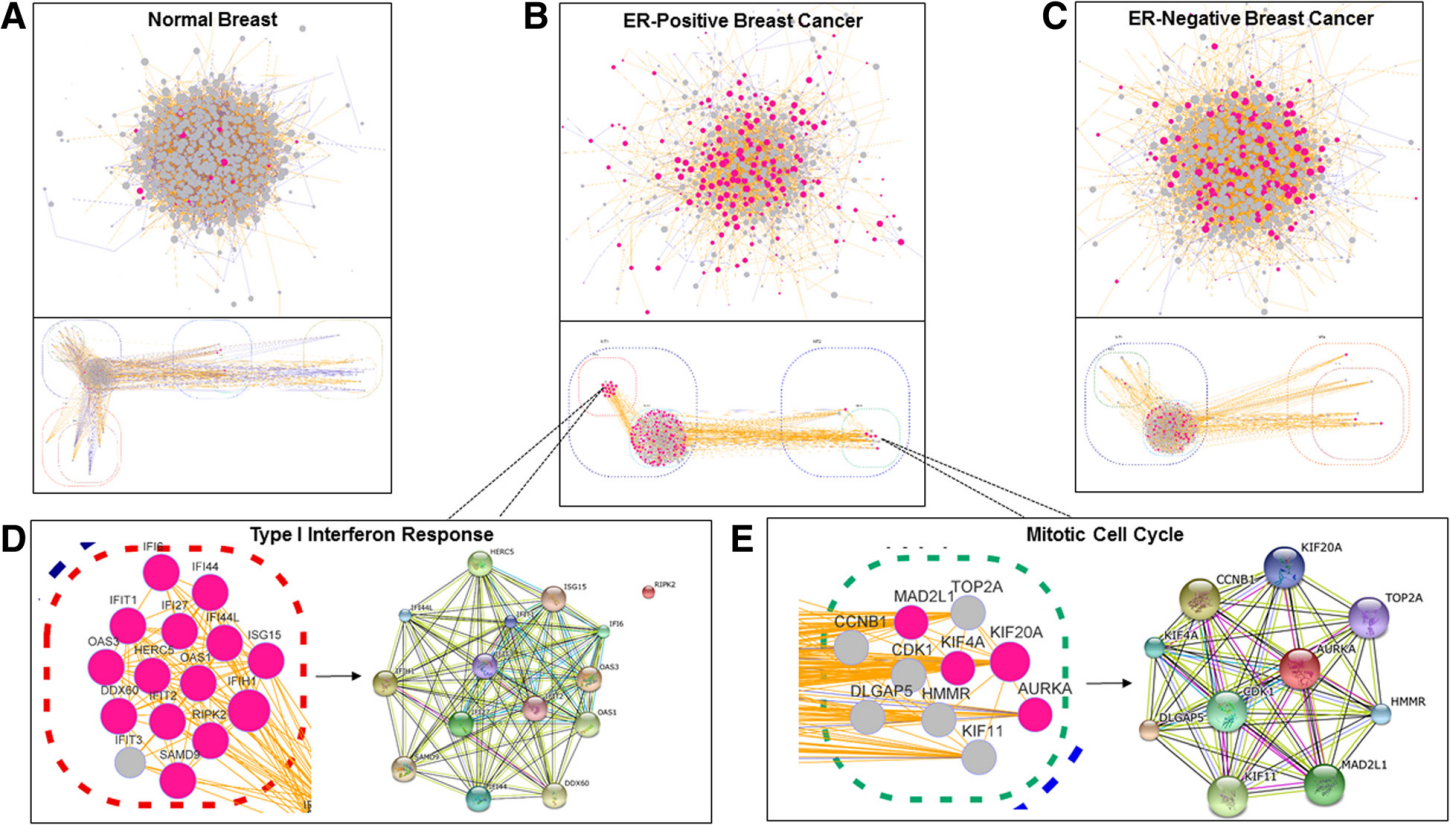
Network visualization reveals network modularity and increased self-loops in epithelial-stromal co-expression networks in invasive breast cancer. The epithelial-stromal co-expression networks are visualized for **a** normal, **b** ER-positive, and **c** ER-negative tissue. *Edge weight* indicates the statistical significance of the epithelial-stromal co-expression relationship, *edge color* indicates the direction of the association (positive is *yellow*; negative is *gray*), *node size* indicates the node’s degree, and *node color* indicates whether the node was involved in an epithelial-stromal self-loop (*pink*) or not (*gray*). The network layout was determined by a force-directed algorithm applied to the network and then by performing hierarchical clustering on the network adjacency matrix and projecting the clustering results onto the network. Panels **d** and **e** show the results of unsupervised hierarchical clustering performed on the ER-positive network adjacency matrix, which uncovered a network cluster highly enriched for proteins involved in the type 1 interferon response (d) and the mitotic cell cycle (e)

To visualize the epithelial-stromal co-expression networks, we used the *RedeR* [59] package in R. For visualization, we limited the analysis to genes with a network degree greater than five (Fig. 3). The high-level network visualizations suggest several broad differences between the normal breast and IBC networks. First, the proportion of nodes involved in epithelial-stromal self-loops (colored pink in Fig. 3) was dramatically increased in ER-positive IBC (31 % of nodes in the ER-positive IBC network figure) and ER-negative IBC (22 % of nodes in the ER-negative IBC network figure) as compared with the normal breast (2 % of nodes in the normal breast network figure) (both *p* < 2.2e-16). Second, the proportion of positive edges (colored yellow in Fig. 3) was increased in the ER-positive IBC (85 %) and ER-negative IBC (88 %) networks compared with the normal network (65 %) (both *p* < 2.2e-16). Additionally, analysis of the nested sub-networks in ER-positive breast cancer showed a very high-level of functional enrichment for several critical biological processes (e.g., type 1 interferon response and mitotic cell cycle) in network clusters, demonstrating that unsupervised hierarchical clustering alone based on the edge-weighted epithelial-stromal co-expression network adjacency matrix is able to uncover sets of genes that are highly enriched for protein-protein interactions and that contribute to important biological processes in breast cancer (Fig. 3d, e).

To systematically test for network functional enrichment and to directly compare network functional enrichment across the three epithelial-stromal co-expression networks, we used the Spatial Analysis of Network Associations (SANTA) method, which statistically tests the association between a query geneset and a network, enabling the functional annotation of networks [60]. To perform the functional enrichment analyses, we used four collections of genesets: Gene Ontology (GO) Biological processes (n = 825) [72]; Kyoto Encyclopedia of Genes and Genome (KEGG) pathways (n = 186) [73], a compendium of breast cancer prognostic signatures (n = 125) [74], and a collection of cell type-specific signatures that we compiled for this analysis (n = 42), for a total of 1178 signatures evaluated (Additional files 7 and 8).

**Fig. 4:**
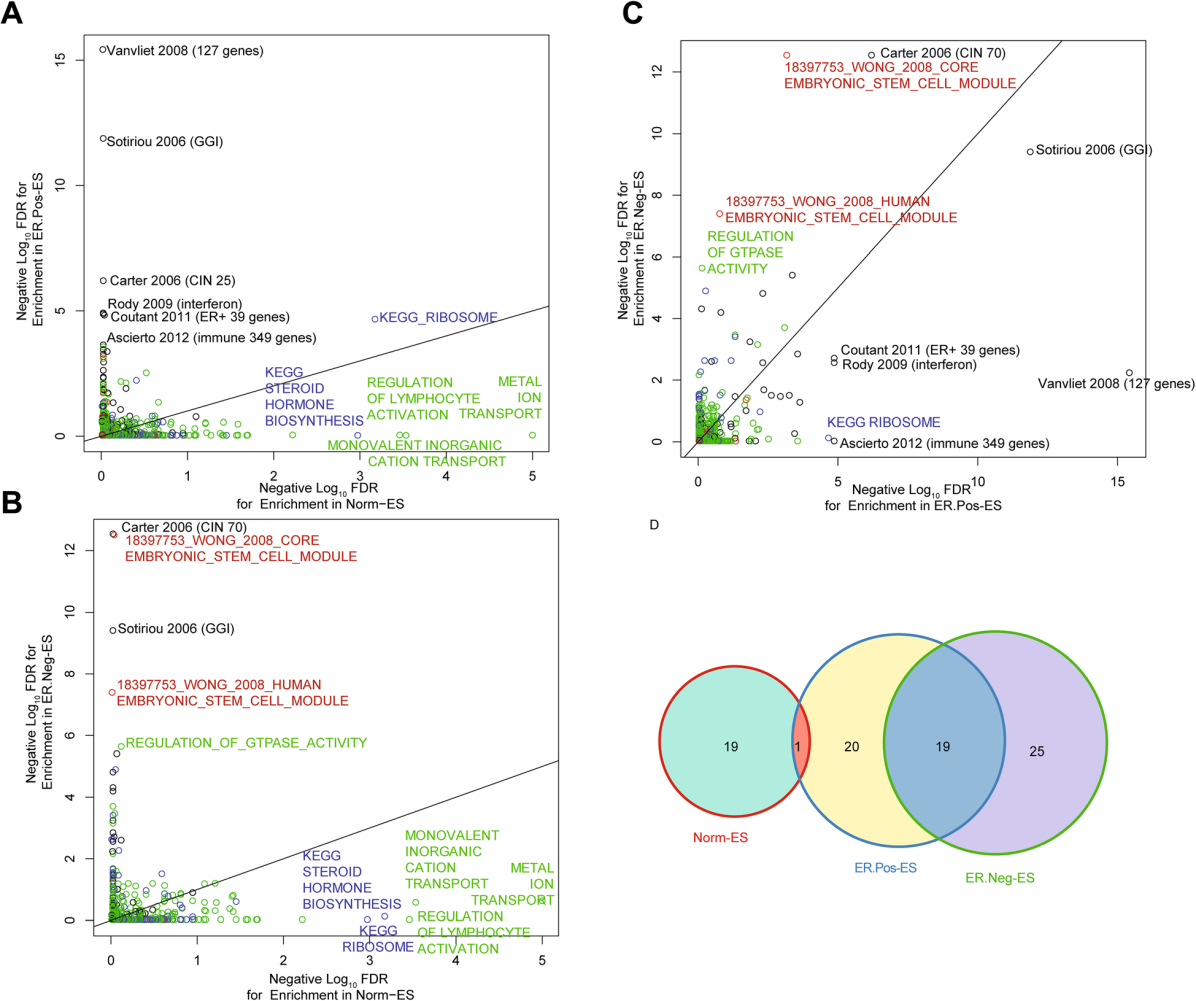
Comparative functional network enrichment analysis on the normal breast, ER-positive IBC, and ER-negative IBC epithelial-stromal co-expression networks. **a**–**c** Scatterplots where each *point* is a functional geneset plotted according to its statistical significance in two of the epithelial-stromal co-expression networks. The terms are colored according to the category of geneset, with *black* indicating a breast cancer prognostic geneset; *blue* indicating a KEGG biological pathway; *green* indicating a GO biological process; and *red* indicating a cell type-specific signature. A subset of top-ranking genesets is labeled in the plots. In a, each geneset is plotted according to its statistical significance in the ER-positive IBC network on the *y-axis* and the normal network on the *x-axis*. In b, each geneset is plotted according to its statistical significance in the ER-negative IBC network on the *y-axis* and the normal network on the *x-axis*. In c, each geneset is plotted according to its statistical significance in the ER-negative IBC network on the *y-axis* and the ER-positive IBC network on the *x-axis*. **d** Venn diagram depicting genesets identified as significantly enriched (FDR < 5 %) in at least one of the three networks. *ER estrogen receptor; ES epithelial-stromal; GO Gene Ontology; IBC invasive breast cancer; KEGG Kyoto Encyclopedia of Genes and Genomes; Neg negative; Norm normal; Pos positive*

Overall, the functional enrichment significance scores for ER-negative and ER-positive IBC networks showed moderate correlation with each other (Spearman Rho = 0.23), but little correlation with the epithelial-stromal co-expression network functional enrichment scores in normal breast (Spearman Rho = −0.06 and −0.08 with ER-positive IBC and ER-negative IBC, respectively), supporting significant functional network rewiring in breast cancer (Fig. 4). Overall, we identified a total of 20, 40, and 44 significant genesets in the normal, ER-positive IBC, and ER-negative IBC epithelial-stromal co-expression networks, respectively, at an FDR of 5 %. We identified a significant positive enrichment for genesets identified in both ER-positive IBC and ER-negative IBC, with 19 genesets identified in both ER-positive IBC and ER-negative IBC among the 65 genesets identified as significant in either network (odds ratio (OR) = 40.0, *p* < 2.2e-16). There was no significant overlap in the pathways identified as significant in the ER-positive or ER-negative IBC epithelial-stromal networks and those identified as significant in the normal network (both *p* > 0.50) (Fig. 4).

### Normal breast functional geneset enrichment

The most functionally enriched terms in the normal breast tended to be KEGG terms and GO biological processes (Fig. 4, Additional file 7), including the biological processes *metal ion transport*, *regulation of lymphocyte activation*, *alcohol metabolic process*, *lipid catabolic process,* and *neuropeptide signaling pathway*; and the KEGG terms: *ribosome* and *steroid hormone biosynthesis*. With the exception of the KEGG term *ribosome* (which was significantly enriched in ER-positive IBC), these genesets were not enriched in breast cancer.

### ER-positive breast cancer functional geneset enrichment

Breast cancer prognostic signatures (which represented 11% of the total genesets analyzed) were highly enriched among the top pathways in ER-positive IBC, representing nine of the top ten pathways in ER-positive IBC. These prognostic pathways included inflammation-related signatures (Vanvliet 2008 (127 genes); Ascierto 2012 (immune 349 genes); Rody 2009 (interferon); Rody 2009 (199 genes)), a signature associated with histologic grade and tumor proliferation in ER-positive breast cancer (Sotiriou 2006 (GGI)), a signature based on chromosomal instability (Carter 2006 (CIN 70)), a signature associated with TP53 mutation status in ER-positive breast cancer (Coutant 2011 (ER-positive 39 genes)), and a signature defined based on an association with prognosis in ER-positive breast cancer (ER-positive predictor) [74]. The top-ranking GO biological process was *unfolded protein response* and the top-ranking KEGG term was *ribosome*. These data show that a diverse set of prognostic genesets, including both immune-associated genes and proliferation associated genesets, are strongly enriched in the ER-positive IBC epithelial-stromal co-expression network.

### ER-negative breast cancer functional geneset enrichment

In ER-negative IBC, six of the top ten enriched signatures were breast cancer prognostic signatures, including chromosomal instability signatures (Carter 2006 (CIN 70); Carter 2006 (CIN 25)), a signature associated with prognosis in ER-positive breast cancer (Teschendorff 2006 (t52)), a signature associated with histologic grade in ER-positive breast cancer (Sotiriou 2006 (GGI)), a PTEN-associated signature (Saal 2007 (PTEN pathway)), and genes associated with TP53 mutation status in ER-negative breast cancer (Coutant 2011 (ER- 30 genes)). The top-ranking GO biological process was *regulation of GTPase activity* and the top-ranking KEGG pathway was *Huntington Disease*. In addition, we identified strong functional enrichment for two embryonic stem cell modules [75], supporting a link between an embryonic stem cell expression signature and epithelial-stromal co-expression in ER-negative IBC.

### Comparative functional network enrichment analysis of epithelial-stromal co-expression networks and epithelial-epithelial co-expression networks

**Fig. 5:**
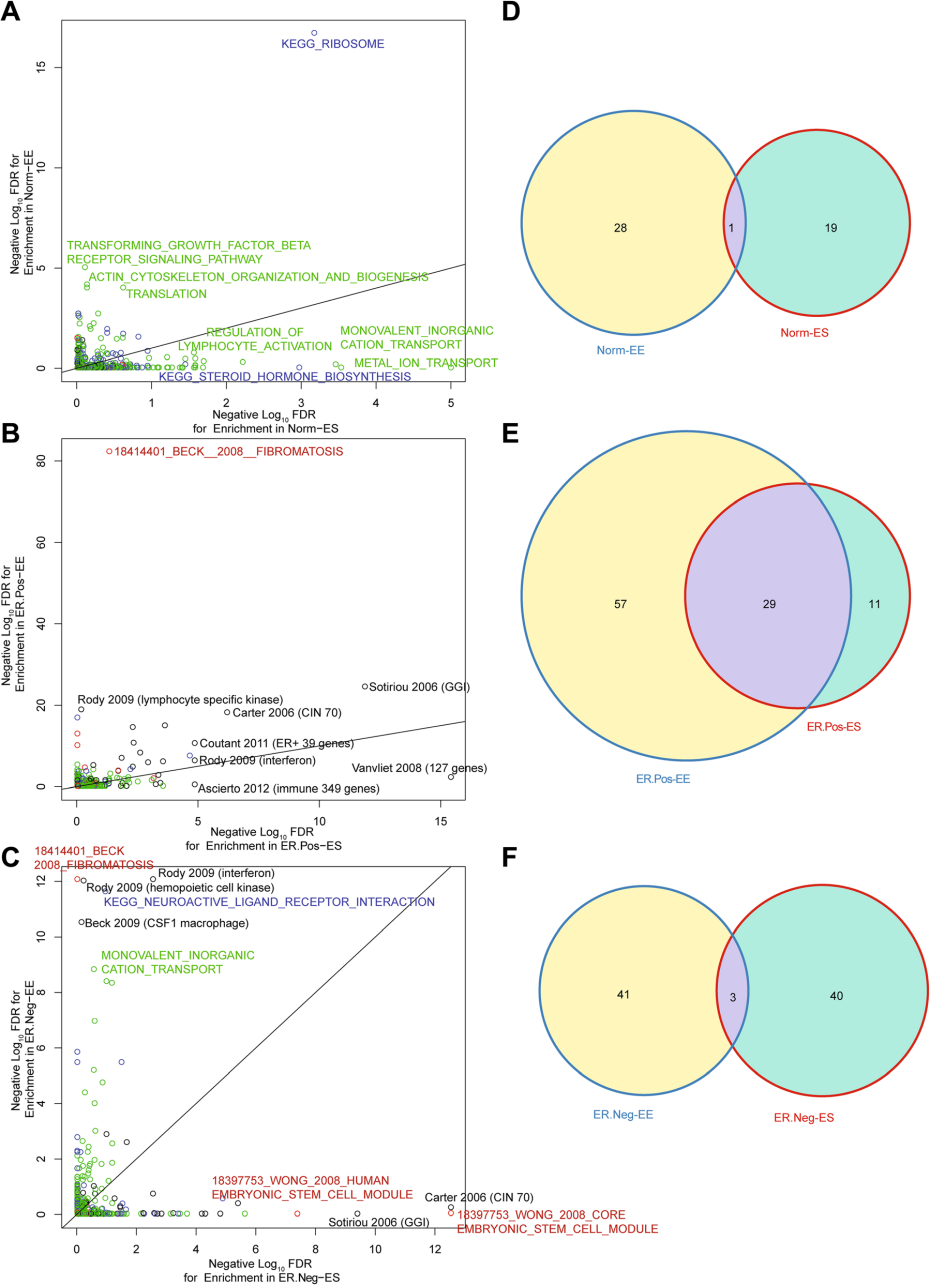
Comparative functional network enrichment analysis of the epithelial-stromal versus epithelial-epithelial co-expression networks in normal breast, ER-positiveIBC, and ER-negativeIBC. **a**–**c** Scatterplots in which each point is a functional geneset plotted according to its statistical significance in the epithelial-epithelial co-expression network (*y-axis*) and the epithelial-stromal co-expression network (*x-axis*) in normal breast, ER-positive IBC, and ER-negative IBC. Terms are colored as in Fig. 4. A subset of top-ranking genesets is labeled in the plots. **d**–**f** Venn diagrams of genesets identified as significantly enriched (FDR < 5 %) in epithelial-epithelial and/or epithelial-stromal co-expression networks in normal breast, ER-positive IBC, and ER-negative IBC, respectively. *EE epithelial-epithelial; ER estrogen receptor; ES epithelial-stromal; IBC invasive breast cancer; Neg negative; Norm normal; Pos positive*

To directly compare network functional enrichment between epithelial-epithelial co-expression networks and epithelial-stromal co-expression networks, we repeated the SANTA and functional enrichment analyses on epithelial-epithelial co-expression networks in normal breast, ER-positive IBC, and ER-negative IBC (Fig. 5). The overall pattern of functional enrichment scores in the epithelial-epithelial network was weakly correlated with the patterns observed in the epithelial-stromal co-expression networks for correlation between the epithelial-epithelial and epithelial-stromal functional enrichment scores in normal breast (Spearman Rho = 0.09), ER-positive IBC (Spearman Rho = 0.22), and ER-negative IBC (Spearman Rho = 0.14) Fig. 5). We identifies significantly more functionally enriched pathways overall in the epithelial-epithelial co-expression network in ER-positive IBC (n = 86 with FDR < 5 %) as compared with the epithelial-stromal co-expression network in ER-positive IBC (n = 40) (*p* = 3.8e-5), with no significant differences in numbers of significant pathways in the epithelial-epithelial versus the epithelial-stromal networks in ER-negative (*p =* 1) or normal breast (*p* = 0.25). Overall, there was significant positive association of pathways identified by the epithelial-epithelial analysis and epithelial-stromal analysis in ER-positive breast cancer (OR = 49.5, *p* < 2.2e-16; Fig. 5), with no significant positive association between pathways identified in the epithelial-stromal versus epithelial-epithelial in ER-negative IBC or normal breast (both *p* > 0.21).

For the prognostic signatures, there was no significant difference in the numbers of genesets identified as significantly enriched in the ER-positive epithelial-stromal network versus the epithelial-epithelial network (*p* = 0.62); however, we did identify significantly more prognostic signatures as enriched in the ER-negative epithelial-stromal co-expression network (19/125, 15 %), as compared with the ER-negative epithelial-epithelial co-expression network (5/125, 4 %) (*p* = 0.005) (Additional file 9). In particular, in ER-negative IBC, there was strong and specific enrichment for a core embryonic stem cell module [75] and for a genomic grade signature [76] and a genomic instability signature [77] in the epithelial-stromal co-expression network, with no enrichment for these pathways in the epithelial-epithelial co-expression network. In ER-positive IBC, the genomic grade index and chromosomal instability signatures were both significantly enriched in both the epithelial-epithelial and epithelial-stromal networks.

We also identified a subset of pathways that showed significant enrichment in the epithelial-epithelial networks, but not the epithelial-stromal networks (Fig. 5). Interestingly, a signature initially defined by expression in a fibroblastic neoplasm (the fibromatosis signature [4]) was the most highly enriched signature in the epithelial-epithelial co-expression networks in both ER-positive and ER-negative IBC and showed no significant enrichment in the epithelial-stromal co-expression networks (Fig. 5). Similarly, the macrophage CSF1-response signature [5] was highly enriched in the ER-negative IBC epithelial-epithelial network, but showed no significant enrichment in the ER-negative IBC epithelial-stromal network. These data suggest that in IBC, stromal-derived gene signatures may become activated within the epithelial compartment of breast cancer, consistent with a process of epithelial-to-mesenchymal transition.

### Epithelial-stromal self-loop co-expression relationships are increased in breast cancer

We defined an epithelial-stromal self-loop co-expression relationship to be a gene expression pattern in which a gene’s epithelial expression level was correlated with the same gene’s stromal expression level. Based on this definition, the overall background frequency of self-loop relationships was less than 0.01 %. Among statistically significant co-expression relationships (FDR < 5 %), we identified a much larger proportion of self-loops in ER-positive IBC (332/1746, 19.0 %) and ER-negative IBC (210/2441, 8.6 %) as compared with normal breast (5/965, 0.52 %) (all *p* < 2.2e-16) (Fig. 2). These data suggest that the emergence of epithelial-stromal self-loop relationships is a characteristic feature of the rewiring of epithelial-stromal co-expression networks that occurs in breast carcinogenesis.

The most statistically significant epithelial-stromal co-expression relationships in IBC tended to be self-loop relationships, with 89 % and 75 % of relationships significant at FDR < 0.0001 representing self-loops in ER-positive and ER-negative IBC, respectively, compared with only 8 % and 2 % of relationships with an FDR between 10 % and 1 % (both *p*-values for trend < 2.2e-16) (Fig. 2). These data show that epithelial-stromal self-loop interactions tend to show the largest univariate significance levels. To further evaluate the role of epithelial-stromal self-loop nodes in the overall network, we compared the network degree of self-loop genes to the network degree of the non-self-loop genes. These data show that self-loop genes tend to be significantly more connected in the networks as compared with non-self-loop genes (all *p* < 2.2 e-16) (Additional file 10). We assessed the overlap in self-loop genes identified in ER-positive IBC and ER-negative IBC, and we identified a significant positive enrichment (OR = 4.4, *p* < 2.2e-16), with 89 genes identified as self-loops in both the ER-positive and ER-negative IBC networks. There was no significant overlap between self-loop genes identified in normal breast and self-loop genes in the ER-positive or ER-negative IBC networks (both *p* > 0.19).

Genes with the strongest self-loop relationships in ER-negative IBC included *ORM1*, *PCP4*, *MMP10*, *DSC3*, *IMPA2*, *ASPM*, *LCN2*, *KRT16*, and *SH3GL2* (Table 1). Genes with the strongest self-loop relationships in ER-positive IBC included *CEACAM5*, *S100A7*, *FAM5C*, *BEX1*, *IFIH1*, *AGT*, *BAMBI*, *PDH8*, *S100A8*, and *ATHL1* (Table 1). The self-loop status of each gene in each of the three epithelial-stromal co-expression networks is included in Additional file 6.

### Evaluation of epithelial-stromal self-loops with breast cancer-fibroblast co-culture data

Co-culture of breast cancer cells with fibroblasts is an in vitro system that has been widely used for studying the effects of direct physical interactions of breast cancer cells with fibroblasts [78–80]. To determine an association between epithelial-stromal self-loops and changes in breast cancer epithelial cells and fibroblasts induced by direct physical interactions, we used a publicly available list of genes identified by Camp and colleagues [81] in a gene expression profiling-based study as induced in both breast cancer cells and fibroblasts upon breast cancer cell-fibroblast co-culture. We observed a significant overlap between genes predicted to be self-loops by our epithelial-stromal co-expression analyses (in ER-positive IBC and/or ER-negative IBC) and genes identified as up-regulated in breast cancer cell lines following co-culture with fibroblast cell lines (OR = 2.33, Fisher’s exact *p* = 0.002), with 19 genes (*IFI30*, *S100A8*, *S100A9*, *FABP5*, *SEMA3F*, *S100P*, *TAP1*, *TGFB3*, *IMPA2*, *LCN2*, *CYP2J2*, *OAS2*, *DUSP1*, *IFIH1*, *SERPINA3*, *SAMD9*, *DDX58*, *IL1R2*, *ASRGL1*) identified by both the epithelial-stromal co-expression self-loop analysis and the breast cancer-fibroblast co-culture in vitro study.

### Evaluation of an independent LCM dataset in ER-positive invasive breast cancer

To validate our findings on an independent set of samples, we obtained epithelial and stromal LCM-derived gene expression profiling data from a total of 36 cases of ER-positive IBC from McGill University. We performed an epithelial-stromal co-expression analysis (as described above) on this independent dataset. To assess network concordance between our original ER-positive IBC dataset and the ER-positive IBC dataset from McGill University, we applied a raw *p*-value cutoff of 0.001 to identify significant network edges and then assessed concordance in the sign of association among edges identified at this threshold in both datasets. Using this approach, we identified strong concordance in network predictions, with a Spearman correlation of network edge t-statistics of 0.44 (*p* < 2.2e-16) (Additional file 11), with 981 of the 1142 edges identified in both datasets showing concordant direction (86 %, *p* < 2.2e-16). In agreement with our earlier findings, in the McGill University dataset, there was a strong positive association of the proportion of self-loop interactions and the statistical significance of network edges, with an increasing proportion of self-loops among the most significant network edges (Additional file 12), and we identified significant concordance in predicted self-loops in the two datasets, with an eightfold increase in the proportion of self-loops in the McGill University dataset among genes predicted by the primary analysis to be self-loops, as compared with genes not predicted in the primary analysis to be self-loops (*p* < 2.2e-16).

### Pathological evaluation of epithelial-stromal self-loop co-expression relationships by immunohistochemistry on tissue microarrays

**Fig. 6:**
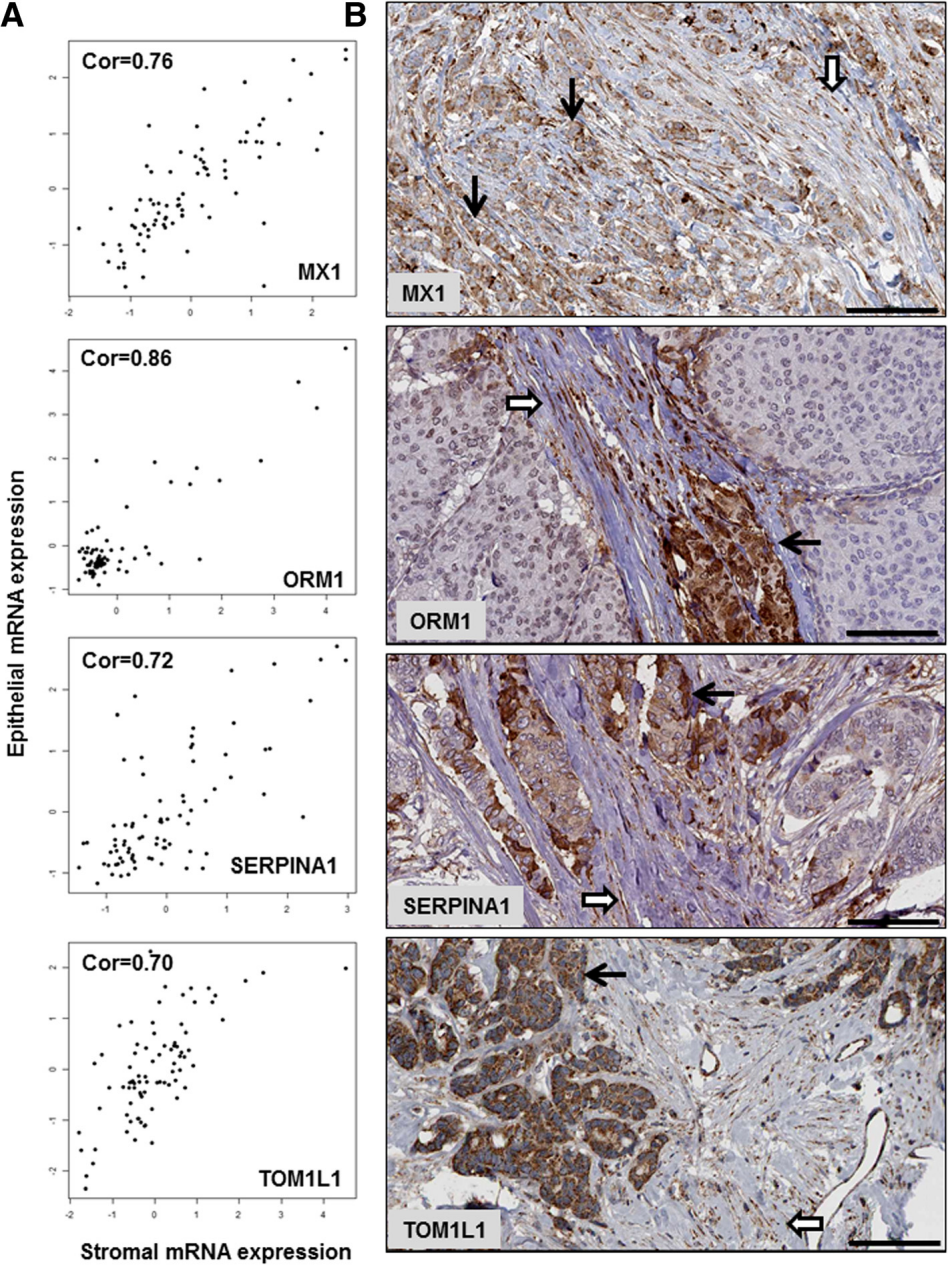
Genes predicted to be involved in epithelial-stromal self-loops show coordinate epithelial and stromal protein expression by immunohistochemistry. **a** Scatterplots of mRNA expression of *MX1*, *ORM1*, *SERPINA1*, and *TOM1L1* in epithelium and stroma of 82 invasive breast carcinomas from LCM-derived gene expression data. The epithelial and stromal expression of each gene is positively correlated in cancer. **b** Protein expression of MX1, ORM1, SERPINA1, and TOM1L1 is concurrently seen in cancer epithelium and stromal spindle cells in images from the Human Protein Atlas. Stronger protein expression is often seen at the periphery of tumor nests and at the tumor-stroma interface for each protein marker (*black bar* 100 um, *black arrow* cancer epithelium; *open arrow* stromal cells)

To evaluate the epithelial-stromal self-loop relationships by an additional approach on independent samples, we identified the 38 proteins predicted by the epithelial-stromal co-expression network to be involved in statistically significant epithelial-stromal self-loops (FDR < 5 %) in both ER-positive and ER-negative IBC and not in normal breast. For each of these 38 proteins, we used the Human Protein Atlas [51] to identify images of normal breast and breast cancer tissue microarrays (TMAs) stained for the protein by immunohistochemistry. For each marker, a pathologist (EYO) separately assessed the protein’s expression in the epithelium and stroma (Additional file 13). Out of the 38 proteins, four antibodies were unavailable and one antibody (COL2A1) was uninterpretable. Thus, immunohistochemistry images for a total of 33 antibodies were analyzed, including a total of 72 cores of benign breast tissue (median of two cores per antibody), and a total of 276 cores of breast cancer (median of eight cores per antibody). Of the 33 evaluable antibodies, four antibodies (UGT2B28, SLPI, SULT4A1,VGLL1) failed to show staining in the epithelium or stroma from any of the tumor or benign breast tissue cores. Of the remaining 29 antibodies, 24 (83 %) showed positive staining in both the epithelial and stromal compartments of the invasive breast carcinoma cores compared with only 13 (45 %) showing positive epithelial and stromal staining in benign breast tissue (*p* = 0.006). Of the 24 antibodies that showed positive epithelial and stromal expression in invasive breast cancer, 18 (75 %) showed coordinated epithelial and stromal expression in at least 50 % of the evaluated cores, and nine (38 %) showed coordinated epithelial and stromal expression in all evaluated cores (Fig. 6). Of the 13 proteins that showed positive epithelial and stromal staining in benign breast tissue, six proteins were expressed in the breast epithelium and stromal endothelial cells without staining of the stromal spindled (fibroblastic) cells. This pattern of exclusive stromal endothelial staining was not observed in the positive epithelial-stromal cancer cases, in which stromal staining included at least focal spindle cell staining in each positive case. Taken together, these results support that the emergence of epithelial-stromal self-loops represents an important property of the rewiring of epithelial-stromal co-expression networks that occurs in carcinogenesis.

### Computational evaluation of epithelial-stromal self-loop co-expression relationships by immunohistochemistry on tissue microarrays

As an alternative and complementary approach for unbiased estimation of epithelial-stromal self-loop interactions, an automated image-processing strategy was designed and applied to a large set of TMA images from the Human Protein Atlas [51, 52]. Specifically, we evaluated all available proteins for the genes listed in Table 1 for ER-positive IBC, ER-negative IBC, and normal breast, as well as the top 50 most connected genes (with the largest degree) from the ER-positive IBC, ER-negative IBC, and normal breast epithelial stromal networks in both the normal and breast cancer samples from the Human Protein Atlas. Up to six TMA images were analyzed for each protein in each of normal breast and breast cancer. We wrote a custom R script to perform bulk download of images from the Human Protein Atlas (Additional file 14). Overall, we evaluated 1147 images from a common set of 105 proteins in normal breast (n = 475 images) and breast cancer (n = 672 images) from the Human Protein Atlas.

Each immunohistochemistry image was processed using an automated image analysis pipeline to extract the proportion of pixels stained brown in the epithelium and stroma (Additional file 15). To achieve this goal, the image was first divided into superpixels. Superpixels are locally smooth regions into which an image can be partitioned based on local intensity and edge statistics. Next, each superpixel was identified as either belonging to epithelium or stroma. Superpixels that contained background and fat were discarded from segmentation based on a simple intensity threshold. A support vector machine (SVM) algorithm was used to predict the class (epithelium or stroma) of each superpixel. Texture features were used to train the SVM classifier. Specifically, the texture features extracted for each superpixel were correlation, contrast, dissimilarity, homogeneity, and local binary pattern. The proportion of pixels with brown stain in the epithelium and the stroma were reported and analyzed for validation (Additional file 16).

**Fig. 7:**
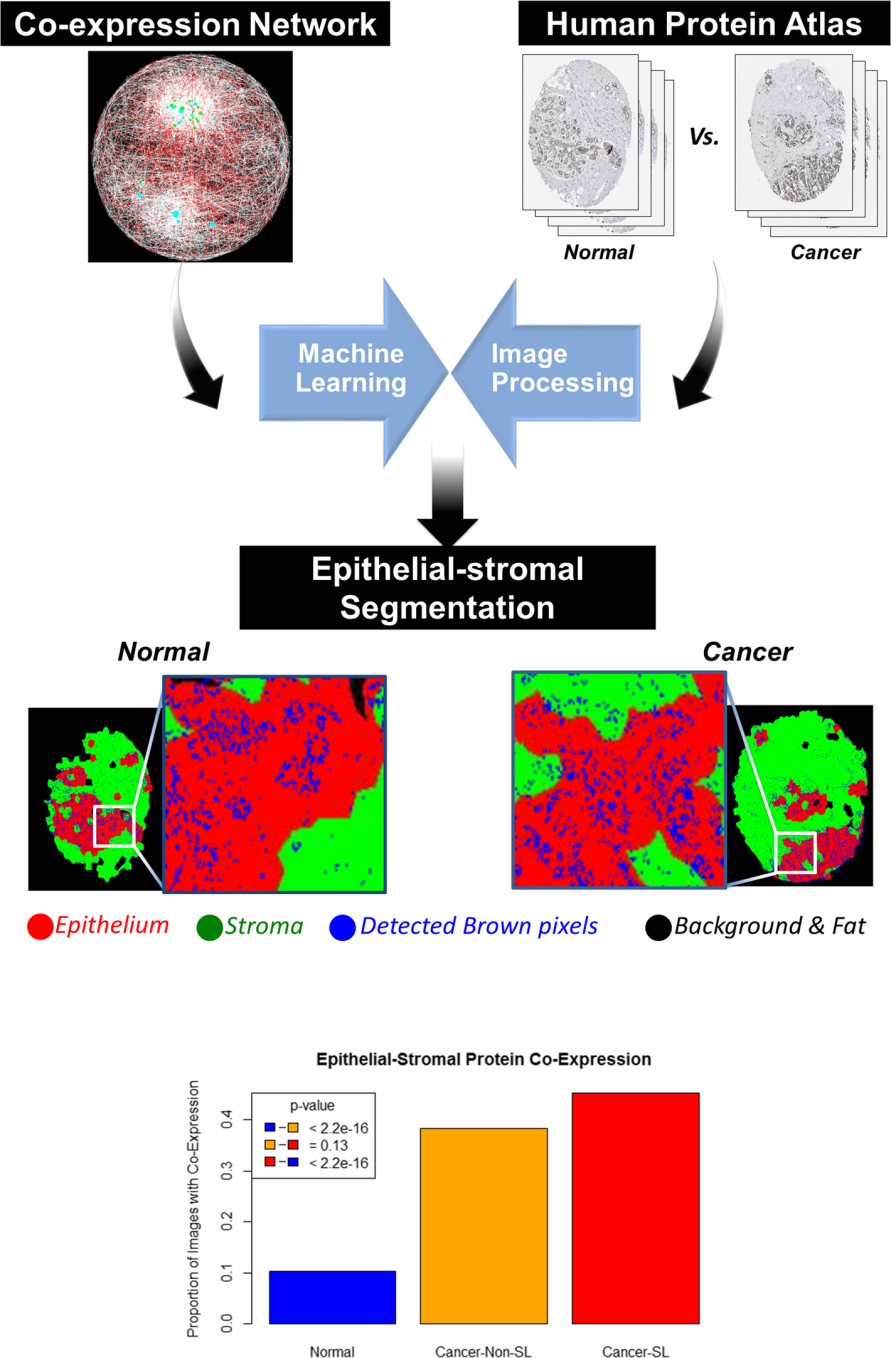
Assessment of protein co-expression in the epithelium and stroma by computational image analysis. We performed a large-scale validation experiment of predicted cancer self-loops by evaluating 1147 images from a common set of 105 proteins in normal breast and breast cancer. We then performed machine learning-based epithelial-stromal segmentation followed by quantitation of protein expression in the epithelium and stroma. *Red* indicates epithelium and *green* indicates stroma. Pixels whose class was either unknown or which did not belong to either of the classes are represented in *black*. After this, pixels containing brown stain in each region were extracted by applying a threshold to the intensity values in the red, green, and blue channels of the image. Brown pixels belonging to epithelium or stroma were reported and analyzed for validation. The analysis shows significantly increased epithelial-stromal protein co-expression in breast cancer as compared with normal breast, as predicted by our network analysis (*p* < 2.2e-16). There was a trend for increased epithelial-stromal co-expression for predicted self-loops within the cancer samples, although this did not reach statistical significance (45 % vs 38 %, *p* = 0.13). *non-SL non-self-loop; SL self-loop*

After computing the proportion of epithelial and stromal pixels with protein staining, we classified the epithelial and stromal stains as positive or negative by performing univariate Gaussian mixture model-based clustering with two clusters (“negative” and “positive”) separately for the epithelial and stromal protein expression scores. A protein was classified as co-expressed in the epithelium and stroma if the protein was in the positive cluster in both the epithelium and stroma. This analysis showed a significant increase in self-loops in breast cancer as compared with normal breast tissue, with epithelial-stromal protein co-expression in breast cancer for 45 % of proteins predicted by the network analysis to be self-loops and for 38 % of proteins not predicted to be self-loops, as compared with only 10 % of proteins in normal samples (Fig. 7). Thus, this analysis supports significantly increased epithelial-stromal protein co-expression in breast cancer as compared with normal breast (*p* < 2.2e-16). There was a trend for increased epithelial-stromal co-expression for predicted self-loops versus the non-predicted self-loops within the cancer samples, although this trend did not obtain statistical significance (45 % vs 38 %, *p* = 0.13).

## Discussion

Over the past two decades, there has been increasing appreciation of the importance of epithelial-stromal interactions in supporting initiation, progression, metastasis, and drug resistance in solid tumors [15, 17, 19, 24]. However, little is known on a systems level of how global patterns of epithelial-stromal interactions evolve during carcinogenesis. Thus, we developed a computational approach for building and analyzing genome-wide epithelial-stromal co-expression networks using transcriptional profiling data obtained from matched epithelial and stromal samples.

Co-expression networks have been widely used in studies of cancer (e.g., to identify prognostic signatures [49] and to uncover cellular phenotypes in the tumor microenvironment from bulk expression profiling data [50]). Further, the critical role of stromal gene expression patterns in determining patient prognosis is now well-established and has been demonstrated in many cancer types, including breast, colorectal, and lymphoma [7, 9, 12]. However, no prior studies have used co-expression networks to directly assess the evolution of coordinated patterns of epithelial-stromal gene expression genome-wide in cancer.

Our analysis of genome-wide epithelial-stromal co-expression networks showed epithelial-stromal co-expression network self-loops to be highly enriched among the most significant interactions in IBC, and we validated by immunohistochemistry that epithelial-stromal self-loop co-expression relationships are much more common in IBC than in normal breast tissue. Our analysis identified significant functional rewiring of epithelial-stromal co-expression networks in IBC as compared with normal breast, with the emergence of network enrichment for prognostic signatures in the IBC epithelial-stromal networks as compared with the normal breast epithelial-stromal co-expression network.

Limitations of our study include the fact that our networks were based on correlation of mRNA expression levels. Although a large body of work has supported the effectiveness of studying networks of co-expression interactions to prioritize functionally related genes and biological modules [47, 82–84], it is critical to note that most correlations observed in large scale Omics datasets do not represent functional interactions but instead represent indirect, or “passenger,” interactions. Thus, although the analysis of co-expression networks is a useful approach for uncovering new co-expression relationships and prioritizing hubs most likely to be important to the network, discriminating truly functionally interacting molecules from indirect correlations will require future hypothesis-driven mechanistic studies to functionally validate hypotheses generated from this work. These studies could include a variety of pre-clinical epithelial-stromal in vitro and in vivo modeling approaches, including 2D and 3D co-culture systems [85] and genetically engineered mouse models. These approaches could be leveraged to determine the biological mechanisms producing the patterns of epithelial-stromal co-expression observed in our analyses. These potential mechanisms include cancer cell to stromal cell physical interactions; cancer cell and stromal cell interactions with the extracellular matrix; and cancer cell and stromal cell response to secreted factors, such as cytokines, adipokines, proteases, angiogenic factors, growth factors [13, 16], and exosome transfer [86, 87].

Additional limitations of the study include the fact that the primary analysis was based entirely on LCM-derived data from the epithelium and stroma. The stroma contains a variety of different cell types, including smooth muscle cells, fibroblasts, endothelial cells, perictyes, and adipocytes. In our bulk stromal tissue analyses, stromal expression values represent a summary of expression from these heterogeneous cell types and our analyses were unable to account for differences in stromal cell type proportions between samples. In principle, future studies could address these limitations by applying spatially resolved transcriptome profiling methods to enable the assessment of stromal expression patterns with single cell resolution [31–34].

Beyond functional validation of network components and application of spatially resolved single cell transcriptome measurements, future work can build on our analysis in several additional directions. The first direction is to generate epithelial-stromal co-expression networks from larger sample sizes spanning diverse cancer types. Recently developed molecular methods for spatially resolved transcriptomics [34] and highly multiplexed next-generation immunohistochemistry [35] may enable the generation of large, spatially resolved cancer expression profiling datasets. The generation of these datasets across diverse clinically annotated cancer samples will enable the construction of well-powered epithelial-stromal co-expression networks and permit comparative analyses of epithelial-stromal crosstalk networks, network functional enrichment, and network hubs across human cancers.

A second important future direction is to integrate additional Omics data types (e.g., epigenetic profiling, copy number profiling, mutation profiling) into the epithelial-stromal co-expression network analytic framework to enable identification of the genetic and epigenetic etiology of patterns of epithelial-stromal co-expression network connectivity. This general approach has proven to be a powerful method for studying the genetics of gene expression [88, 89], and we expect integrative analyses of tissue region-specific genetics and gene expression will provide important insights into the genetic basis of epithelial-stromal co-expression networks in cancer.

Third, constructing epithelial-stromal co-expression networks from epithelial and stromal samples obtained from pre-invasive neoplasia (e.g., atypical ductal hyperplasia and ductal carcinoma in situ) will allow the characterization of temporal changes in epithelial-stromal co-expression network connectivity during the longitudinal process of carcinogenesis. There is strong evidence that stromal changes precede the development of IBC and several candidate mediators of this process have been described [14, 90]. However, no prior studies to date have systematically analyzed the evolution of epithelial-stromal co-expression relationships on a genome-wide scale in early neoplasia.

Ultimately, we hope that the systematic characterization of epithelial-stromal co-expression relationships will lead to the identification of drivers of epithelial-stromal crosstalk and to the development of new epithelial-stromal network-derived diagnostics and therapeutics, aimed at monitoring and targeting epithelial-stromal interactions for early detection, diagnosis, and treatment of cancer.

## Conclusions

We developed an approach for constructing and analyzing epithelial-stromal co-expression networks in normal breast and breast cancer. Our network analysis identifies a major increase in the number of epithelial-stromal self-loops in breast cancer samples, and we validated the co-ordinate epithelial and stromal expression of a subset of self-loop proteins by analysis of immunohistochemistry data. Our analysis provides new biological insights into the functional rewiring of epithelial-stromal co-expression networks in breast cancer. The approach may facilitate the development of new diagnostics and therapeutics targeting epithelial-stromal interactions in cancer.

## Methods

### Datasets

The expression profiling data from pairs of epithelial-stromal samples in our study were derived from five published datasets [39–41, 43, 54] available from the NCBI GEO database [GEO:GSE4823, GEO:GSE5847, GEO:GSE10797, GEO:GSE14548, and GEO:GSE35019]. The GSE4823 dataset contains 22 matched epithelial and stromal samples from histologically normal breast [40], which were profiled using the Agilent Whole Human Genome Oligo Microarray G4112A platform. The study included replicate arrays for each sample, which were averaged prior to inclusion in our study. The GSE5847 dataset consisted of 34 sets of matched epithelial and stromal samples obtained from non-inflammatory breast carcinomas (30 ductal and 4 lobular) and 15 sets of matched epithelial and stromal samples captured from the inflammatory breast carcinoma [39], which were profiled using the Affymetrix U133A 2.0 platform. The inflammatory breast carcinoma cases were excluded from our analysis. The GSE10797 dataset consisted of 28 sets of matched epithelial and stromal samples from invasive breast carcinomas (25 ductal and 3 lobular) and five matched epithelial and stromal samples from histologically normal breast, all profiled by the Affymetrix U133A2.0 platform [43]. The GSE14548 dataset consisted of 14 sets of matched epithelial and stromal samples obtained from invasive ductal carcinoma and profiled on the Affymetrix U133X3P platform [54]. The GSE35019 dataset consisted of 11 sets of matched epithelial and stromal samples obtained from invasive ductal carcinoma, all profiled using the Whole Genome DASL platform [41]. The method for dataset integration is described in the Results section “Assembly of an LCM dataset of paired epithelial and stromal samples in normal breast, ER-negative invasive breast cancer, and ER-positive invasive breast cancer”. The datasets used in the analysis are provided in Additional file 17.

### Computation of epithelial-stromal co-expression interactions

For each of the three datasets, we used the MatrixEQTL package to compute all pairwise associations of epithelial and stromal gene expression levels and to estimate FDRs [57]. The FDR computation with MatrixEQTL accounted for the full set of hypotheses analyzed, but for computational speed, FDRs were only reported for interactions achieving a raw *p*-value less than 0.001. The full listing of all interactions that achieved this raw *p*-value threshold is provided in Additional file 18. The same approach was applied for computing the epithelial-epithelial co-expression interactions, with the exception that the self-interactions were removed from the epithelial-epithelial networks.

### Network analysis

For each of normal breast, ER-positive IBC, and ER-negative IBC, we selected the 10,000 most significant co-expression relationships for network analyses. We used the *iGraph* package in R [58] to compute each node’s epithelial and stromal degree in each network. Network visualization was performed with the RedeR package in R [59]. Functional network enrichment analyses were performed with the SANTA package in R using default parameters [60].

### Processing of validation LCM dataset

The normalization of the validation dataset is described in [40]. Specifically, microarray data were feature extracted using Feature Extraction Software (v. 7.11) from Agilent with the default parameters. Raw data were uploaded to the NCBI GEO database and are accessible as data series [GEO:GSE68744]. Outlier features on arrays were flagged by the software. Arrays were required to have an average raw signal intensity of 1000 in each channel, and a signal to noise ratio above 16 per channel. MvA plots were examined for signs of hybridization or labeling problems. Replicate arrays were required to have a concordance above 0.944. This level was established empirically using sets of known good replicate arrays in our database. Data preprocessing and normalization were automated using the BIAS system [91]. Raw feature intensities were background corrected using the RMA background correction algorithm [92, 93]. Resulting expression estimates were converted to log2-ratios. Within-array normalization was performed using spatial and intensity-dependent loess [94]. Median absolute deviation scale normalization was used to normalize between arrays [95]. The normalized data used in our analyses is provided in Additional file 19.

### Validation of predicted epithelial-stromal self-loops by manual pathological assessment of immunohistochemistry images from the Human Protein Atlas

To validate predicted epithelial-stromal self-loop interactions by an orthogonal and in situ approach, we used the large collection of protein immunohistochemistry images from normal and cancer tissue available at the Human Protein Atlas, which is a publicly available database with millions of high-resolution images showing the spatial distribution of proteins in 44 different normal human tissues and 20 different cancer types, including breast tissue and breast cancer [51]. For each of the 38 genes included in the protein atlas and predicted by our analysis as contributing to epithelial-stromal self-loops in both ER-positive IBC and ER-negative IBC at an FDR < 5 %, we evaluated the gene’s protein expression in the epithelium and stroma of normal breast tissue and breast cancer. Information on the antibodies used and the pathological interpretation of the immunohistochemistry studies is provided in Additional file 13.

### Validation of predicted epithelial-stromal self-loops by computational image analysis assessment of immunohistochemistry images from the human protein atlas

#### Image preparation

We downloaded images from the Human Protein Atlas using a custom-designed R-script (Additional file 14). The script searches for information based on a user-defined query (i.e., gene name) and downloads the corresponding images and meta data (e.g., antibody stain, patients, and disease information) from all four categories in the Human Protein Atlas: tissue atlas, subcell atlas, cell line atlas, and cancer atlas. The script was tested and optimized on Windows, Linux, and LSF cluster machine. As described in the results, we used the script to download a total of 1147 images from a common set of 105 proteins in normal breast (n = 475 images) and breast cancer (n = 672 images) from the Human Protein Atlas.

#### Image analysis

We wrote an automated image analysis script in Python to classify each image into epithelial and stromal regions using a SVM and to compute the proportion of positively staining pixels in the epithelium and in the stroma. The image-processing script and the results of the immunohistochemistry quantification are provided as Additional files 14 and 15, respectively.

#### Knitr file to reproduce primary analyses

A knitr file (.rnw) and the resulting .pdf file are provided with the complete R code to reproduce the primary analyses and figures from the manuscript (Additional file 20).

### Data access

The data from this study is provided in Additional files. In addition, the microarray datasets are available from GEO under the listed accession numbers [GEO:GSE4823, GEO:GSE5847, GEO:GSE10797, GEO:GSE14548, and GEO:GSE35019]. Raw data from the McGill University validation dataset were uploaded to the NCBI GEO database and are accessible as data series [GEO:GSE68744].

### Ethical approvals

The McGill University validation dataset was approved by the McGill University Health Centre Research Ethics Board (protocols SDR-99-780 and SDR-00-966). All participants provided written informed consent, and all use of human samples was performed in accordance with the Declaration of Helsinki.

## Additional files

Additional file 1:
**Association of dataset with** ***ESR1*** **status.** The *y-axis* indicates the *ESR1* epithelial mRNA level. Cases of invasive breast cancer are grouped along the *x-axis* and colored according to the dataset the sample is derived from (blue = GSE10797, black = GSE14548, green = GSE35019, red = GSE5847). The shape of each object in the plot indicates whether it was classified as ESR1-positive (*triangle*) or ESR1-negative (*circle*). This plot provides no striking evidence of batch effect effecting ESR1 status, with no significant association between the ESR1 classification (positive vs negative) and dataset site (*P* = 0.34). Additional file 2:
**Hierarchical clustering relationship with dataset.** We performed unsupervised hierarchical clustering of the normal breast (*top panel*), ER-positive IBC samples (*middle panel*), and ER-negative IBC samples (*bottom panel*) from the epithelium (*left panels*) and the stroma (*right panels*). We performed clustering with Euclidean distance and complete linkage. Each leaf in the dendrogram is labeled with the dataset the sample came from (normal 5 = GSE10797, 14 = GSE14548, 22 = GSE4823; IBC 9 = GSE14548, 11 = GSE35019, 28 = GSE10797, 34 = GSE5847). Additional file 3:
**Scatterplots of first two principal components with points colored by dataset.** Each plot displays a scatterplot of a sample along the first two principal components for normal breast (*top panel*), ER-positive IBC samples (*middle panel*), and ER-negative IBC samples (*bottom panel*) from the epithelium (*left panels*) and the stroma (*right panels*). The color of each sample indicates the dataset it comes from (normal red = GSE14548, green = GSE4823, black = GSE10797; IBC blue = GSE10797, black = GSE14548, green = GSE35019, red = GSE5847). Additional file 4:
**Permutation experiment to assess impact of shuffling dataset on the concordance of co-expression analyses across sites.** Barplots on the left indicate the observed fraction of sign reversed correlations on the true data (*Labels Not Shuffled*) as compared with the median fraction of sign reversed correlations with the *Labels Shuffled*, when the epithelial-stromal co-expression analysis was performed separately on the two largest datasets for normal breast (*top panel*), ER-positive IBC (*middle panel*), and ER-negative IBC (*lower panel*). The histograms on the *right* show the distribution of the sign-reversed correlations across 100 iterations. The observed sign-reversed correlation fraction with the true dataset labels is indicated with a *red arrow*. In normal breast, there is strong evidence of batch effect, while there is no evidence of significant batch effect in ER-positive and ER-negative IBC. Additional file 5:
**Hierarchical clustering of largest normal breast dataset shows strong concordance between technical replicates.** We performed unsupervised hierarchical clustering of the technical replicate (dye-swap) normal samples from GSE4823. There is strong concordance for each of the technical replicates, and for each sample technical replicates show the strongest correlation with each other. Additional file 6:
**Epithelial-stromal co-expression network node degree matrix.** This data table presents each gene’s overall degree, epithelial- and stromal-specific degree, and presence of self-loops in the normal, ER-positive IBC, and ER-negative IBC networks. The column *Normal Degree* indicates the row’s gene’s overall degree in the normal breast epithelial stromal cross-talk network. The columns *Stroma Normal Degree* and *Epi Normal Degree* indicate the number of connections derived from stromal or epithelial expression in the normal network, respectively. The column *Normal Self-loop* contains a 1 if the row’s gene is involved in a self-loop interaction in the normal breast network, and a 0 otherwise. The remaining columns follow the same naming conventions for the ER-positive IBC and ER-negative IBC epithelial-stromal crosstalk networks. Additional file 7:
**Genesets used in the functional geneset enrichment analyses.** This zip directory contains four files, c2.cp.kegg.v4.0.symbols.gmt, c5.bp.v4.symbols.gmt, CellTypeSpecificSignatures.txt, and PrognosticSignatures.txt, which contain genesets for the KEGG biological pathways, GO biological processes, cell type-specific signatures, and breast cancer prognostic signatures, respectively. Additional file 8:
**SANTA functional network enrichment analysis results.** This file contains the functional network enrichment analysis results. Each *row* indicates a geneset, and the *columns* contain results for each network connotation Each *cell* in the matrix is the adjusted *p*-value (FDR) for the row’s geneset in the column’s network. The genesets derive from four collections: breast cancer prognostic signatures (BRCA_PROG_SIG) [74], a collected set of cell type-specific signatures (CELL_TYPE_SPEC), Gene Ontology biological processes (GO_BP) [72], and KEGG biological pathways (KEGG) [73]. Additional file 9:
**Proportion of genesets identified as significantly enriched in the epithelial-epithelial and epithelial-stromal co-expression networks according to geneset category.** The *y-axis* indicates the proportion of overall genesets enriched in the normal, ER-positive IBC, and ER-negative IBC networks. The *red bars* indicate the epithelial-epithelial co-expression network and the *blue bars* indicate the epithelial-stromal co-expression network. **A-C** show the overall genesets, GO biological process genesets, and breast cancer prognostic signature genesets, respectively. Additional file 10:
**Epithelial-stromal self-loops are significantly more connected in the epithelial-stromal networks than non-self-loops.** The boxplots display the distribution of node degree for self-loop genes (*SL*) and non-self-loop genes (*No-SL*) in normal breast, ER-positive IBC, and ER-negative IBC epithelial-stromal co-expression networks. The median node degree is significantly higher for the self-loops in each of the three networks (all *p* < 2.2e-16). Additional file 11:
**Scatterplot of epithelial-stromal T-statistics from the McGill ER-positive IBC LCM dataset and the original meta-ER-positive IBC dataset.** Each *point* represents an epithelial-stromal co-expression relationship, which achieved a raw *p* < 0.001 in the epithelial-stromal co-expression analysis. The *x-axis* indicates the T-statistic in the ER-positive IBC meta-dataset, and the *y-axis* indicates the T-statistic on the McGill ER-positive IBC dataset. The Spearman correlation is 0.44 (*p* < 2.2e-16). Additional file 12:
**Self-loops tend to have more significant edges in the McGill ER-positive IBC LCM dataset.** The *y-axis* indicates the proportion of epithelial-stromal self-loops. The groups on the *x-axis* indicate significance windows for the epithelial-stromal interactions, ranging from most significant (−log(fdr) > 5) to the least significant (−log(fdr) < 3). Additional file 13:
**Results from the manual pathology evaluation of predicted self-loop proteins by immunohistochemistry.** Each *row* represents an evaluated protein. *Column A* indicates the protein’s gene symbol. *Column B* indicates the Antibody ID from the Human Protein Atlas. *Columns C–E* contain information from benign breast tissue, and columns *F–J* contain information from invasive breast cancer. *N* indicates the number of evaluable cores. The *Pos* column indicates the number of positive cores. The positive cases are coded as *E*, epithelium only; *EE*, epithelium and endothelial cells; *ES*, epithelium and stroma; and *S*, stroma only. (CSV 2 kb) Additional file 14:
**R-scripts for downloading images from the Human Protein Atlas.** The zip directory contains two r scripts: the r script hpa.r is used for downloading images from the Human Protein Atlas and an example use of the script is provided in hpa_examples.r. Additional file 15:
**Python script for analyzing the HPA immunohistochemistry images.** This zip directory contains two python files: brown_stain_extraction.py and RGB_SVM.pk1. The image-processing code and its description are provided in the *.py file and the RGB_SVM.pk1 file contains the model parameters. Additional file 16:
**Results of computational image analysis of epithelial and stromal protein expression in the Human Protein Atlas.** The zip directory contains two files, Cancer.HPA.csv and Normal.HPA.csv, which contain the results of applying the script to quantitate protein expression in the epithelium and stroma of cancer and normal samples, respectively. Each output file contains the following columns: *Stroma.pixels* and *Epithelium.pixels* (indicating the total number of pixels in the stroma and epithelium, respectively); *Brown.Spots.in.Stroma* and *Brown.Spots.in.Epithelium* (indicating the number of positively stained pixels in the epithelium and stroma, respectively); and *Nucleus.Pixels.in.Stroma* and *Nucleus.pixels.in.Epithelium* (indicating the number of pixels classified as nucleus in the stroma and epithelium, respectively). Additional file 17:
**The expression data for each sample from GEO used in our analyses.** This zip directory contains ten text files, each labeled with the dataset’s GEO series identifier, an indicator of whether the data is from normal (*No*) or breast cancer (*Br*), an indicator of whether the data is from the epithelium (*Epi*) or stroma (*Str*), and the number of samples in the dataset. Additional file 18:
**Epithelial-stromal co-expression.** This zip directory contains three files: Normal_22_ES.txt, ER_Positive_ES.txt, and ER_Negative_ES.txt. Each file is a tab-delimited table containing the epithelial-stromal interactions that achieved a raw *p*-value of 1e-3 in the epithelial-stromal co-expression network indicated by the file’s name (Normal, ER-positive IBC, ER-negative IBC). Each *row* indicates an epithelial-stromal interaction. The *first column* indicates the gene expressed in the stroma, the *second column* indicates the gene expressed in the epithelium, the *third column* indicates the interaction’s T-statistic, the *fourth column* indicates the raw *p*-value associated with the T-statistic, and the *fifth column* indicates the interaction’s FDR. Additional file 19:
**Normalized gene expression data from paired epithelial and stromal samples in the McGill University validation dataset.** This zip directory contains two files, eset_erp_finak_anno.txt and eset_erp_finak_ex.txt, which contain the gene annotation and gene expression data, respectively. The gene expression matrix contains normalized expression values, with tumor epithelial (*TE*) data for 36 patients in the first 36 data columns, and tumor stromal (*TS*) data for the same 36 patients in the remaining 36 columns. Additional file 20:
**Knitr script for performing statistical analyses in R (analysis.knit.zip).** This zip directory contains two files, analysis.rnw and analysis.pdf, for running the main statistical analyses from the paper in R (analysis.rnw) and for producing a file containing both the code and the results and figures from the analyses (analysis.pdf).

## Abbreviations

ER: Estrogen receptor
FDR: False discovery rate
IBC: Invasive breast cancer
LCM: Laser capture microdissection
SVM: Support vector machine
TMA: Tissue microarrays
