# Supplementary figures and images for: Extensive rewiring of epithelial-stromal co-expression networks in breast cancer
Source: Genome Biol. 2015 Jun 19;16(1):128. doi: 10.1186/s13059-015-0675-4 (PMC4471934; doi:10.1186/s13059-015-0675-4)

# No Significant Association of Dataset with ESR1 Status (P=0.34)

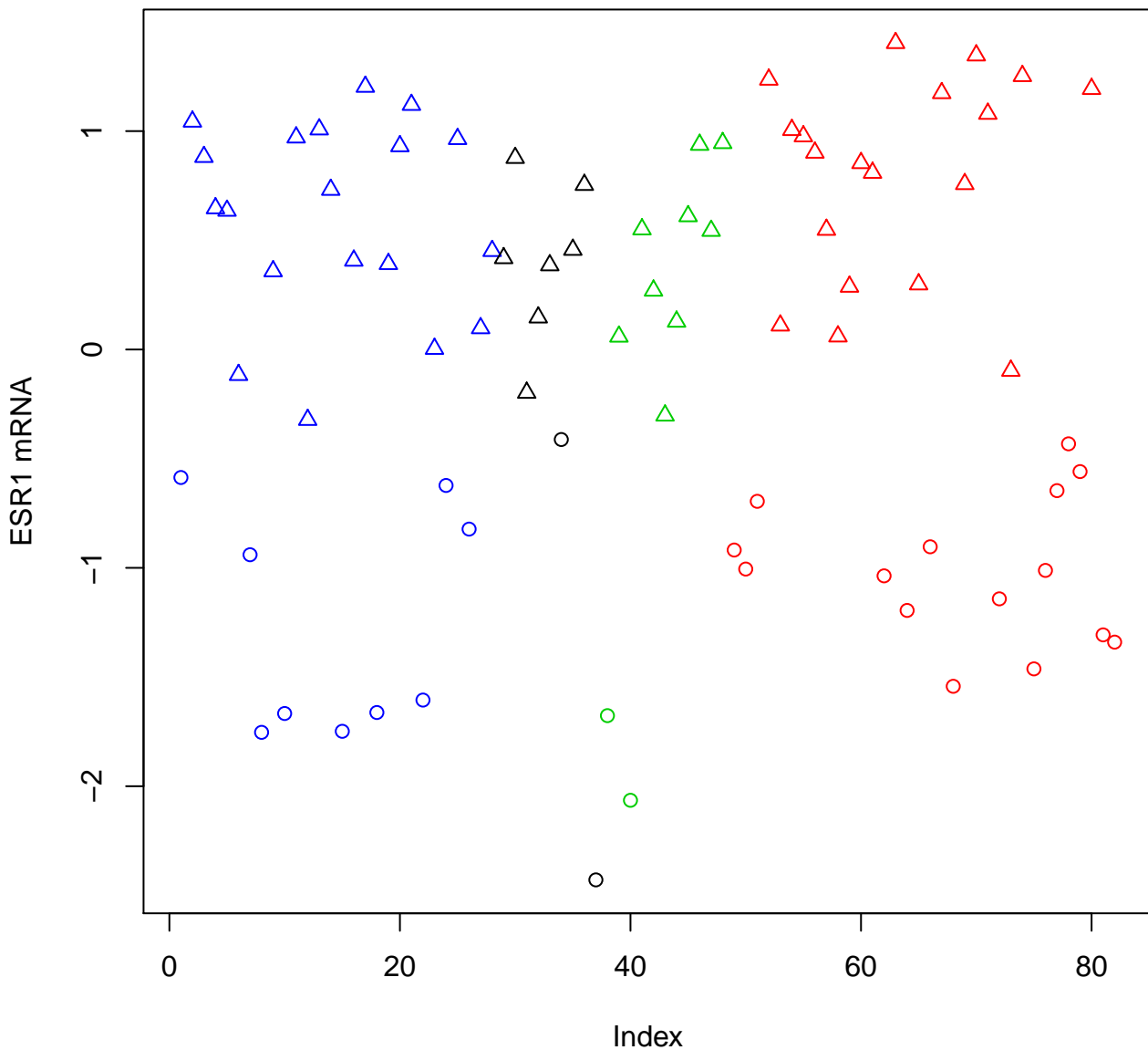

Supplement: Additional file 1: — Association of dataset with ESR1 status. The y-axis indicates the ESR1 epithelial mRNA level. Cases of invasive breast cancer are grouped along the x-axis and colored according to the dataset the sample is derived from (blue = GSE10797, black = GSE14548, green = GSE35019, red = GSE5847). The shape of each object in the plot indicates whether it was classified as ESR1-positive (triangle) or ESR1-negative (circle). This plot provides no striking evidence of batch effect effecting ESR1 status, with no significant association between the ESR1 classification (positive vs negative) and dataset site (P = 0.34). [file 13059_2015_675_MOESM1_ESM.pdf]

## Normal Epi

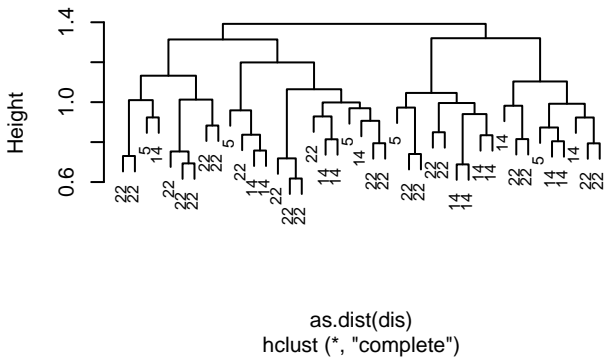

**Normal Str**

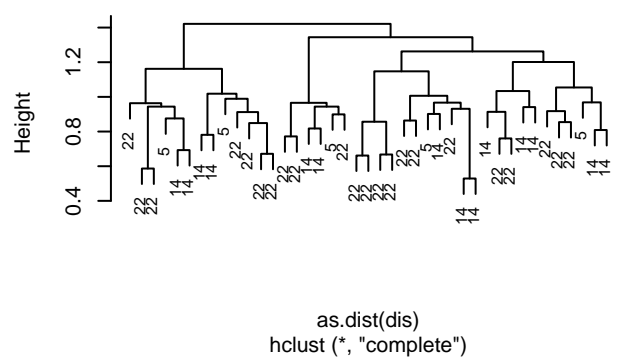

ER+ Epi

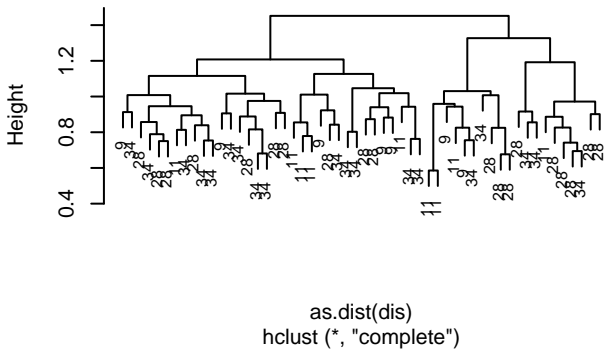

ER+ Str

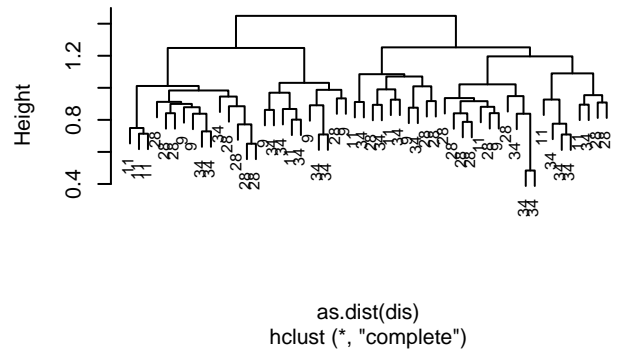

ER- Epi

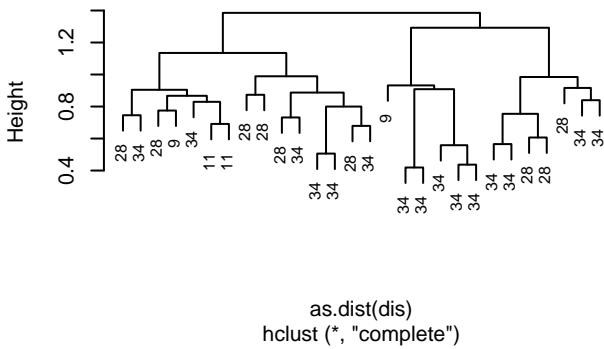

**ER- Str**

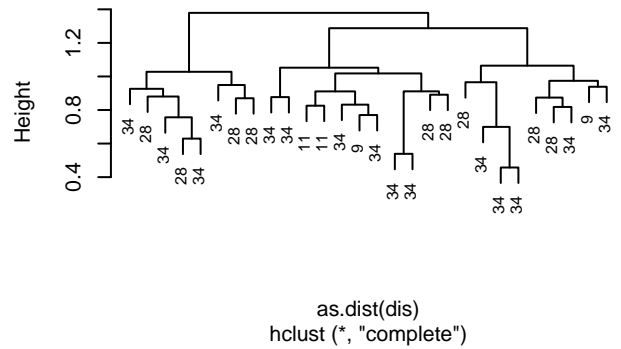

Supplement: Additional file 2: — Hierarchical clustering relationship with dataset. We performed unsupervised hierarchical clustering of the normal breast (top panel), ER-positive IBC samples (middle panel), and ER-negative IBC samples (bottom panel) from the epithelium (left panels) and the stroma (right panels). We performed clustering with Euclidean distance and complete linkage. Each leaf in the dendrogram is labeled with the dataset the sample came from (normal 5 = GSE10797, 14 = GSE14548, 22 = GSE4823; IBC 9 = GSE14548, 11 = GSE35019, 28 = GSE10797, 34 = GSE5847). [file 13059_2015_675_MOESM2_ESM.pdf]

**Normal Epi**

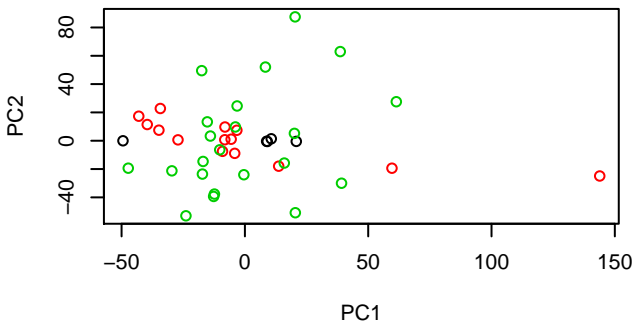

**Normal Str**

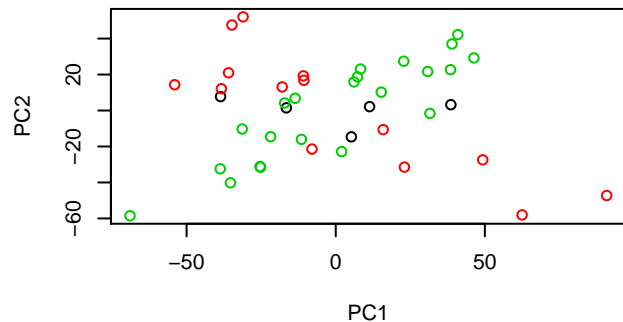

**ER+ Epi**

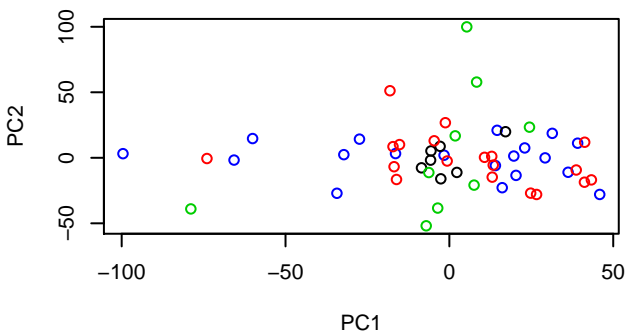

**ER+ Str**

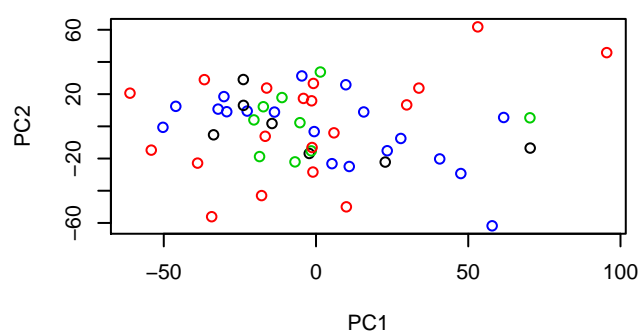

**ER- Epi**

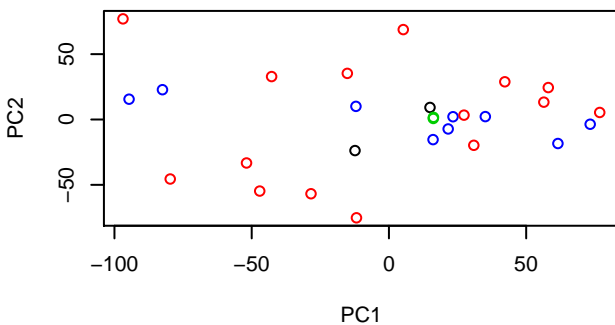

**ER- Str**

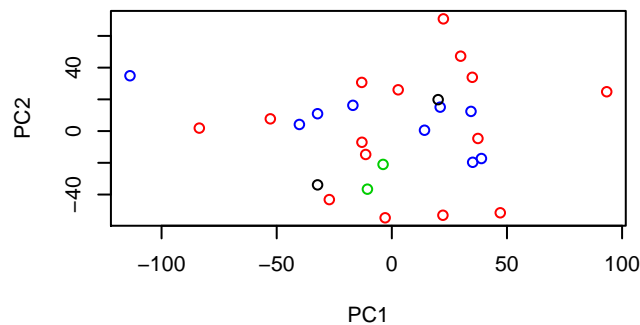

Supplement: Additional file 3: — Scatterplots of first two principal components with points colored by dataset. Each plot displays a scatterplot of a sample along the first two principal components for normal breast (top panel), ER-positive IBC samples (middle panel), and ER-negative IBC samples (bottom panel) from the epithelium (left panels) and the stroma (right panels). The color of each sample indicates the dataset it comes from (normal red = GSE14548, green = GSE4823, black = GSE10797; IBC blue = GSE10797, black = GSE14548, green = GSE35019, red = GSE5847). [file 13059_2015_675_MOESM3_ESM.pdf]

A

Normal Epi

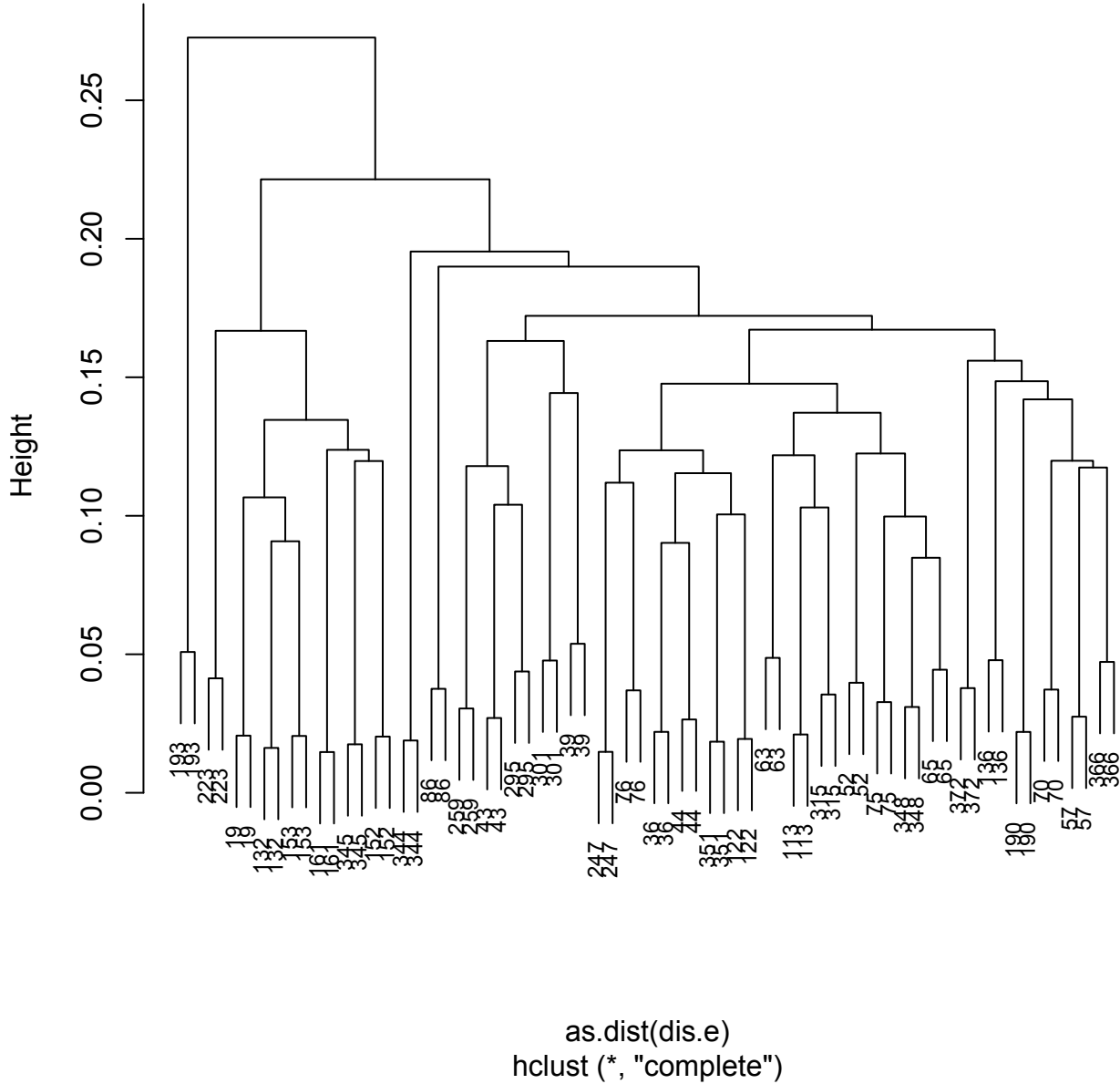

B

Normal Str

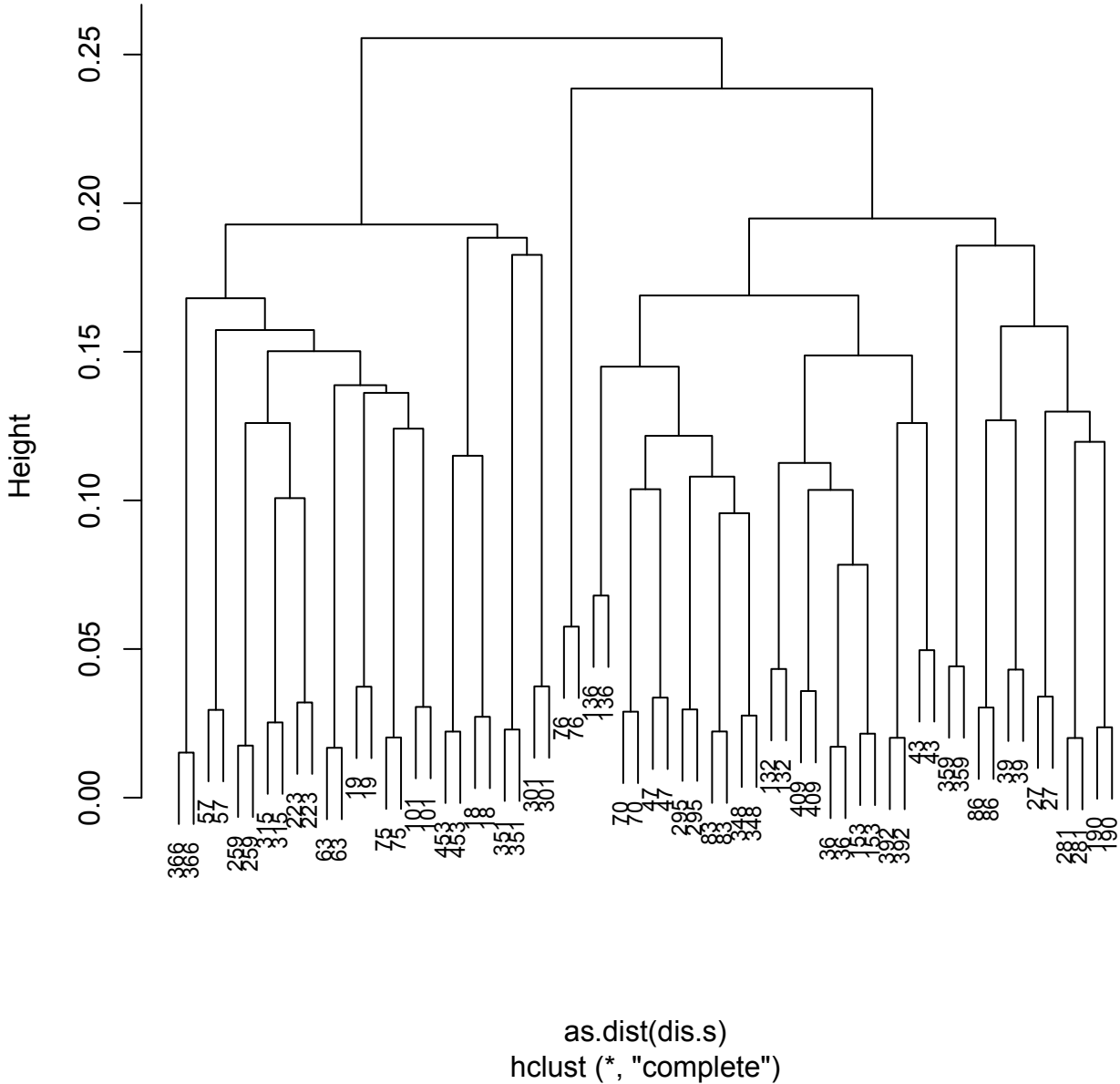

Supplement: Additional file 5: — Hierarchical clustering of largest normal breast dataset shows strong concordance between technical replicates. We performed unsupervised hierarchical clustering of the technical replicate (dye-swap) normal samples from GSE4823. There is strong concordance for each of the technical replicates, and for each sample technical replicates show the strongest correlation with each other. [file 13059_2015_675_MOESM5_ESM.pdf]

**Normal**

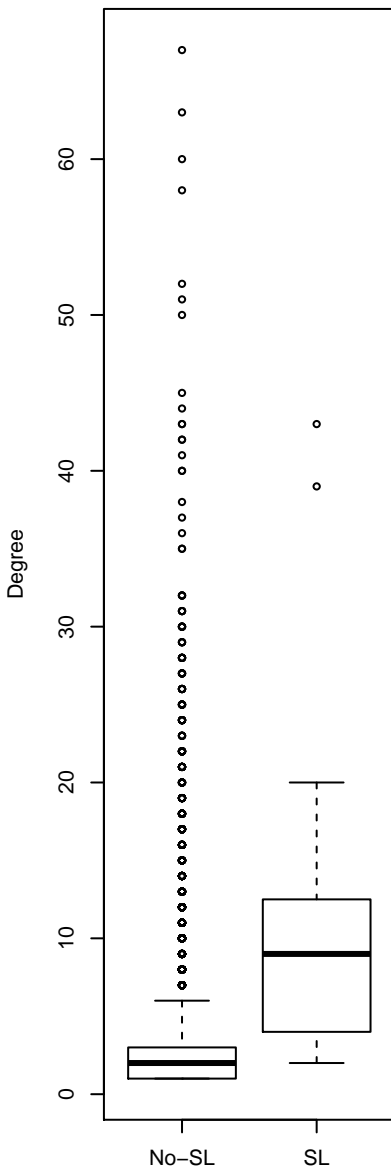

**ER-positive IBC**

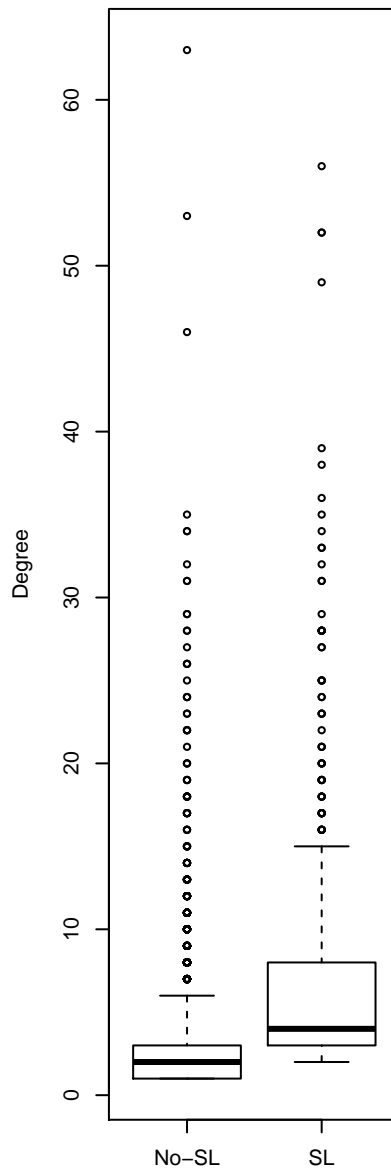

**ER-negative IBC**

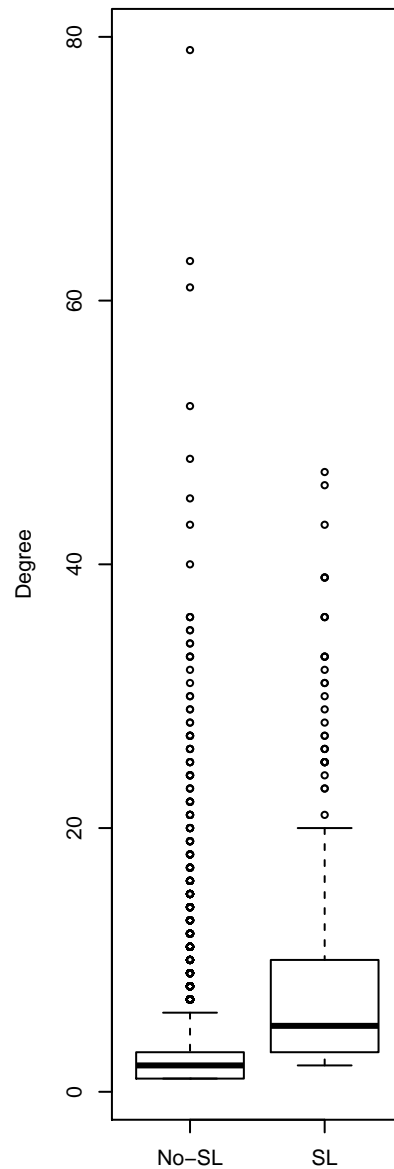

Supplement: Additional file 10: — Epithelial-stromal self-loops are significantly more connected in the epithelial-stromal networks than non-self-loops. The boxplots display the distribution of node degree for self-loop genes (SL) and non-self-loop genes (No-SL) in normal breast, ER-positive IBC, and ER-negative IBC epithelial-stromal co-expression networks. The median node degree is significantly higher for the self-loops in each of the three networks (all p < 2.2e-16). [file 13059_2015_675_MOESM10_ESM.pdf]

# Epi-Stroma Coexpression T-Statistics, Cor = 0.44

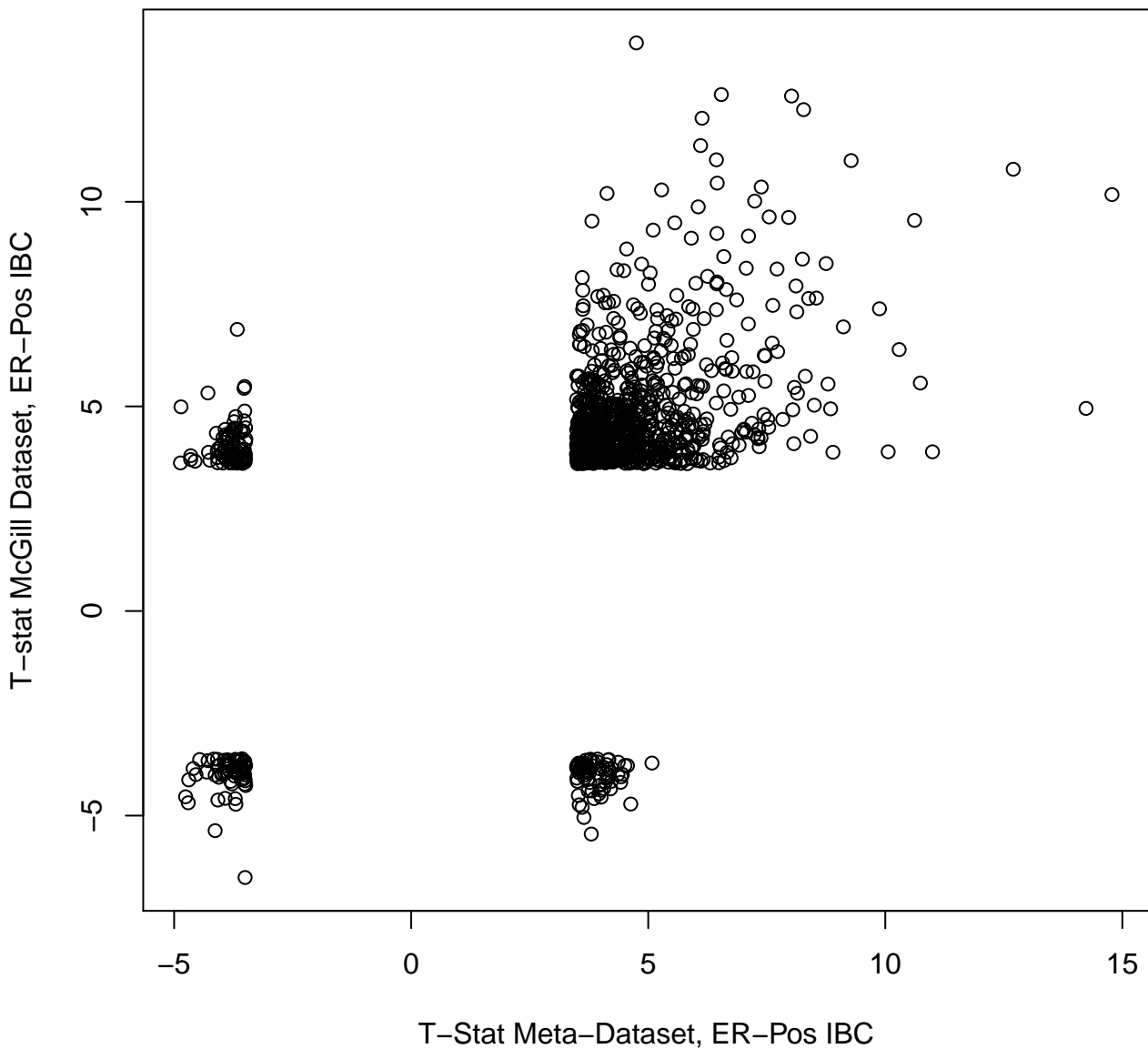

Supplement: Additional file 11: — Scatterplot of epithelial-stromal T-statistics from the McGill ER-positive IBC LCM dataset and the original meta-ER-positive IBC dataset. Each point represents an epithelial-stromal co-expression relationship, which achieved a raw p < 0.001 in the epithelial-stromal co-expression analysis. The x-axis indicates the T-statistic in the ER-positive IBC meta-dataset, and the y-axis indicates the T-statistic on the McGill ER-positive IBC dataset. The Spearman correlation is 0.44 (p < 2.2e-16). [file 13059_2015_675_MOESM11_ESM.pdf]

**Proportion of Self-Loops and Edge Strength in McGill Dataset**

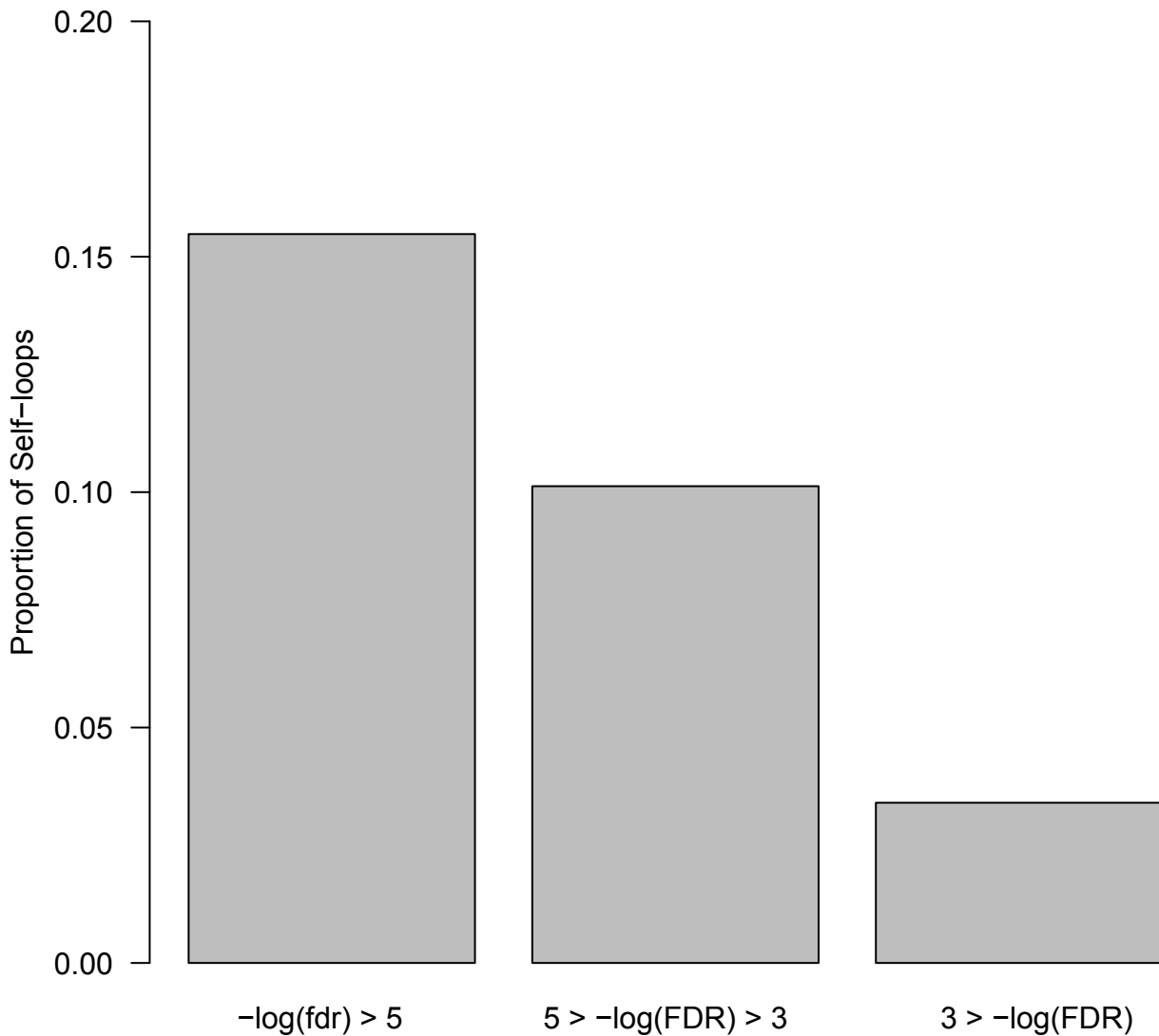

Supplement: Additional file 12: — Self-loops tend to have more significant edges in the McGill ER-positive IBC LCM dataset. The y-axis indicates the proportion of epithelial-stromal self-loops. The groups on the x-axis indicate significance windows for the epithelial-stromal interactions, ranging from most significant (−log(fdr) > 5) to the least significant (−log(fdr) < 3). [file 13059_2015_675_MOESM12_ESM.pdf]
