# Supplementary material for: Extensive rewiring of epithelial-stromal co-expression networks in breast cancer
Source: Genome Biol. 2015 Jun 19;16(1):128. doi: 10.1186/s13059-015-0675-4 (PMC4471934; doi:10.1186/s13059-015-0675-4)
Supplement: Additional file 4: — Permutation experiment to assess impact of shuffling dataset on the concordance of co-expression analyses across sites. Barplots on the left indicate the observed fraction of sign reversed correlations on the true data (Labels Not Shuffled) as compared with the median fraction of sign reversed correlations with the Labels Shuffled, when the epithelial-stromal co-expression analysis was performed separately on the two largest datasets for normal breast (top panel), ER-positive IBC (middle panel), and ER-negative IBC (lower panel). The histograms on the right show the distribution of the sign-reversed correlations across 100 iterations. The observed sign-reversed correlation fraction with the true dataset labels is indicated with a red arrow. In normal breast, there is strong evidence of batch effect, while there is no evidence of significant batch effect in ER-positive and ER-negative IBC. [file 13059_2015_675_MOESM4_ESM.pdf]

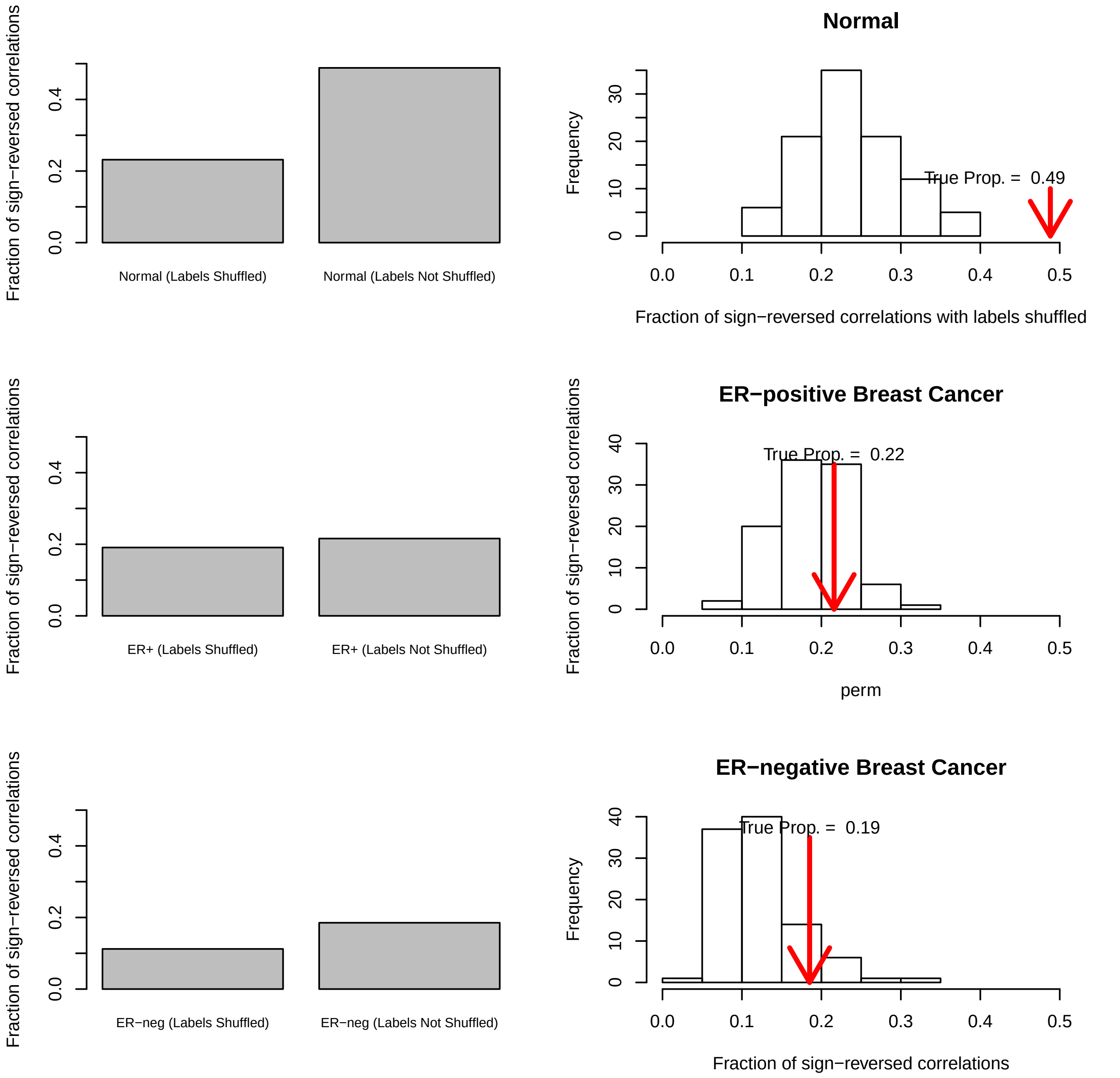
Normal
ER−positive Breast Cancer
ER−negative Breast Cancer
Fraction of sign−reversed correlations
Normal (Labels Shuffled)
Normal (Labels Not Shuffled)
ER+ (Labels Shuffled)
ER+ (Labels Not Shuffled)
ER−neg (Labels Shuffled)
ER−neg (Labels Not Shuffled)
Frequency
True Prop. = 0.49
True Prop. = 0.22
True Prop. = 0.19
Fraction of sign−reversed correlations with labels shuffled
perm
Fraction of sign−reversed correlations
