# Supplementary material for: Extensive rewiring of epithelial-stromal co-expression networks in breast cancer
Source: Genome Biol. 2015 Jun 19;16(1):128. doi: 10.1186/s13059-015-0675-4 (PMC4471934; doi:10.1186/s13059-015-0675-4)
Supplement: Additional file 9: — Proportion of genesets identified as significantly enriched in the epithelial-epithelial and epithelial-stromal co-expression networks according to geneset category. The y-axis indicates the proportion of overall genesets enriched in the normal, ER-positive IBC, and ER-negative IBC networks. The red bars indicate the epithelial-epithelial co-expression network and the blue bars indicate the epithelial-stromal co-expression network. A-C show the overall genesets, GO biological process genesets, and breast cancer prognostic signature genesets, respectively. [file 13059_2015_675_MOESM9_ESM.pdf]

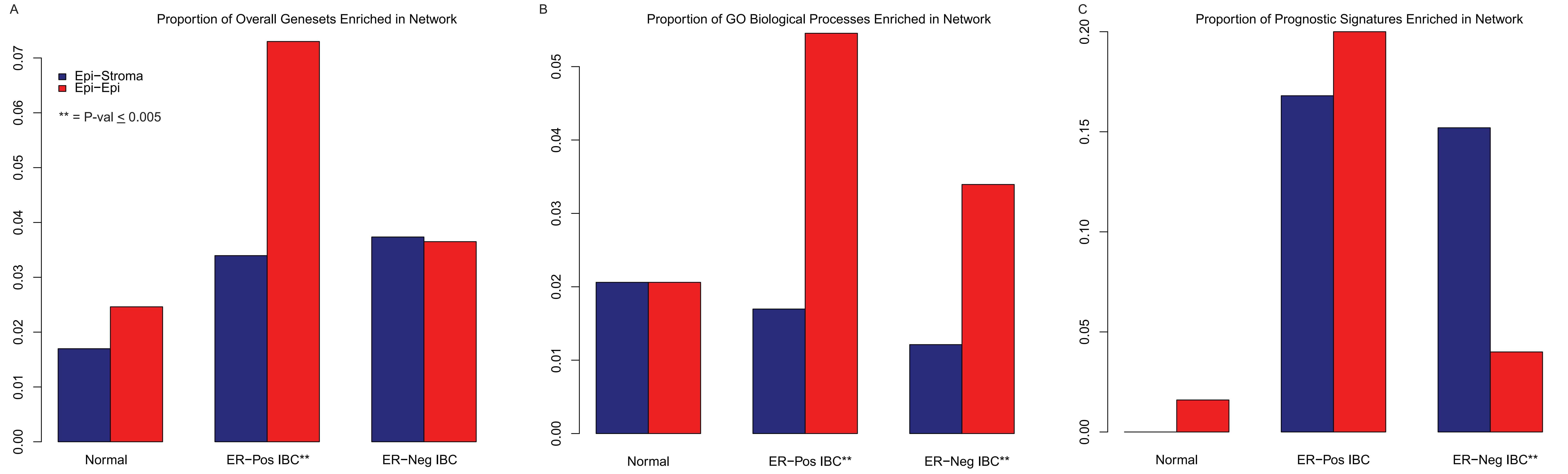

A
Proportion of Overall Genesets Enriched in Network
Epi−Stroma
Epi−Epi
** = P-val ≤ 0.005
0.00
0.01
0.02
0.03
0.04
0.05
0.06
0.07
Normal
ER−Pos IBC**
ER−Neg IBC
B
Proportion of GO Biological Processes Enriched in Network
0.00
0.01
0.02
0.03
0.04
0.05
Normal
ER−Pos IBC**
ER−Neg IBC**
C
Proportion of Prognostic Signatures Enriched in Network
0.00
0.05
0.10
0.15
0.20
Normal
ER−Pos IBC
ER−Neg IBC**
