# Supplementary material for: Extensive rewiring of epithelial-stromal co-expression networks in breast cancer
Source: Genome Biol. 2015 Jun 19;16(1):128. doi: 10.1186/s13059-015-0675-4 (PMC4471934; doi:10.1186/s13059-015-0675-4)
Supplement: Additional file 17: — The expression data for each sample from GEO used in our analyses. This zip directory contains ten text files, each labeled with the dataset’s GEO series identifier, an indicator of whether the data is from normal (No) or breast cancer (Br), an indicator of whether the data is from the epithelium (Epi) or stroma (Str), and the number of samples in the dataset. [file 13059_2015_675_MOESM17_ESM.docx]

**Additional file 17.** The expression data for each sample from GEO used in our analyses is provided in the zip directory (Additional file 17. ExpressionData.zip). This zip directory contains ten text files, each labeled with the dataset’s GEO series identifier, an indicator of whether the data is from normal (No) or breast cancer (Br), an indicator of whether the data is from the epithelium (Epi) or stroma (Str), and the number of samples in the dataset. The zip directory can be downloaded from:

https://raw.githubusercontent.com/becklab/esnet/master/Additional%20files/Additional%20file%2017.%20ExpressionData.zip
