# Supplementary material for: Extensive rewiring of epithelial-stromal co-expression networks in breast cancer
Source: Genome Biol. 2015 Jun 19;16(1):128. doi: 10.1186/s13059-015-0675-4 (PMC4471934; doi:10.1186/s13059-015-0675-4)
Supplement: Additional file 18: — Epithelial-stromal co-expression. This zip directory contains three files: Normal_22_ES.txt, ER_Positive_ES.txt, and ER_Negative_ES.txt. Each file is a tab-delimited table containing the epithelial-stromal interactions that achieved a raw p-value of 1e-3 in the epithelial-stromal co-expression network indicated by the file’s name (Normal, ER-positive IBC, ER-negative IBC). Each row indicates an epithelial-stromal interaction. The first column indicates the gene expressed in the stroma, the second column indicates the gene expressed in the epithelium, the third column indicates the interaction’s T-statistic, the fourth column indicates the raw p-value associated with the T-statistic, and the fifth column indicates the interaction’s FDR. [file 13059_2015_675_MOESM18_ESM.docx]

**Additional file 18**. Epithelial-stromal coexpression results (Additional file 18. EpiStromaCoExpressResults.zip). This zip directory contains three files (Normal_22_ES.txt, ER_Positive_ES.txt, ER_Negative_ES.txt). Each file is a tab-delimited table containing the epithelial-stromal interactions that achieved a raw p-value of 1e-3 in the epithelial-stromal coexpression network indicated by the file’s name (Normal, ER-positive IBC, ER-Negative IBC). Each row indicates an epithelial-stromal interaction. The first column indicates the gene expressed in the stroma, the second column indicates the gene expressed in the epithelium, the third column indicates the interaction’s T-statistic, the fourth column indicates the raw p-value associated with the T-statistic, and the fifth column indicates the interaction’s FDR.

The zip directory can be downloaded from:

https://raw.githubusercontent.com/becklab/esnet/master/Additional%20files/Additional%20file%2018.%20EpiStromaCoExpressResults.zip
