# Supplementary material for: Extensive rewiring of epithelial-stromal co-expression networks in breast cancer
Source: Genome Biol. 2015 Jun 19;16(1):128. doi: 10.1186/s13059-015-0675-4 (PMC4471934; doi:10.1186/s13059-015-0675-4)
Supplement: Additional file 20: — Knitr script for performing statistical analyses in R (analysis.knit.zip). This zip directory contains two files, analysis.rnw and analysis.pdf, for running the main statistical analyses from the paper in R (analysis.rnw) and for producing a file containing both the code and the results and figures from the analyses (analysis.pdf). [file 13059_2015_675_MOESM20_ESM.zip › analysis.pdf]

Extensive Rewiring of Epithelial-Stromal Coexpession Networks in Breast Cancer

April 10, 2015

Eun-Yeong Oh, Stephen M Christensen, Sindhu Ghanta, Jong Cheol Jeong, Octavian Bucur, Benjamin Glass, Laleh Montaser-Kouhsari, Nicholas W Knoblauch, Nicholas Bertos,Sadiq M. I. Saleh, Benjamin Haibe-Kains, Morag Park,Andrew H Beck [abeck2@bidmc.harvard.edu]

1 Set-Up

```
rm(list = ls())
options(width = 60)
list.of.packages <- c("MatrixEQTL", "RcppArmadillo", "sqldf", "plyr", "mclust",
    "reshape2", "RCurl", "igraph", "RedeR", "SANTA", "GSA", "Vennerable")
new.packages <- list.of.packages[!(list.of.packages %in% installed.packages()[,
    "Package"])]
if (length(new.packages)) install.packages(new.packages)
require("MatrixEQTL")

## Loading required package:  MatrixEQTL

require("RcppArmadillo")

## Loading required package:  RcppArmadillo

require("sqldf")

## Loading required package:  sqldf
## Loading required package:  gsubfn
## Loading required package:  proto
## Loading required package:  RSQLite
## Loading required package:  DBI

require("plyr")

## Loading required package:  plyr

require("mclust")

## Loading required package:  mclust
## Package 'mclust' version 4.3

require("reshape2")

## Loading required package:  reshape2

require("RCurl")

## Loading required package:  RCurl
## Loading required package:  bitops

require("igraph")

## Loading required package:  igraph

require("RedeR")
```

```
## Loading required package:  RedeR
## ***This is RedeR 1.12.9!  For a quick start, please type 'vignette('RedeR')'.
##    Supporting information is available at Genome Biology 13:R29,
2012,
##    (doi:10.1186/gb-2012-13-4-r29).
```

```r
require("SANTA")
```

```
## Loading required package:  SANTA
```

```r
require("GSA")
```

```
## Loading required package:  GSA
```

```r
require("Vennerable")
```

```
## Loading required package:  Vennerable
## Loading required package:  graph
##
## Attaching package:  'graph'
##
## The following object is masked from 'package:RedeR':
##
##    updateGraph
##
## The following objects are masked from 'package:igraph':
##
##    degree, edges
##
## The following object is masked from 'package:plyr':
##
##    join
##
## Loading required package:  RBGL
##
## Attaching package:  'RBGL'
##
## The following object is masked from 'package:igraph':
##
##    transitivity
##
## Loading required package:  grid
## Loading required package:  lattice
## Loading required package:  RColorBrewer
## Loading required package:  reshape
##
## Attaching package:  'reshape'
##
```

```
## The following objects are masked from 'package:reshape2':
##
##     colsplit, melt, recast
##
## The following objects are masked from 'package:plyr':
##
##     rename, round_any
##
## Loading required package:  gtools
## Loading required package:  xtable

run.eqtl <- function(x, name) {
    res = Matrix_eQTL_main(snps = SlicedData$new(x$Str), gene = SlicedData$new(x$Epi),
        cvrt = SlicedData$new(), output_file_name = paste0(name, ".txt"), useModel = modelLI
        verbose = T, output_file_name.cis = 0, pvOutputThreshold = 0.001)
    output <- read.table(paste0(name, ".txt"), header = T, sep = "\t")
    output <- output[, -c(3)]
    colnames(output) <- c("Stroma", "Epi", "t.stat", "p.value", "FDR")
    write.table(output, file = paste0(name, ".txt"), sep = "\t", row.names = F,
        quote = F)
}
```

2 Select Most Variant Probe and Scale Data

```
## Beginning With GEO Data Files
## Data has common gene symbols across platform from within a cancer type

files=c("GSE10797_BrEpi_28_anno_common.txt","GSE10797_BrStr_28_anno_common.txt","GSE14548_Br
dataDir= 'https://raw.githubusercontent.com/becklab/esnet/master/'
files.dir=paste(dataDir,files,sep="/")


## We will summarize probes, by taking the probe with the most variance
## Then we will scale and save the data
for(i in 1:length(files)){
 my_data <- getURL(files.dir[i],ssl.verifypeer=FALSE)
 test <- read.csv(textConnection(my_data), sep='\t',head=T)
 ntest <- test[,-(1)]
 dfl <- split(ntest,test$Gene.Symbol)

 var.probe <- sapply(dfl,function(x)apply(x,1,function(y)var(y)),simplify=F)

 max.probes <- sapply(var.probe,which.max)
```

```r
 ndfl <- list()
 for(j in 1:length(max.probes)){
   ndfl[[j]]<-dfl[[j]][max.probes[j],]
 }
 nndfl <- do.call(what="rbind",ndfl)
 nndfl <- t(scale(t(nndfl)))
 nndfl <- data.frame(gene=names(dfl),nndfl)
 write.table(nndfl,file=paste0(files[i],"_SCALED.txt"),col.names=T,row.names=F,quote=F,sep="
 cat("File ",i," of ",length(files),"\n")
}
```

```
## File  1  of  10
## File  2  of  10
## File  3  of  10
## File  4  of  10
## File  5  of  10
## File  6  of  10
## File  7  of  10
## File  8  of  10
## File  9  of  10
## File  10  of  10
```

3 Combine Data Sets

```r
epi.stroma.files <- c("GSE10797_BrEpi_28_anno_common.txt_SCALED.txt","GSE10797_BrStr_28_anno

all.epi.stroma.mats <- lapply(epi.stroma.files,FUN=read.table,header=T,sep="\t",check.names=
good.rows <- Reduce(intersect,x=lapply(all.epi.stroma.mats,FUN=rownames))
all.epi.stroma.mats <- lapply(all.epi.stroma.mats,'[',good.rows,)
grps <- gsub(".+GSE[0-9]+_([A-Za-z]+)_.+","\\1",epi.stroma.files)
epi.stroma.num <- gsub(".+GSE[0-9]+_.+_([0-9]+)_.+","\\1",epi.stroma.files)
egrps <- gsub("(.+)[ES].*","\\1",grps)
epi.str <- gsub(".+([ES]..).*","\\1",grps)
Epis <- epi.stroma.files[epi.str=="Epi"]
Strs <- epi.stroma.files[epi.str=="Str"]
Epi.grps <- paste0(egrps,epi.stroma.num)[epi.str=="Epi"]
negrps <- egrps[epi.str=="Epi"]
nepi.stroma.num <- epi.stroma.num[epi.str=="Epi"]
newcols <- unlist(mapply(function(x,y,z)paste(x,y,z,sep="_"),lapply(all.epi.stroma.mats,coln
bigmat <- do.call("cbind",all.epi.stroma.mats)
colnames(bigmat)<- newcols
new.egrps <- gsub(".+((No)|(Br)|(Dcis)).+","\\1",newcols)
allmat.by.ctype <- lapply(split(data.frame(t(bigmat)),new.egrps),t)
```

```r
allmat.by.ctype.by.ES <- lapply(allmat.by.ctype,function(x){
 EpiStr <- gsub(".+((Epi)|(Str)).+","\\1",colnames(x))
 tsplit <- split(data.frame(t(x)),EpiStr)
 return(lapply(tsplit,t))
 })

t.ER <- allmat.by.ctype.by.ES$Br
ERclass <- Mclust(t.ER$Epi["ESR1",],G=2)
boxplot(t.ER$Epi["ESR1",]~ERclass$class,names=c("ER-neg","ER-pos"),ylab="ESR1 mRNA")
```

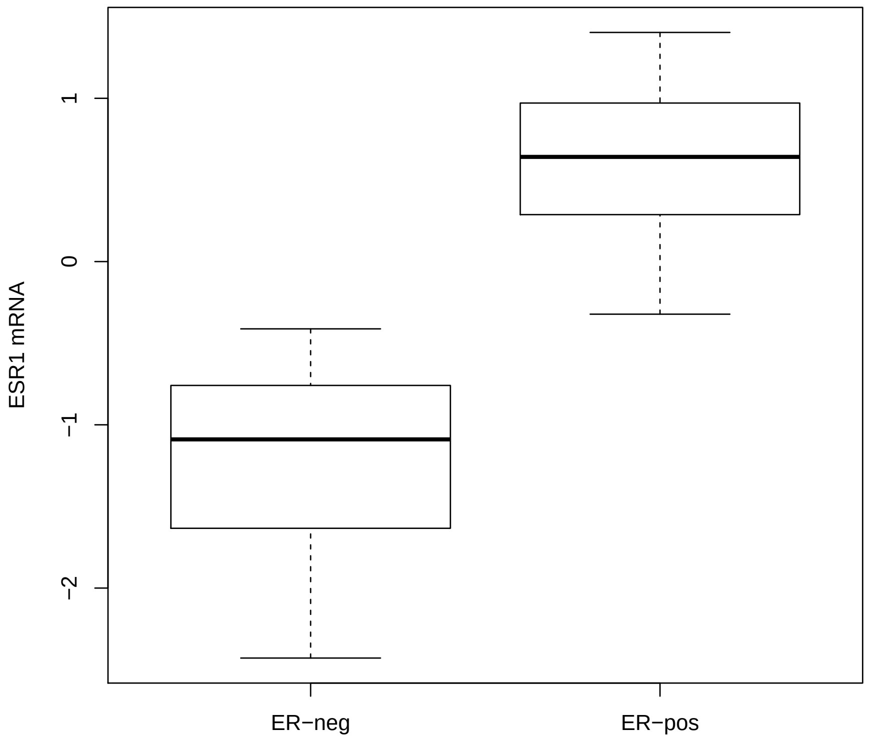


```r
# Check ER correlation with SITE
sites=unlist(lapply(strsplit(colnames(t.ER$Epi),"_"),function(xx)(xx[4])))
t1=table(ERclass$class,sites)
t1

##    sites
```

```
##      11 28 34  9
##    1  2  9 15  2
##    2  9 19 19  7

chisq.test(t1) # p =0.34 , No significant association of site with ER status

## Warning in chisq.test(t1):  Chi-squared approximation may be incorrect

##
##  Pearson's Chi-squared test
##
## data:  t1
## X-squared = 3.3692, df = 3, p-value = 0.3381

plot(t.ER$Epi["ESR1",],col=sites,pch=ERclass$class,main="No Significant Association of Datas
```

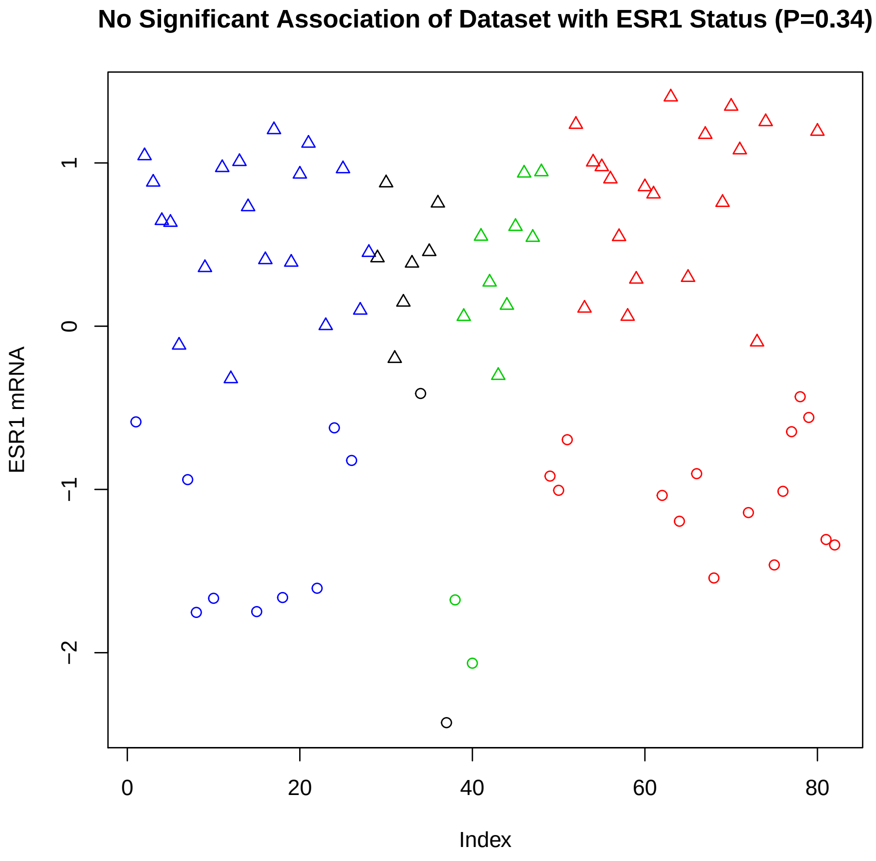

```
BrEpi.posneg <- lapply(split(data.frame(t(t.ER$Epi)),ERclass$classification),t)
BrStr.posneg <- lapply(split(data.frame(t(t.ER$Str)),ERclass$classification),t)
allmat.by.ctype.by.ES$BrP <- list(Epi=BrEpi.posneg[["2"]],Str=BrStr.posneg[["2"]])
allmat.by.ctype.by.ES$BrN <- list(Epi=BrEpi.posneg[["1"]],Str=BrStr.posneg[["1"]])
allmat.by.ctype.by.ES$Br <- NULL
```

4 Run Matrix EQTL on Merged Datasts for IBC and Single Dataset for Normal

```
run.eqtl(allmat.by.ctype.by.ES$BrP,"ER_Positive_ES")

## Processing covariates
## Task finished in  0  seconds
## Processing gene expression data (imputation, residualization, etc.)
## Task finished in  0.03  seconds
## Creating output file(s)
## Task finished in  0.02  seconds
## Performing eQTL analysis
## 100.00% done, 330,368 eQTLs
## Task finished in  14.55  seconds
##

run.eqtl(allmat.by.ctype.by.ES$BrN,"ER_Negative_ES")

## Processing covariates
## Task finished in  0  seconds
## Processing gene expression data (imputation, residualization, etc.)
## Task finished in  0.03  seconds
## Creating output file(s)
## Task finished in  0.02  seconds
## Performing eQTL analysis
## 100.00% done, 548,641 eQTLs
## Task finished in  19.11  seconds
##

run.eqtl(allmat.by.ctype.by.ES$No,"Normal_22_ES")

## Processing covariates
## Task finished in  0  seconds
## Processing gene expression data (imputation, residualization, etc.)
## Task finished in  0.03  seconds
## Creating output file(s)
## Task finished in  0  seconds
```

```
## Performing eQTL analysis
## 100.00% done, 243,474 eQTLs
## Task finished in  10.53  seconds
##
```

5 Computing Descriptive Stats on Networks

```
# Table 1
# Most significant normal breast epi-stroma interactions
head(norm)

##   Stroma     Epi   t.stat      p.value          FDR
## 1 SPINK1  IPCEF1 29.29150 6.697883e-18 9.168732e-10
## 2  PNMA2 HSPA12A 21.14947 3.706472e-15 1.740958e-07
## 3  PNMA2   ALDOB 21.10843 3.847158e-15 1.740958e-07
## 4  PNMA2 SULT1E1 20.80299 5.087175e-15 1.740958e-07
## 5 SPINK1     DPT 19.97923 1.101624e-14 2.900225e-07
## 6  PNMA2   SFTPB 19.82989 1.271192e-14 2.900225e-07

# Most significant ER-positive IBC epi-stroma interactions
head(BrP)

##    Stroma     Epi   t.stat      p.value          FDR
## 1 CEACAM5 CEACAM5 18.04539 4.840840e-24 6.626626e-16
## 2  S100A7  S100A7 14.78167 2.925105e-20 2.002088e-12
## 3   FAM5C   FAM5C 14.23503 1.417667e-19 6.468815e-12
## 4    BEX1    BEX1 12.69880 1.460347e-17 4.997673e-10
## 5   IFIH1   IFIH1 10.99527 3.546667e-15 9.710065e-08
## 6     AGT     AGT 10.74010 8.335618e-15 1.901771e-07

# Most significant ER-negative IBC epi-stroma interactions
head(BrN)

##   Stroma   Epi   t.stat      p.value          FDR
## 1   ORM1  ORM1 19.28556 6.324143e-17 8.657119e-09
## 2   PCP4  PCP4 13.94548 1.400947e-13 9.588778e-06
## 3  MMP10 MMP10 13.64965 2.293304e-13 1.046435e-05
## 4   DSC3  DSC3 13.39460 3.530769e-13 1.208318e-05
## 5   CPB1 NPY5R 12.45865 1.817112e-12 4.974890e-05
## 6  IMPA2 IMPA2 12.10477 3.456164e-12 7.885239e-05
```

```r
# Table 2
# Most highly connected nodes in normal breast
head(Genecomp[order(Genecomp[,"NormDegree"],decreasing=T),])

##        NormDegree StromaNorm EpiNorm BrPDegree StromaBrP
## GABRA6         67         56      11         1         1
## FGF22          63         63       0         2         0
## POU3F1         60         54       6         0         0
## FPR3           58         58       0         2         0
## RPE65          52         20      32         1         1
## ASPM           51         51       0        31        18
##        EpiBrP BrNDegree StromaBrN EpiBrN norm.self erp.self
## GABRA6      0         4         2      2         0        0
## FGF22       2         0         0      0         0        0
## POU3F1      0        15        10      5         0        0
## FPR3        2         2         0      2         0        0
## RPE65       0         7         5      2         0        0
## ASPM       13        15         3     12         0        1
##        ern.self
## GABRA6        0
## FGF22         0
## POU3F1        0
## FPR3          0
## RPE65         0
## ASPM          0

# Most highly connected nodes in ER-positive IBC
head(Genecomp[order(Genecomp[,"BrPDegree"],decreasing=T),])

##        NormDegree StromaNorm EpiNorm BrPDegree StromaBrP
## BDNF            2          1       1        63        61
## IFIH1           0          0       0        56        37
## FUT5            0          0       0        53        35
## KIF20A          1          1       0        52        29
## UBE2C           0          0       0        52        26
## FOXM1           0          0       0        49        33
##        EpiBrP BrNDegree StromaBrN EpiBrN norm.self erp.self
## BDNF        2         2         0      2         0        0
## IFIH1      19         9         3      6         0        1
## FUT5       18         0         0      0         0        0
## KIF20A     23        43         6     37         0        1
## UBE2C      26        16        14      2         0        1
## FOXM1      16        15         7      8         0        1
##        ern.self
## BDNF          0
## IFIH1         0
```

```
## FUT5           0
## KIF20A         0
## UBE2C          0
## FOXM1          0

# Most highly connected nodes in ER-negative IBC
head(Genecomp[order(Genecomp[,"BrNDegree"],decreasing=T),])

##           NormDegree StromaNorm EpiNorm BrPDegree StromaBrP
## NTS                2          2       0         4         0
## C11orf9            4          0       4         7         6
## SRPK1              0          0       0         1         0
## DENND5B            0          0       0         0         0
## BUB1               1          1       0        17         1
## EZH2               1          0       1         2         1
##           EpiBrP BrNDegree StromaBrN EpiBrN norm.self
## NTS            4        79        79      0         0
## C11orf9        1        63        63      0         0
## SRPK1          1        61        61      0         0
## DENND5B        0        52        46      6         0
## BUB1          16        48         0     48         0
## EZH2           1        47        45      2         0
##           erp.self ern.self
## NTS              0        0
## C11orf9          0        0
## SRPK1            0        0
## DENND5B          0        0
## BUB1             0        0
## EZH2             0        1
```

7 Figure 2 – Network Barplots,eval = TRUE

```
# A. Number of Significant Epithelial-Stromal Co-expression Interactions
barplot(c(sum(norm[,"FDR"]<0.05),sum(BrP[,"FDR"]<0.05),sum(BrN[,"FDR"]<0.05)),names=c("Norma
```

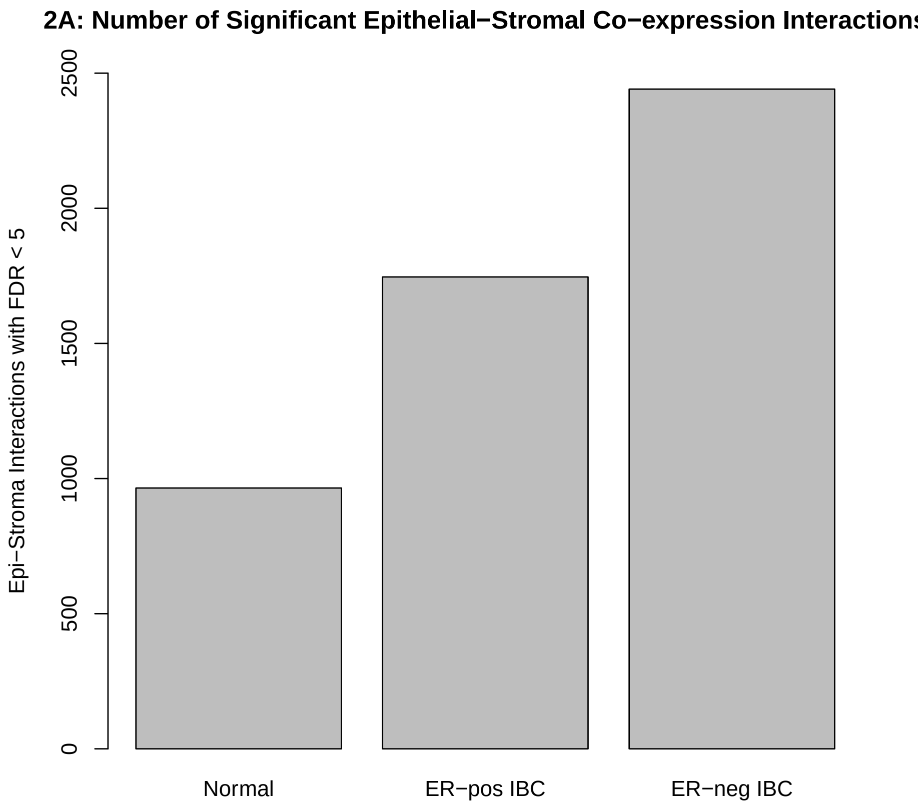


```
norm1.sl=apply(norm[norm[,"FDR"]<0.05,],1,function(x)(1*(x[1]==x[2])))
tno=table(norm1.sl)
tno[2]/sum(tno) * 100

##         1
## 0.5181347

brp1.sl=apply(BrP[BrP[,"FDR"]<0.05,],1,function(x)(1*(x[1]==x[2])))
tp=table(brp1.sl)
tp[2]/sum(tp) * 100

##        1
## 19.01489

brn1.sl=apply(BrN[BrN[,"FDR"]<0.05,],1,function(x)(1*(x[1]==x[2])))
tn=table(brn1.sl)
tn[2]/sum(tn) * 100
```

```
##        1
## 8.603032

# B. Proportion of Self-Loops Among Significant Interactions
barplot(c(tno[2]/sum(tno), tn[2]/sum(tn),tp[2]/sum(tp)),beside=T,names=c("Normal","ER-neg IB
```

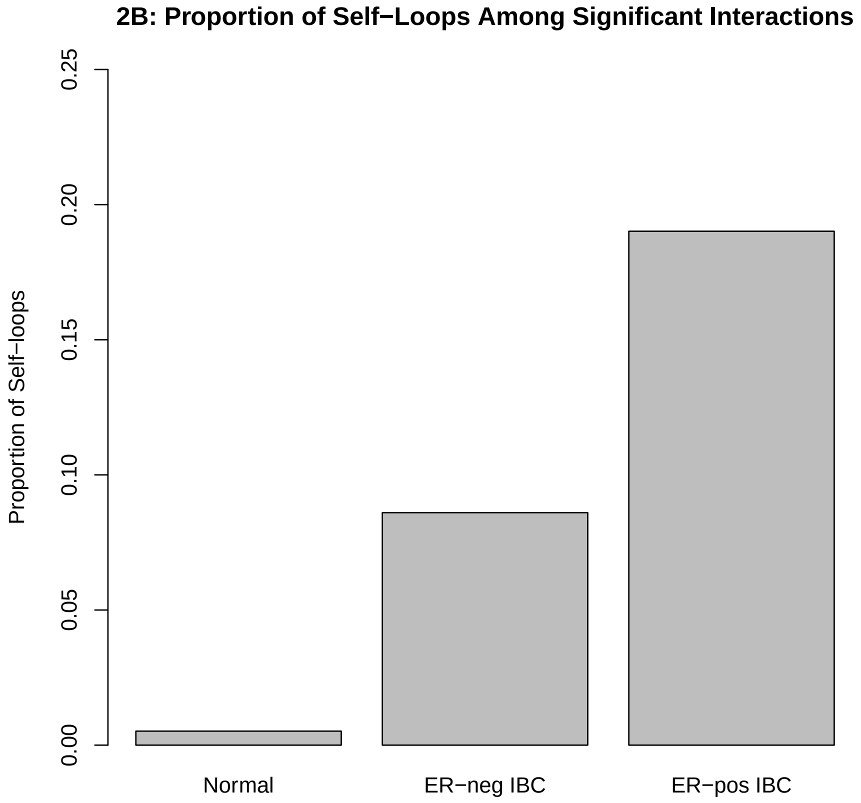


```
# C. Proportion of Self Loops and Coexpression Interaction Significance in ER positive IBC
brp1.sl=apply(BrP,1,function(x)(1*(x[1]==x[2])))
fdr=BrP[,"FDR"]
p1=sum(brp1.sl[fdr<1e-4])/sum(fdr<1e-4)
p2=sum(brp1.sl[fdr>1e-4 & fdr<1e-3])/sum(fdr>1e-4 & fdr<1e-3)
p3=sum(brp1.sl[fdr>1e-3 & fdr<1e-2])/sum(fdr>1e-3 & fdr<1e-2)
p4=sum(brp1.sl[fdr>1e-2 & fdr<1e-1])/sum(fdr>1e-2 & fdr<1e-1)
p5=sum(brp1.sl[fdr>1e-1])/sum(fdr>1e-1)
barplot(c(p1,p2,p3,p4,p5),main="2C: Proportion Self-Loops in ER-positive IBC",names=c("-log(
```

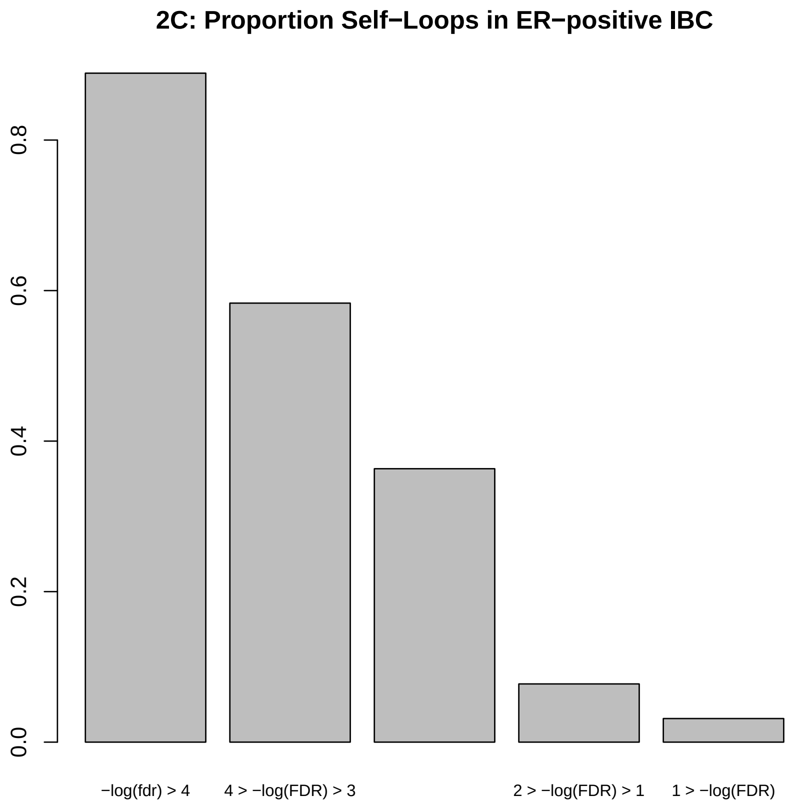


```
# D. Proportion of Self Loops and Coexpression Interaction Significance in ER negative IBC
brn1.sl=apply(BrN,1,function(x)(1*(x[1]==x[2])))
fdr=BrN[,"FDR"]
p1=sum(brn1.sl[fdr<1e-4])/sum(fdr<1e-4)
p2=sum(brn1.sl[fdr>1e-4 & fdr<1e-3])/sum(fdr>1e-4 & fdr<1e-3)
p3=sum(brn1.sl[fdr>1e-3 & fdr<1e-2])/sum(fdr>1e-3 & fdr<1e-2)
p4=sum(brn1.sl[fdr>1e-2 & fdr<1e-1])/sum(fdr>1e-2 & fdr<1e-1)
p5=sum(brn1.sl[fdr>1e-1])/sum(fdr>1e-1)
barplot(c(p1,p2,p3,p4,p5),main="2D: Proportion Self-Loops in ER-negative IBC", ,names=c("-lo
```

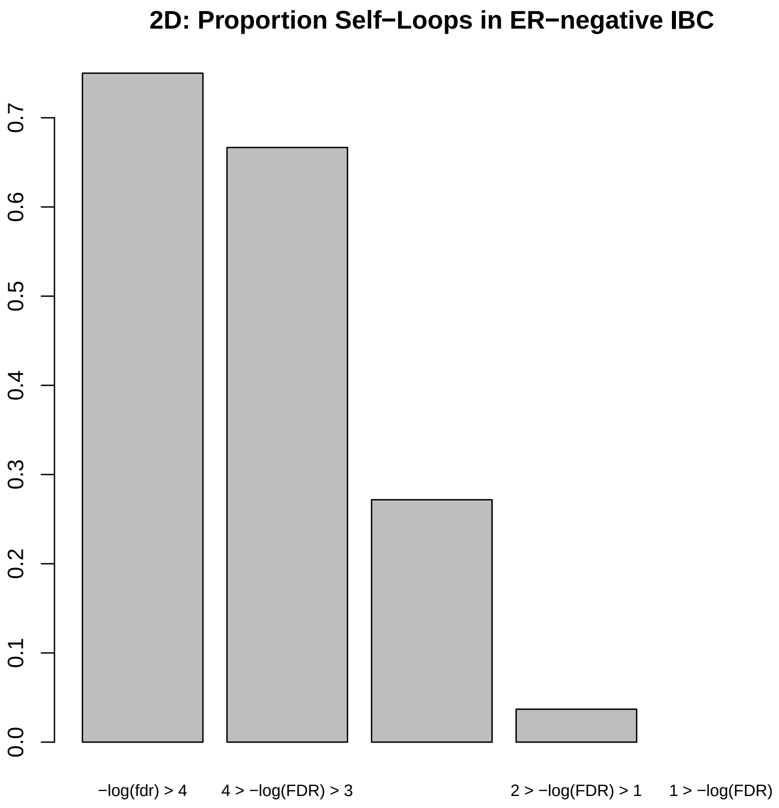

8 RedeR Visualization, Figure 3

```
rdp <- RedPort()
calld(rdp)

## RedeR is ready!

nodeA=Genecomp

ng=graph.data.frame(norm)
brpg=graph.data.frame(BrP)
brng=graph.data.frame(BrN)

ng <- set.edge.attribute(ng,"weight",value=1/abs(E(ng)$t.stat))
brpg <- set.edge.attribute(brpg,"weight",value=1/abs(E(brpg)$t.stat))
```

```
brng <- set.edge.attribute(brng,"weight",value=1/abs(E(brng)$t.stat))

sl=rownames(nodeA)[nodeA[,"norm.self"]==1]
ng <- set.vertex.attribute(ng,"degree",value=igraph::degree(ng))
ng <- set.vertex.attribute(ng,"selfloop",value=1*(is.element(V(ng)$name,sl)))
ng1=subgraph(ng,V(ng)$degree>5)

## Warning in .Call("R_igraph_subgraph", graph, as.igraph.vs(graph,
v) - 1, :  At structural_properties.c:1945 :igraph_subgraph is deprecated
from igraph 0.6, use igraph_induced_subgraph instead

sl=rownames(nodeA)[nodeA[,"erp.self"]==1]
brpg <- set.vertex.attribute(brpg,"degree",value=igraph::degree(brpg))
brpg <- set.vertex.attribute(brpg,"selfloop",value=1*(is.element(V(brpg)$name,sl)))
brpg1=subgraph(brpg,V(brpg)$degree>5)

## Warning in .Call("R_igraph_subgraph", graph, as.igraph.vs(graph,
v) - 1, :  At structural_properties.c:1945 :igraph_subgraph is deprecated
from igraph 0.6, use igraph_induced_subgraph instead

brpg1

## IGRAPH DNW- 754 2331 --
## + attr: name (v/c), degree (v/n), selfloop (v/n),
##   t.stat (e/n), p.value (e/n), FDR (e/n), weight
##   (e/n)

sl=rownames(nodeA)[nodeA[,"ern.self"]==1]
brng <- set.vertex.attribute(brng,"degree",value=igraph::degree(brng))
brng <- set.vertex.attribute(brng,"selfloop",value=1*(is.element(V(brng)$name,sl)))
brng1=subgraph(brng,V(brng)$degree>5)

## Warning in .Call("R_igraph_subgraph", graph, as.igraph.vs(graph,
v) - 1, :  At structural_properties.c:1945 :igraph_subgraph is deprecated
from igraph 0.6, use igraph_induced_subgraph instead

sum(V(ng1)$selfloop==1)/length(V(ng1)$selfloop)

## [1] 0.01822323

sum(V(brpg1)$selfloop==1)/length(V(brpg1)$selfloop)

## [1] 0.3143236

sum(V(brng1)$selfloop==1)/length(V(brng1)$selfloop)

## [1] 0.2207207

sum(V(ng)$selfloop==1)/length(V(ng)$selfloop)
```

```
## [1] 0.003953928

sum(V(brpg)$selfloop==1)/length(V(brpg)$selfloop)

## [1] 0.1029478

sum(V(brng)$selfloop==1)/length(V(brng)$selfloop)

## [1] 0.06952055

wilcox.test(V(ng)$degree~V(ng)$selfloop)

##
##  Wilcoxon rank sum test with continuity correction
##
## data:  V(ng)$degree by V(ng)$selfloop
## W = 19816.5, p-value = 6.498e-10
## alternative hypothesis: true location shift is not equal to 0

wilcox.test(V(brpg)$degree~V(brpg)$selfloop)

##
##  Wilcoxon rank sum test with continuity correction
##
## data:  V(brpg)$degree by V(brpg)$selfloop
## W = 777436, p-value < 2.2e-16
## alternative hypothesis: true location shift is not equal to 0

wilcox.test(V(brng)$degree~V(brng)$selfloop)

##
##  Wilcoxon rank sum test with continuity correction
##
## data:  V(brng)$degree by V(brng)$selfloop
## W = 401391, p-value < 2.2e-16
## alternative hypothesis: true location shift is not equal to 0

# Plot Normal network
for(i in c("ng1","brpg1","brng1")){
sg=eval(parse(text=i))
cols=rep(rgb(t(col2rgb("orange",alpha=0.5)),maxColorValue=255),length(E(sg)$t.stat))
cols[E(sg)$t.stat<=0]=rgb(t(col2rgb("slateblue",alpha=0.5)),maxColorValue=255)
resetd(rdp)
calld(rdp)
sg <- att.setv(sg, from="selfloop", to="nodeColor", cols=c("grey","deeppink"))
sg <- att.setv(sg, from="degree", to="nodeSize",isrev=F,nquant=10,xlim=c(1,50,0))
sg <- set.edge.attribute(graph=sg,name="color",value=cols)
sg <- att.sete(sg,from="color",to="edgeColor",cols=c("slateblue","orange"))
```

```
sg <- set.edge.attribute(sg,"arrowType",value=1)
sg <- set.vertex.attribute(sg,"nodeFontSize", value=14)
sg <- set.edge.attribute(sg,"arrowDirection",value=rep(1,length(E(sg)$t.stat)))

addGraph(rdp,sg)
relax(rdp)
Sys.sleep(10)

d1=dist(get.adjacency(sg,attr="weight"))
hc <- hclust(d1)
nesthc(rdp,hc, cutlevel=3, nmemb=5,labels=V(sg)$nodeAlias)
relax(rdp)
Sys.sleep(10)
}

## *** Uploading graph to RedeR server ***
## ** ...  nodes!
## ** ...  edges!
## *** Uploading node attributes ...
## ** ...  node 'coords'
## ** ...  node 'size'
## ** ...  node 'color'
## ** ...  node 'font size'
## *** Uploading edge attributes ...
## Warning in .local(obj, g, ...):  NOTE: edge 'direction' must be
provided as integers!
## ** ...  edge 'weight'
## ** ...  edge 'color'
## *** Uploading nest hclust...
## *** Uploading graph to RedeR server ***
## ** ...  nodes!
## ** ...  edges!
## *** Uploading node attributes ...
## ** ...  node 'coords'
## ** ...  node 'size'
## ** ...  node 'color'
## ** ...  node 'font size'
## *** Uploading edge attributes ...
## Warning in .local(obj, g, ...):  NOTE: edge 'direction' must be
provided as integers!
## ** ...  edge 'weight'
## ** ...  edge 'color'
## *** Uploading nest hclust...
```

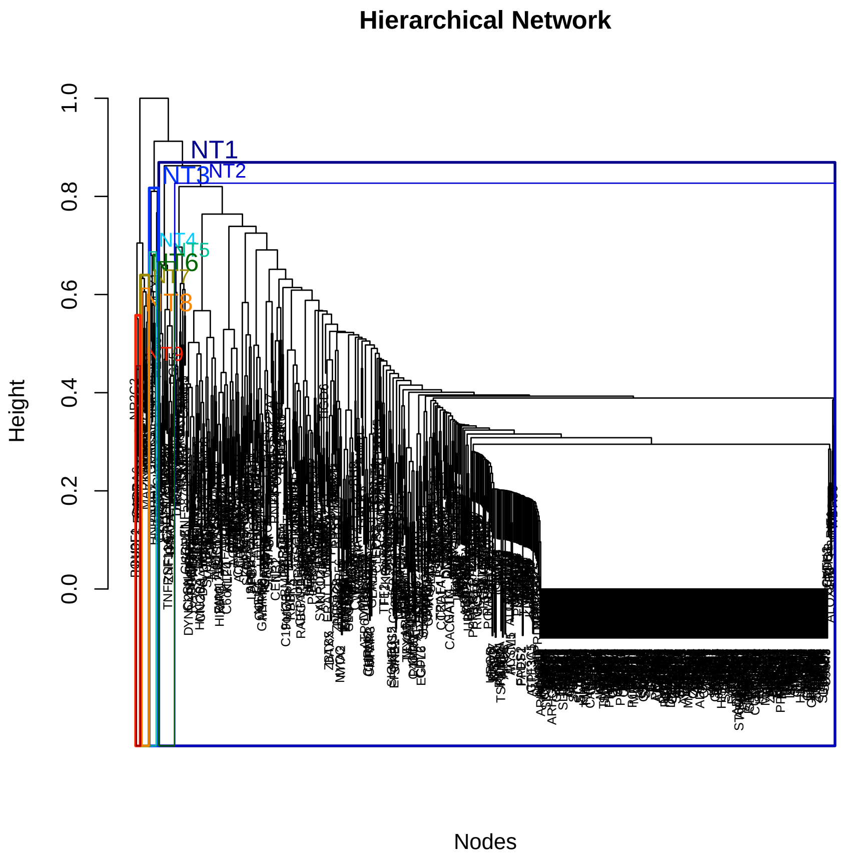


```
## *** Uploading graph to RedeR server ***
## ** ...  nodes!
## ** ...  edges!
## *** Uploading node attributes ...
## ** ...  node 'coords'
## ** ...  node 'size'
## ** ...  node 'color'
## ** ...  node 'font size'
## *** Uploading edge attributes ...
## Warning in .local(obj, g, ...):  NOTE: edge 'direction' must be
provided as integers!
## ** ...  edge 'weight'
## ** ...  edge 'color'
## *** Uploading nest hclust...
```

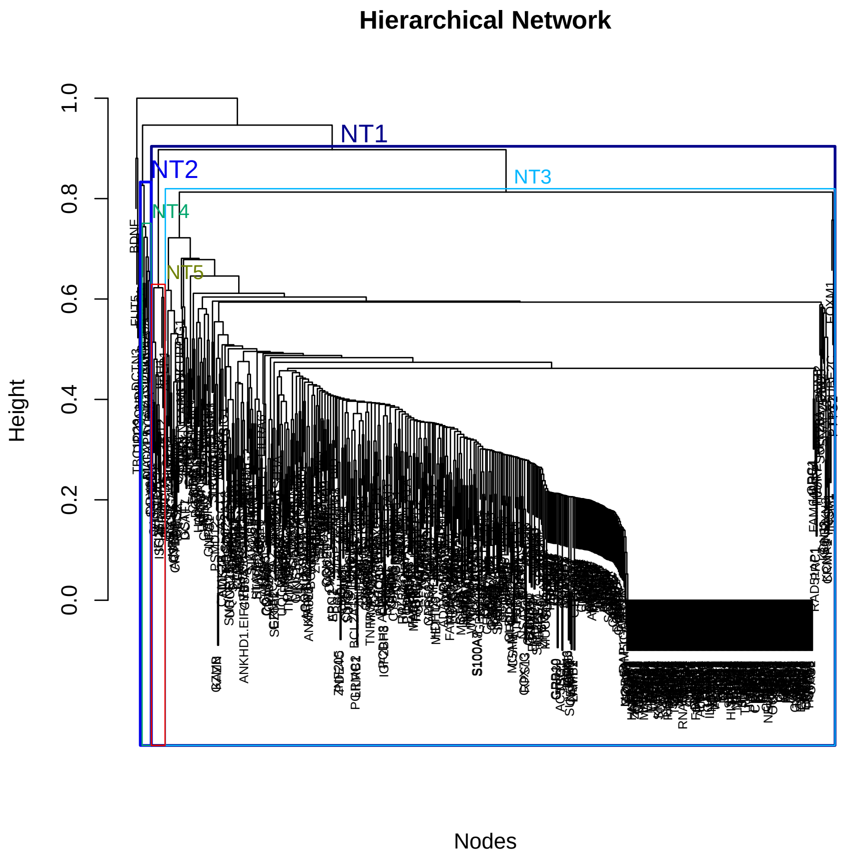

Hierarchical Network
Height
1.0
0.8
0.6
0.4
0.2
0.0
NT1
NT2
NT3
NT4
NT5
BDNF
Nodes

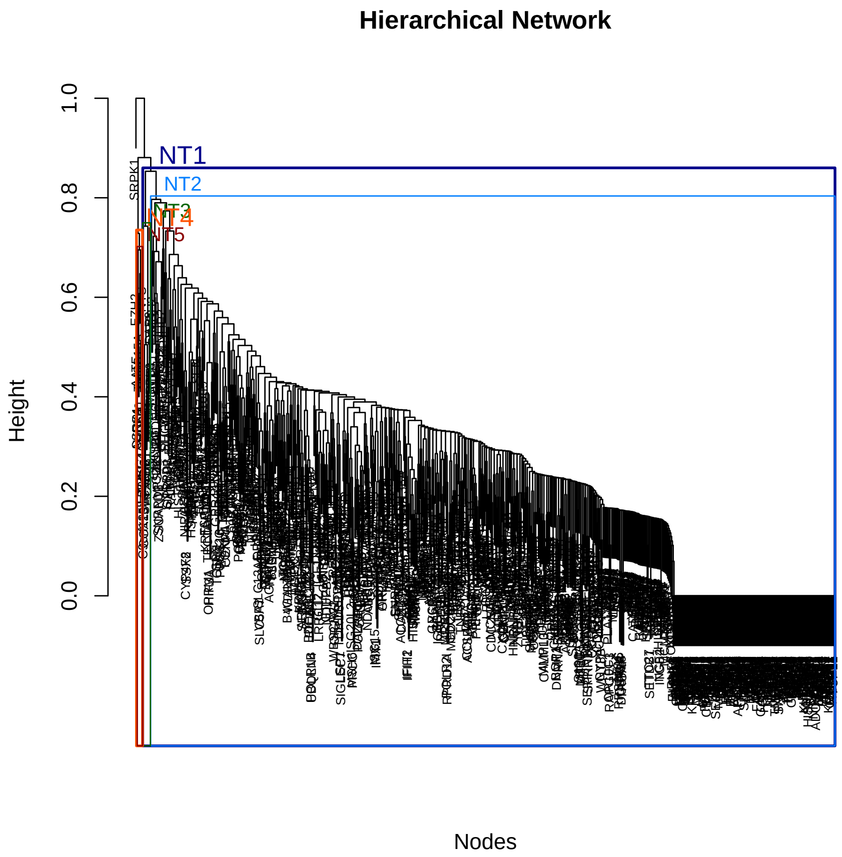

9 SANTA Network Analysis

This section shows how we run the SANTA analysis for enrichment of genesets. As an example, we only use 3 prognostic gene signatures in this Sweave file, for brevity and since this can take hours to run. The p-values we generate here are raw p-values. For the full analysis, we repeated this for all the groups of genesets, merged the results, and adjusted the p values for multiple hypotheses using the p.adjust function with the "fdr" method.

```
# Example SANTA analysis with 3 Prognostic Signatures

if(1){
   f1= 'https://raw.githubusercontent.com/becklab/esnet/master/PrognosticSignatures.txt'
   my_data <- getURL(f1,ssl.verifypeer=FALSE)
   sigs=read.table(textConnection(my_data),row.names=1,sep="\t",header=T)
```

```
  sigs=sigs[,c("Carter.2006..CIN.70.","Rody.2009..interferon.", "Sotiriou.2006..GGI." )]
  genesetNames=colnames(sigs)
  genesets=list()
  for(i in 1:ncol(sigs)){
    genesets[[i]]=rownames(sigs)[sigs[,i]=="x"]
  }
}

#Analysis with Cell Type Specific Signatures
if(0){
  f1= 'https://raw.githubusercontent.com/becklab/epistromanetwork/master/GeneSets/CellTypeS
  my_data <- getURL(f1,ssl.verifypeer=FALSE)
  sigs=read.table(textConnection(my_data),sep="\t",header=T)
  genesetNames=colnames(sigs)
  genesets=list()
for(i in 1:ncol(sigs)){
  genesets[[i]]=unique(as.character(sigs[,i]))
  genesets[[i]]=genesets[[i]][genesets[[i]]!=""]
}
}

# Analysis with MSIGDB genesets
if(0){
  file="c5.bp.v4.0.symbols.gmt"
  #file="c2.cp.kegg.v4.0.symbols.gmt"
  geneset.obj<- GSA.read.gmt(file)
  genesets=geneset.obj[[1]]
  genesetNames=unlist(geneset.obj[[2]])
}

## Perform SANTA on each geneset
norm.ps=rep(NA,length(genesetNames))
brp.ps=rep(NA,length(genesetNames))
brn.ps=rep(NA,length(genesetNames))

ng=graph.data.frame(norm)
brpg=graph.data.frame(BrP)
brng=graph.data.frame(BrN)

ng <- set.edge.attribute(ng,"weights",value=1/abs(E(ng)$t.stat))
brpg <- set.edge.attribute(brpg,"weights",value=1/abs(E(brpg)$t.stat))
brng <- set.edge.attribute(brng,"weights",value=1/abs(E(brng)$t.stat))

nperms=25
```

```
# Normal
for(i in 1:length(genesetNames)){
   vw=1*(is.element(V(ng)$name,unlist(genesets[[i]])))
   if(sum(vw)){
      ng <- set.vertex.attribute(ng,"pheno",value=vw)
      norm.ps[i]=Knet(ng, nperm=nperms, edge.attr="weights", vertex.attr="pheno",verbose=F,c
   }else{
      norm.ps[i]=1
   }
   cat("Iteration ",i," of ",length(genesetNames),"\n")
}

## Iteration  1  of  3
## Iteration  2  of  3
## Iteration  3  of  3

normPs=matrix(norm.ps,ncol=1,dimnames=list(genesetNames,"Norm"))
head(normPs[order(normPs),])

##   Carter.2006..CIN.70. Rody.2009..interferon.
##              0.8163164              0.8220318
##    Sotiriou.2006..GGI.
##              0.9082379

# ER+ IBC
for(i in 1:length(genesetNames)){
   vw=1*(is.element(V(brpg)$name,unlist(genesets[[i]])))
      if(sum(vw)){
         brpg <- set.vertex.attribute(brpg,"pheno",value=vw)
         brp.ps[i]=Knet(brpg, nperm=nperms, edge.attr="weights", vertex.attr="pheno",verbose
      }else{
         brp.ps[i]=1
      }
      cat("Iteration ",i," of ",length(genesetNames),"\n")
   }

## Iteration  1  of  3
## Iteration  2  of  3
## Iteration  3  of  3

   brPs=matrix(brp.ps,ncol=1,dimnames=list(genesetNames,"ERPos"))
   head(brPs[order(brPs),])

##    Sotiriou.2006..GGI.   Carter.2006..CIN.70.
##           5.377932e-16           9.662869e-13
## Rody.2009..interferon.
##           1.403699e-06
```

```
# ER- IBC
  for(i in 1:length(genesetNames)){
    vw=1*(is.element(V(brng)$name,unlist(genesets[[i]])))
    if(sum(vw)){
      brng <- set.vertex.attribute(brng,"pheno",value=vw)
      brn.ps[i]=Knet(brng, nperm=nperms, edge.attr="weights", vertex.attr="pheno",verbose
    }else{
      brn.ps[i]=1
    }
      cat("Iteration ",i," of ",length(genesetNames),"\n")
  }

## Iteration  1  of  3
## Iteration  2  of  3
## Iteration  3  of  3

  brnPs=matrix(brn.ps,ncol=1,dimnames=list(genesetNames,"ERneg"))
  head(brnPs[order(brnPs),])

##   Carter.2006..CIN.70.    Sotiriou.2006..GGI.
##           3.838139e-14           4.683372e-08
## Rody.2009..interferon.
##           7.418948e-07
```

10 Figures 4 and 5, Plotting the SANTA Results

```
  f1= 'https://raw.githubusercontent.com/becklab/esnet/master/SANTAresults.txt'
my_data <- getURL(f1,ssl.verifypeer=FALSE)
s.co.adj=read.table(textConnection(my_data),sep="\t",row.names=1,header=T)
type=s.co.adj[,1]
table(type)

## type
##  BRCA_PROG_SIG CELL_TYPE_SPEC          GO_BP           KEGG
##            125             42            825            186

## Figure 4
plot(-log10(s.co.adj[,"Normal.Breast..Epithelial.Stromal."]),-log10(s.co.adj[,"ER.positive..
abline(a=0,b=1)
```

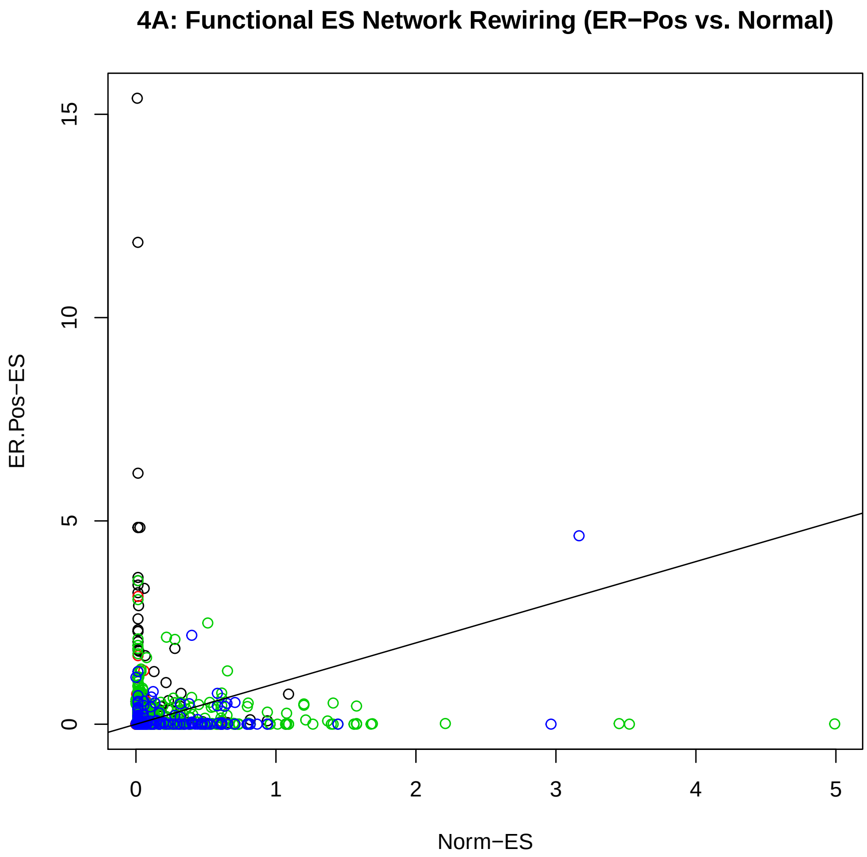


```
plot(-log10(s.co.adj[,"Normal.Breast..Epithelial.Stromal."]),-log10(s.co.adj[,"ER.negative..
abline(a=0,b=1)
```

4B: Functional ES Network Rewiring (ER−Neg vs. Normal)

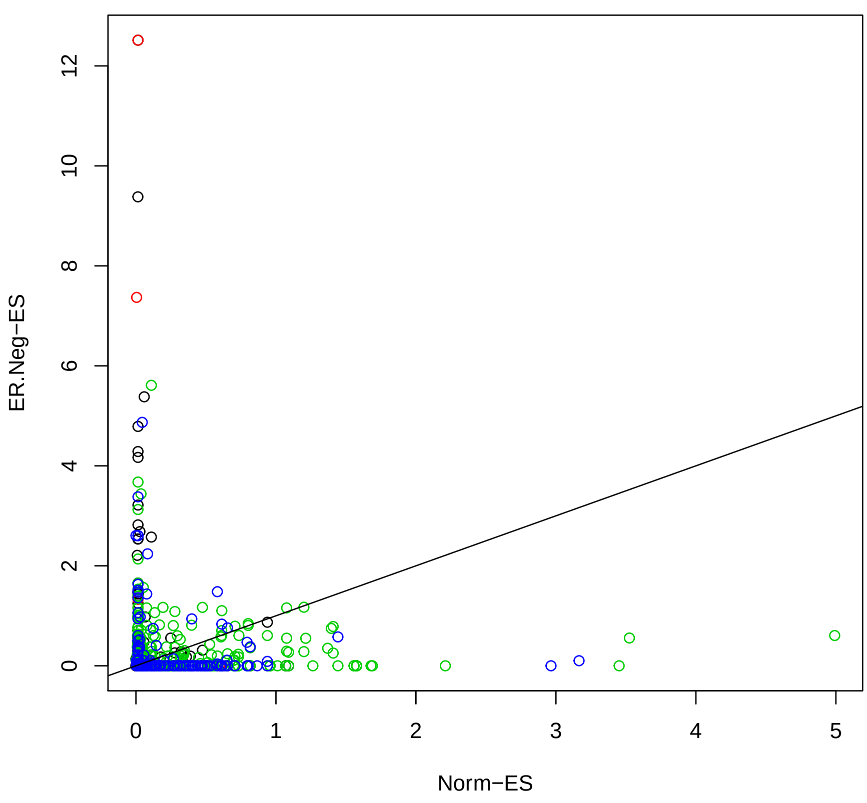


```
plot(-log10(s.co.adj[,"ER.positive..Epithelial.Stromal."]),-log10(s.co.adj[,"ER.negative..Ep
abline(a=0,b=1)
```

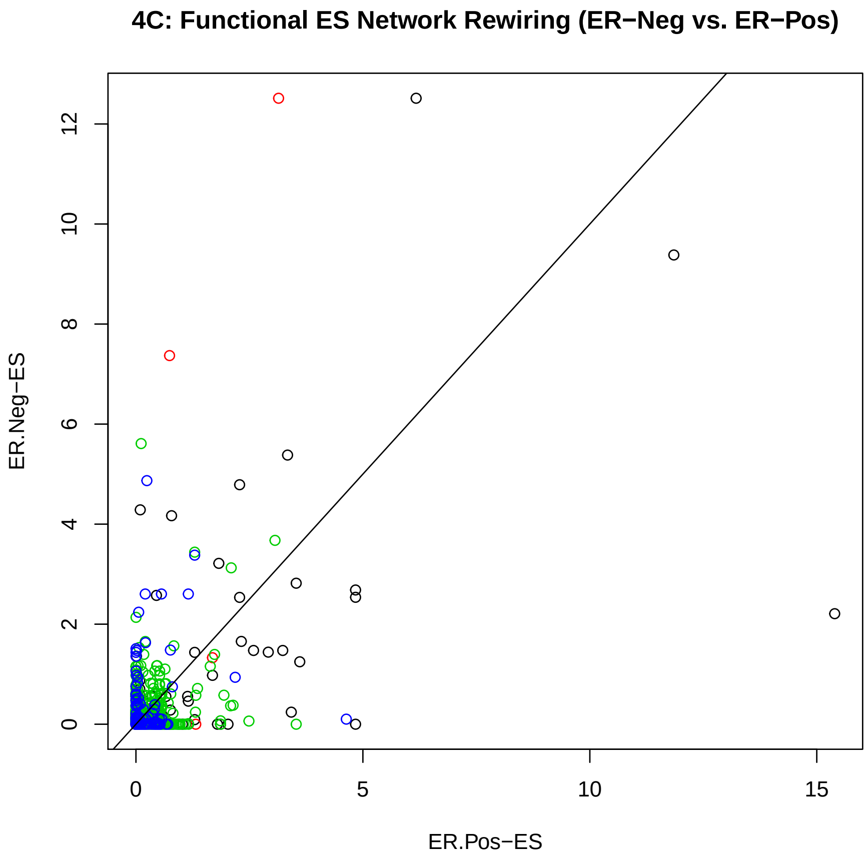


```
## Figure 5
plot(-log10(s.co.adj[,"Normal.Breast..Epithelial.Stromal."]),-log10(s.co.adj[,"Normal.Breast
abline(a=0,b=1)
```

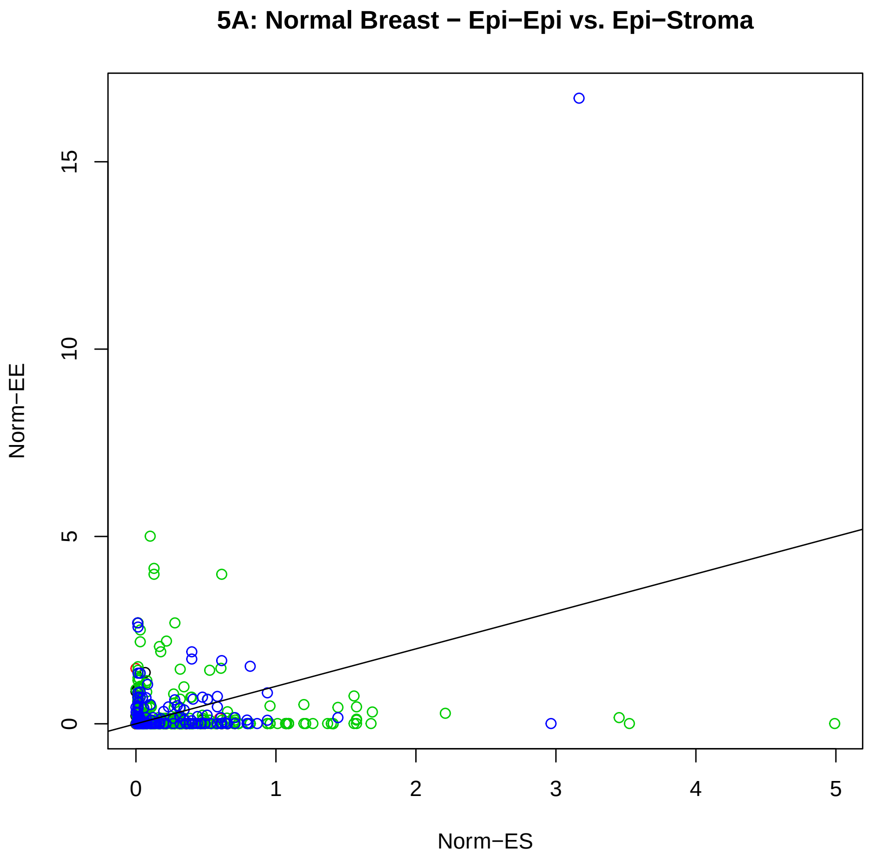


```
plot(-log10(s.co.adj[,"ER.positive..Epithelial.Stromal." ]),-log10(s.co.adj[,"ER.positive..E
abline(a=0,b=1)
```

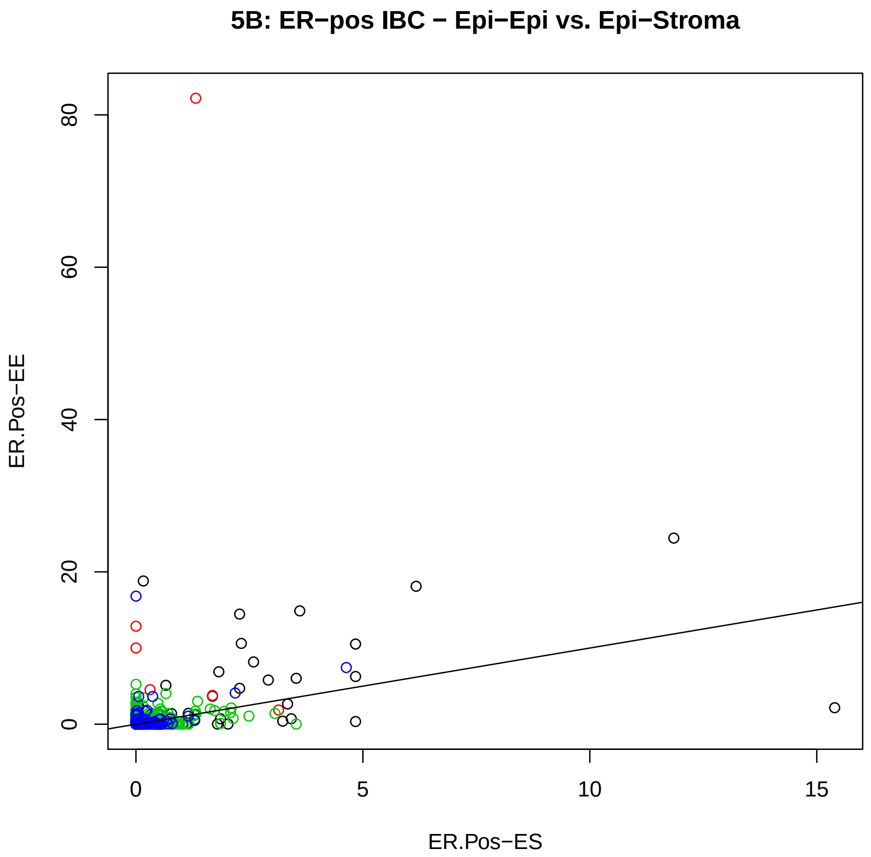


```
plot(-log10(s.co.adj[,"ER.negative..Epithelial.Stromal." ]),-log10(s.co.adj[,"ER.negative..E
abline(a=0,b=1)
```

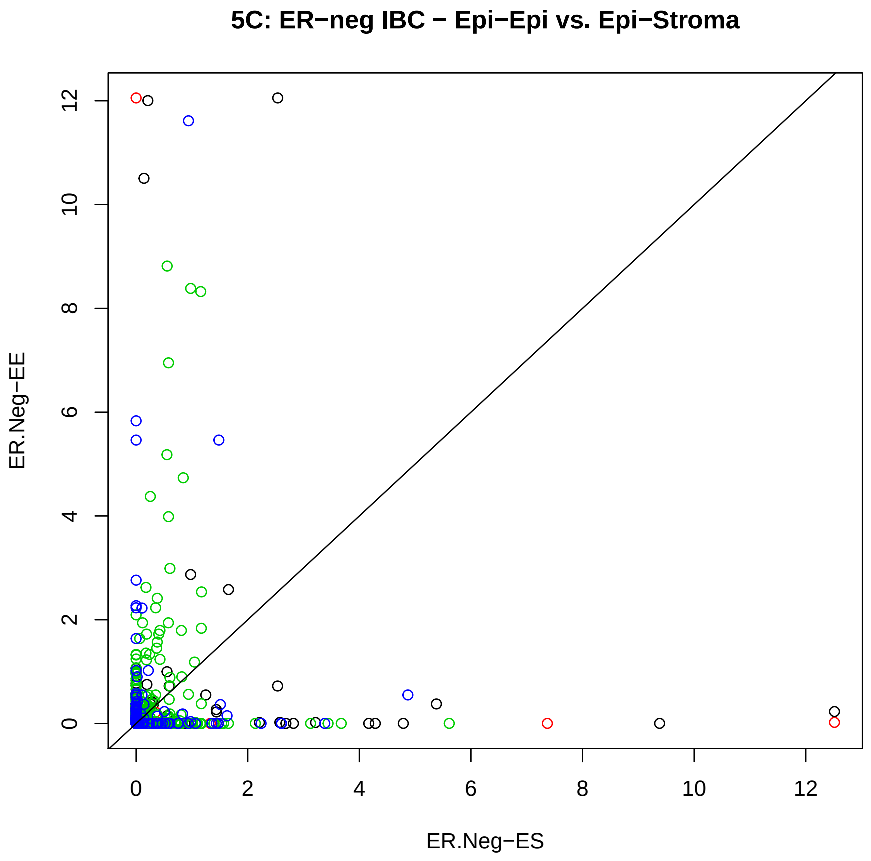


```
# Venn Diagrams
sp=row.names(s.co.adj)[s.co.adj[,"ER.positive..Epithelial.Stromal."]<0.05]
sn=row.names(s.co.adj)[s.co.adj[,"ER.negative..Epithelial.Stromal."]<0.05]
nn=row.names(s.co.adj)[s.co.adj[,"Normal.Breast..Epithelial.Stromal."]<0.05]

sp.ee=row.names(s.co.adj)[s.co.adj[,"ER.positive..Epithelial.Epithelial."]<0.05]
sn.ee=row.names(s.co.adj)[s.co.adj[,"ER.negative..Epithelial.Epithelial." ]<0.05]
nn.ee=row.names(s.co.adj)[s.co.adj[,"Normal.Breast..Epithelial.Epithelial."]<0.05]

# Figure 4D: Venn Diagram of Significant ES Genesets
w=list(Norm.ES=nn,Pos.ES=sp,Neg.ES=sn)
w <- Venn(w)
plot(w)
```

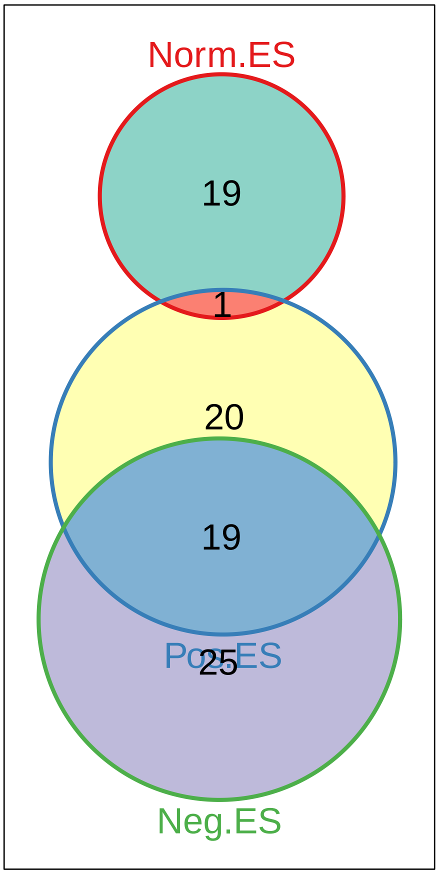


```
# Figure 5D: Venn Digram of Significant ER-Pos ES and ER-Pos EE
w=list(Norm.ES=nn,Norm.EE=nn.ee)
plot(Venn(w))
```

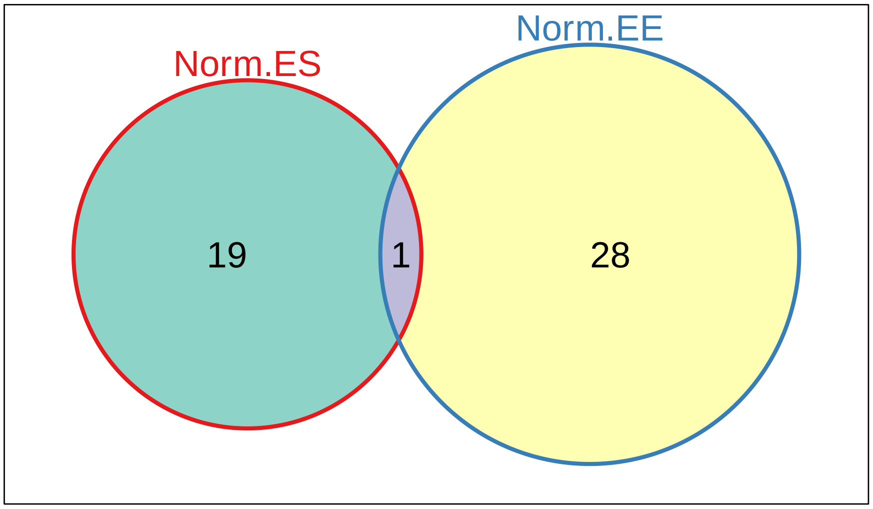


```
# Figure 5E: Venn Digram of Significant ER-Pos ES and ER-Pos EE
w=list(Pos.ES=sp,Pos.EE=sp.ee)
plot(Venn(w))
```

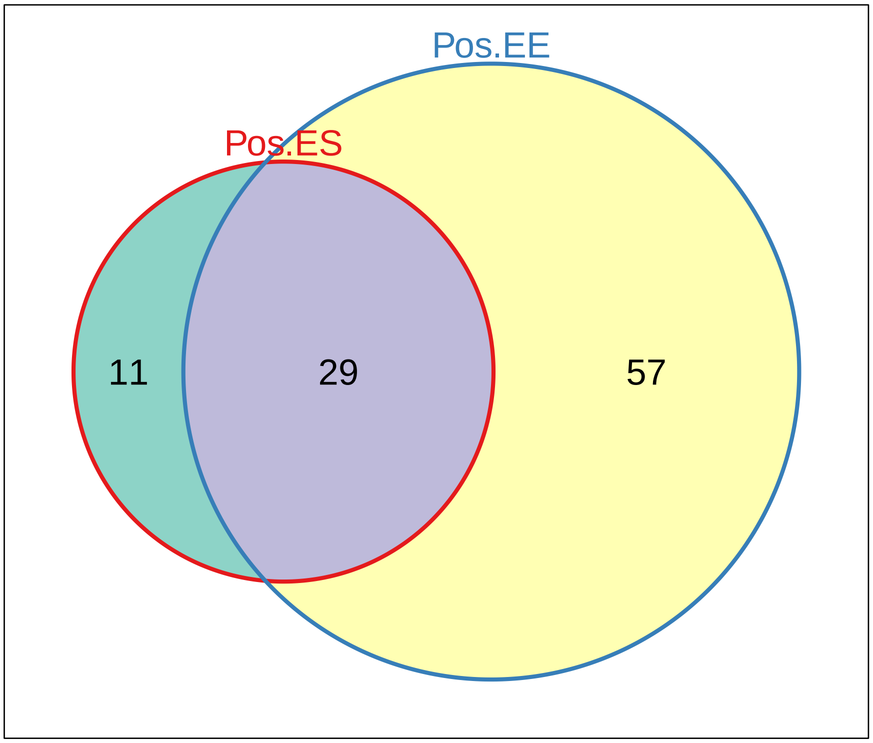


```
# Figure 5F: Venn Diagram of Significant ER-Neg ES and ER-Neg EE
w=list(Neg.ES=sn,Neg.EE=sn.ee)
plot(Venn(w))
```

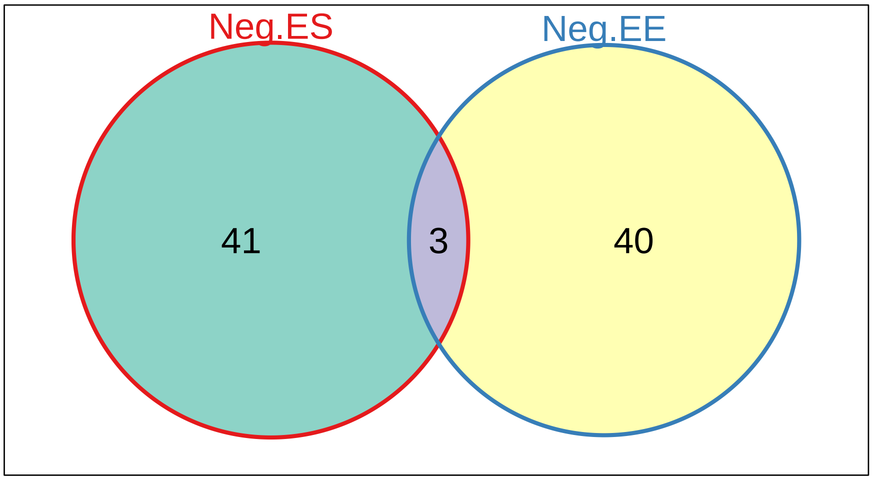

11 Validation Analysis on Park Dataset

```
eDir= 'https://raw.githubusercontent.com/becklab/esnet/master/eset_erp_finak_ex.txt'
aDir = 'https://raw.githubusercontent.com/becklab/esnet/master/eset_erp_finak_anno.txt'
my_data <- getURL(eDir,ssl.verifypeer=FALSE)
e1 <- data.matrix(read.table(textConnection(my_data),sep="\t",header=T,row.names=1))
my_data <- getURL(aDir,ssl.verifypeer=FALSE)
g1 <- read.table(textConnection(my_data),sep="\t",header=T,row.names=1)

type=unlist(lapply(strsplit(colnames(e1),".",fixed=T),function(x)(x[[2]])))
table(type)

## type
## TE TS
## 36 36
```

```
samps=unlist(lapply(strsplit(colnames(e1),"_",fixed=T),function(x)(x[[2]])))

tums.epi=e1[,type=="TE"]
samps.epi=samps[type=="TE"]
tums.str=e1[,type=="TS"]
samps.str=samps[type=="TS"]

dim(tums.epi)

## [1] 18799    36

dim(tums.str)

## [1] 18799    36

rownames(tums.epi)=g1[,"Gene.Symbol"]
rownames(tums.str)=g1[,"Gene.Symbol"]

brCa <- list(Epi=tums.epi,Str=tums.str)
run.eqtl(brCa,"Park_BrCa")

## Processing covariates
## Task finished in  0.01  seconds
## Processing gene expression data (imputation, residualization, etc.)
## Task finished in  0.03  seconds
## Creating output file(s)
## Task finished in  0.02  seconds
## Performing eQTL analysis
## 100.00% done, 591,637 eQTLs
## Task finished in  32.82  seconds
##

## NOW COMPARE RESULTS WITH RESULTS OBTAINED ON ORIGINAL ER-POSITIVE DATASET
BrP=read.table("ER_Positive_ES.txt",header=T,sep="\t")
BrP.park=read.table("Park_BrCa.txt",header=T,sep="\t")

BrP.nn=paste(BrP[,1],BrP[,2],sep=".")
BrP.park.nn=paste(BrP.park[,1],BrP.park[,2],sep=".")

BrP=cbind(BrP.nn,BrP)
BrP.park=cbind(BrP.park.nn,BrP.park)
brc.c=merge(BrP,BrP.park,by.x=1,by.y=1)

t1=table(sign(brc.c[,"t.stat.x"]),sign(brc.c[,"t.stat.y"]))
t1
```

```
##
##       -1   1
##   -1  61  88
##   1   73 920
```

```
sum(t1[1,1],t1[2,2])/sum(t1)
```

```
## [1] 0.8590193
```

```
sum(t1[1,1],t1[2,2])
```

```
## [1] 981
```

```
sum(t1)
```

```
## [1] 1142
```

```
cs1=chisq.test(t1)
cs1$stdres
```

```
##
##              -1         1
##   -1  11.87966 -11.87966
##   1  -11.87966  11.87966
```

```
cs1
```

```
##
##  Pearson's Chi-squared test with Yates' continuity
##  correction
##
## data:  t1
## X-squared = 137.9019, df = 1, p-value < 2.2e-16
```

```
# 1142 edges with raw p value <1e-3 in both
# 981 with concordant direction!
# 86% concordance
cor.test(brc.c[,"t.stat.x"],brc.c[,"t.stat.y"],method="sp")
```

```
##
##  Spearman's rank correlation rho
##
## data:  brc.c[, "t.stat.x"] and brc.c[, "t.stat.y"]
## S = 137901954, p-value < 2.2e-16
## alternative hypothesis: true rho is not equal to 0
## sample estimates:
##       rho
## 0.4444493
```

```
plot(brc.c[,"t.stat.x"],brc.c[,"t.stat.y"],xlab="T-Stat Meta-Dataset, ER-Pos IBC",ylab="T-st
```

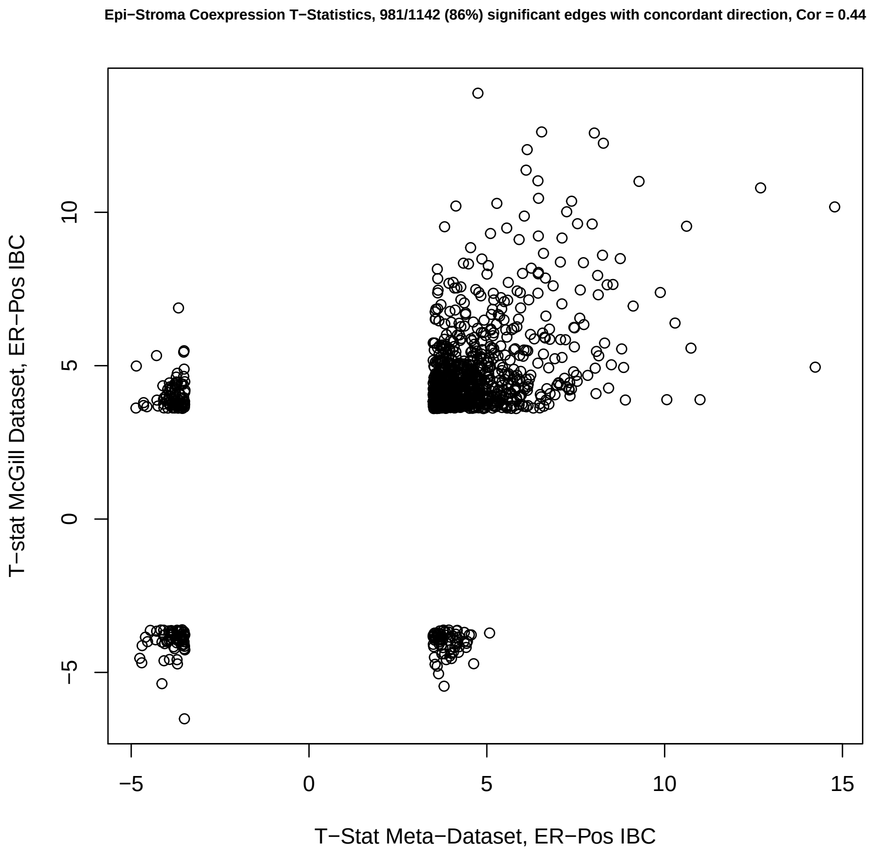


```
park.sl=apply(BrP.park[1:10000,],1,function(x)(1*(x[2]==x[3])))
table(park.sl)

## park.sl
##    0    1
## 9583  417

sum(park.sl==1)/length(park.sl) #4% self-loops

## [1] 0.0417

park.sl.name=as.character(BrP.park[1:10000,2][park.sl==1])
park.sl.name

##   [1] "MUC19"         "GNGT1"         "KCNC2"
##   [4] "UGT2B4"        "SYT4"          "PAGE2B"
##   [7] "MESP1"         "KCNG1"         "GSTT1"
```

```
##  [10] "CDH18"          "TRH"             "GRIA2"
##  [13] "KLK11"          "CLIC6"           "XAGE1"
##  [16] "CUTL2"          "RP13-102H20.1" "TFAP2B"
##  [19] "FLJ34503"       "SCGB3A1"         "SYT13"
##  [22] "PSPH"           "GRB14"           "TAT"
##  [25] "BEX1"           "ORM2"            "GATA5"
##  [28] "LIPF"           "VRK2"            "FBXO2"
##  [31] "SLC6A4"         "CITED4"          "PIK3CD"
##  [34] "ALG8"           "GREB1"           "TCN1"
##  [37] "CBLN2"          "CLNS1A"          "S100A7"
##  [40] "C8orf34"        "CYP4Z1"          "GPR158"
##  [43] "FLJ22671"       "CYP2B6"          "GPR110"
##  [46] "FAM3B"          "KLK12"           "NR0B1"
##  [49] "BCAR4"          "CPA6"            "TFF1"
##  [52] "RPESP"          "S100P"           "BAMBI"
##  [55] "CAMK2N1"        "S100A9"          "LOC284600"
##  [58] "C17orf37"       "RARRES3"         "ORM1"
##  [61] "KLK10"          "BEX2"            "UNG2"
##  [64] "LTF"            "GFRA1"           "GHRH"
##  [67] "TMCO2"          "WFDC2"           "TUBB3"
##  [70] "OBP2B"          "KCTD15"          "CRISP3"
##  [73] "YBX2"           "SERPINA1"        "PAGE2"
##  [76] "C1orf64"        "SERPINA3"        "FCRLM2"
##  [79] "DPEP1"          "CD24"            "FBXL16"
##  [82] "RABEP1"         "CAPS"            "CEACAM6"
##  [85] "PTPRT"          "AQP5"            "C18orf2"
##  [88] "S100A14"        "KRT23"           "CRYBA1"
##  [91] "DLX2"           "SLITRK6"         "GNG4"
##  [94] "NPY1R"          "AREG"            "CCDC74B"
##  [97] "SUSD3"          "NKX3-1"          "TNNT1"
## [100] "SALL2"          "C19orf33"        "TMC5"
## [103] "LOC389458"      "PDZK1"           "FOXJ1"
## [106] "UNQ473"         "ACTR3B"          "ACOX2"
## [109] "TPD52L1"        "LAD1"            "SNCB"
## [112] "MUC1"           "SNCG"            "FLJ31196"
## [115] "FAM14B"         "NTN1"            "COL2A1"
## [118] "HEBP1"          "CPNE4"           "MLC1"
## [121] "HMBOX1"         "CFB"             "TNS4"
## [124] "SLC38A3"        "LAMB3"           "GAD1"
## [127] "PIP"            "NAT1"            "PPP1R1C"
## [130] "DSP"            "TCL1B"           "GLYATL1"
## [133] "PLAC1"          "PLP1"            "CREB3L4"
## [136] "HS6ST3"         "MGC45438"        "DHCR7"
## [139] "NKX2-2"         "MUC15"           "OR2A20P"
## [142] "VGLL1"          "C10orf81"        "VTCN1"
```

```
## [145] "DPYSL4"        "CP"            "TMPRSS4"
## [148] "COPS7A"        "ALDH6A1"       "UBE2C"
## [151] "ANG"           "MS4A8B"        "SLC1A1"
## [154] "RGS22"         "PYCARD"        "NEURL"
## [157] "NOL6"          "HLXB9"         "PHLDA2"
## [160] "EGR4"          "CYP4B1"        "CYP4X1"
## [163] "C12orf46"      "LOC133874"     "MTA1"
## [166] "ACTN2"         "CST6"          "SSFA2"
## [169] "GALNT3"        "KCNC1"         "DCD"
## [172] "PI15"          "H1F0"          "LOC253012"
## [175] "CBS"           "CST9"          "PCSK1"
## [178] "CRISP2"        "MRPL13"        "CAPSL"
## [181] "LOC285878"     "DHRS2"         "DPP3"
## [184] "QDPR"          "AGXT2"         "CHST8"
## [187] "GABRE"         "FAM83D"        "WBP1"
## [190] "KREMEN2"       "A2BP1"         "MTCH2"
## [193] "DPPA2"         "TEX14"         "RBP1"
## [196] "ERBB2"         "CNGA1"         "ALB"
## [199] "OASL"          "PERLD1"        "STARD10"
## [202] "ISG20"         "FBP1"          "FAM60A"
## [205] "SCGB2A1"       "FLJ12993"      "UGT2B17"
## [208] "ZC3H12A"       "FLJ37478"      "C16orf45"
## [211] "SDS"           "LOC56964"      "PRRT2"
## [214] "HIST2H2AA3"    "AZGP1"         "FOSB"
## [217] "HOXA9"         "LCE2A"         "RAMP1"
## [220] "CRYL1"         "CEACAM1"       "SSTR2"
## [223] "SCUBE2"        "CCNT1"         "HSPA2"
## [226] "GDNF"          "EDN2"          "STMN3"
## [229] "MAPT"          "HIST1H2AK"     "CA12"
## [232] "HLA-F"         "ECHDC3"        "WNK4"
## [235] "FAM12B"        "GTF3C1"        "SOX13"
## [238] "PRKAG3"        "SULT1E1"       "CRABP2"
## [241] "SAMD10"        "MRPL21"        "AKR7A3"
## [244] "MSMB"          "SPINK4"        "CKMT1B"
## [247] "TYMS"          "MUC3A"         "C6orf51"
## [250] "C17orf81"      "C4orf25"       "SQLE"
## [253] "MSX2"          "PAGE5"         "ABLIM3"
## [256] "MCHR2"         "PCDH8"         "PTPRN2"
## [259] "SLC39A4"       "HSPH1"         "GSTM3"
## [262] "OSBPL6"        "REEP6"         "STC2"
## [265] "DHDH"          "MGC52282"      "HEXIM2"
## [268] "TMEM132A"      "UBD"           "MGC42157"
## [271] "CPA5"          "OLFM4"         "PHF21B"
## [274] "CDH1"          "ATP6V0A4"      "HIST1H2AH"
## [277] "MOP-1"         "HPGD"          "HIST1H2AD"
```

```
## [280] "COL4A6"        "PIGR"          "ATP1B1"
## [283] "MT1M"          "KIAA0101"      "LOC646652"
## [286] "GDF2"          "HIST1H1C"      "ISG15"
## [289] "SCAMP5"        "BOK"           "PALM"
## [292] "SELENBP1"      "C4orf19"       "PRAME"
## [295] "C16orf75"      "EFCAB4A"       "TFE3"
## [298] "GPX4"          "PSME2"         "BIK"
## [301] "WIT1"          "HIST1H2AE"     "CDKN2D"
## [304] "IRF7"          "LOC340109"     "FUT3"
## [307] "BDH2"          "EGF"           "UHRF1"
## [310] "C17orf71"      "RTBDN"         "TNIP3"
## [313] "UNQ501"        "PLSCR2"        "BIRC5"
## [316] "GLI3"          "HOXA13"        "COL4A5"
## [319] "LY6G6C"        "ZNF202"        "XRCC3"
## [322] "HIST1H2AG"     "CRIP1"         "S100A6"
## [325] "LOC392979"     "DKFZp686I1569" "C6orf61"
## [328] "RP6-213H19.1"  "TMEM16C"       "TRIP6"
## [331] "AUTS2"         "IFITM1"        "MICB"
## [334] "RBBP7"         "TGM4"          "HGD"
## [337] "ANKRD40"       "OR5L2"         "NTNG1"
## [340] "GZMH"          "DNALI1"        "ZP3"
## [343] "MAB21L2"       "TBX1"          "EGR1"
## [346] "IRF5"          "CYP2F1"        "ANXA9"
## [349] "PSCA"          "C9orf58"       "SP5"
## [352] "NR4A1"         "PRC1"          "PRB1"
## [355] "C3orf14"       "FLJ90231"      "DEFB127"
## [358] "LOC388743"     "C4B"           "CDK5R2"
## [361] "OBP2A"         "SLC2A1"        "SLC5A8"
## [364] "SYT12"         "OBSCN"         "AK3L1"
## [367] "DMKN"          "PAK6"          "CHRDL2"
## [370] "RPRML"         "SCAP1"         "LY6K"
## [373] "FSCN3"         "CYP2A6"        "HIST1H2AB"
## [376] "LONRF2"        "SCRN1"         "TAS2R46"
## [379] "TK1"           "LRRC41"        "PSMD3"
## [382] "LOC645249"     "STAT6"         "PGM1"
## [385] "TTMB"          "CDCA8"         "HR44"
## [388] "C1QTNF1"       "FAM79B"        "SAMD13"
## [391] "NAV2"          "FBLIM1"        "HIST1H4G"
## [394] "AMD1"          "SPINK6"        "LOC147710"
## [397] "MULK"          "MANEAL"        "SBEM"
## [400] "HIST2H2AB"     "MYOG"          "PPM1L"
## [403] "CDT1"          "DKFZP547L112"  "KRT15"
## [406] "WDR54"         "PRO0132"       "TAF7"
## [409] "RPL39L"        "TACSTD2"       "CDSN"
## [412] "PPP1R1A"       "CBX3"          "PPIF"
```

```
## [415] "SPAG6"         "FADS2"         "RAB34"

fdr=BrP.park[1:10000,"FDR"]
p1=sum(park.sl[fdr<1e-5])/sum(fdr<1e-5)
p2=sum(park.sl[fdr>1e-5 & fdr<1e-3])/sum(fdr>1e-5 & fdr<1e-3)
p3=sum(park.sl[fdr>1e-3])/sum(fdr>1e-3)

par(las=1)
barplot(c(p1,p2,p3),ylab="Proportion of Self-loops",
  ,names=c("-log(fdr) > 5","5 > -log(FDR) > 3","3 > -log(FDR)"),ylim=c(0,0.2),main="Proporti
```

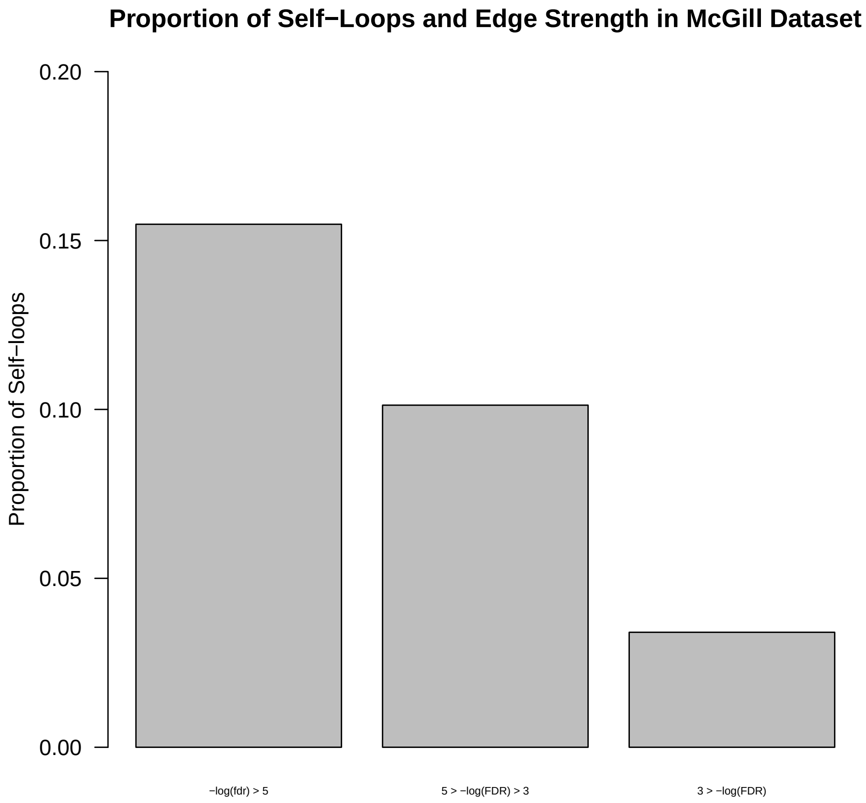


```
## Comparison of self-loops
erp.sl=rownames(Genecomp)[Genecomp[,"erp.self"]==1]
comGenes=unique(rownames(Genecomp),BrP.park[,2],BrP.park[,3])
head(comGenes)
```

```
## [1] "SPINK1"  "PNMA2"   "PLCL1"   "SYNPO2L" "CFTR"
## [6] "SLC4A10"

length(comGenes)

## [1] 10383

park.com.sl=1*(is.element(comGenes,park.sl.name))
table(park.com.sl)

## park.com.sl
##     0     1
## 10134   249

meta.com.sl=1*(is.element(comGenes,erp.sl))
table(meta.com.sl)

## meta.com.sl
##    0    1
## 9695  688

t1=table(park.com.sl,meta.com.sl)
t1

##            meta.com.sl
## park.com.sl    0    1
##           0 9532  602
##           1  163   86

fisher.test(t1) #OR = 8.4, P < 2.2 e-16

##
##  Fisher's Exact Test for Count Data
##
## data:  t1
## p-value < 2.2e-16
## alternative hypothesis: true odds ratio is not equal to 1
## 95 percent confidence interval:
##   6.270455 11.061509
## sample estimates:
## odds ratio
##    8.35109

# Self-loops Identified in Both ER-Positive Datasets
comGenes[meta.com.sl == 1 & park.com.sl ==1]
```

```
##  [1] "TRH"      "GSTM3"    "GSTT1"    "PIP"      "PSMD3"
##  [6] "CEACAM6"  "TCN1"     "BIRC5"    "SSFA2"    "S100A7"
## [11] "GRB14"    "DHRS2"    "PRAME"    "STC2"     "ISG15"
## [16] "AZGP1"    "MSMB"     "COL4A6"   "C4orf19"  "ACOX2"
## [21] "RARRES3"  "GREB1"    "CRIP1"    "AREG"     "GALNT3"
## [26] "PCSK1"    "PCDH8"    "CYP2B6"   "EGF"      "ATP6V0A4"
## [31] "KLK12"    "SELENBP1" "LTF"      "CA12"     "FBXO2"
## [36] "S100A14"  "TFF1"     "CHST8"    "CRABP2"   "ATP1B1"
## [41] "PSCA"     "TFAP2B"   "SLC1A1"   "BEX1"     "CYP4B1"
## [46] "MUC1"     "ORM1"     "BAMBI"    "VTCN1"    "TAT"
## [51] "GAD1"     "GRIA2"    "SERPINA3" "S100P"    "KRT23"
## [56] "SPAG6"    "ANXA9"    "NAT1"     "SCUBE2"   "COL2A1"
## [61] "IFITM1"   "SERPINA1" "PDZK1"    "UBE2C"    "TNNT1"
## [66] "CST6"     "KLK11"    "DPYSL4"   "ACTR3B"   "S100A9"
## [71] "GFRA1"    "VGLL1"    "CFB"      "H1F0"     "RPL39L"
## [76] "TRIP6"    "AKR7A3"   "MTCH2"    "KRT15"    "HSPA2"
## [81] "FOSB"     "ANG"      "S100A6"   "RBP1"     "HIST1H1C"
## [86] "TPD52L1"
```

```
cDir= 'https://raw.githubusercontent.com/becklab/esnet/master/Cancer.HPA.txt'
nDir = 'https://raw.githubusercontent.com/becklab/esnet/master/Normal.HPA.txt'
my_data <- getURL(cDir,ssl.verifypeer=FALSE)
ca <- read.table(textConnection(my_data),sep="\t",header=T,row.names=1)
my_data <- getURL(nDir,ssl.verifypeer=FALSE)
norm <- read.table(textConnection(my_data),sep="\t",header=T,row.names=1)

erp.sl=rownames(Genecomp)[Genecomp[,"erp.self"]==1]
ern.sl=rownames(Genecomp)[Genecomp[,"ern.self"]==1]

genes=as.character(ca[,"Gene.ID"])
genes=substr(genes,1,nchar(genes)-1)

ca.sl=ca[is.element(genes,unique(c(ern.sl,erp.sl))),]
ca.nsl=ca[!is.element(genes,unique(c(erp.sl,ern.sl))),]
dim(ca.sl)

## [1] 283   7

dim(ca.nsl)

## [1] 389   7

epi.rat.no=norm[,"Brown.Spots.in.Epithelium"]/norm[,"Epithelium.pixels"]
str.rat.no=norm[,"Brown.Spots.in.Stroma"]/norm[,"Stroma.pixels"]
```

```r
epi.rat.sl=ca.sl[,"Brown.Spots.in.Epithelium"]/ca.sl[,"Epithelium.pixels"  ]
epi.rat.nsl=ca.nsl[,"Brown.Spots.in.Epithelium"]/ca.nsl[,"Epithelium.pixels"  ]

str.rat.sl=ca.sl[,"Brown.Spots.in.Stroma"]/ca.sl[,"Stroma.pixels"  ]
str.rat.nsl=ca.nsl[,"Brown.Spots.in.Stroma"]/ca.nsl[,"Stroma.pixels"  ]

epi.n.b=Mclust(epi.rat.no[!is.na(epi.rat.no) & !is.na(str.rat.no)],G=2)$class
str.n.b=Mclust(str.rat.no[!is.na(epi.rat.no) & !is.na(str.rat.no)],G=2)$class

epi.nsl.b=Mclust(epi.rat.nsl[!is.na(epi.rat.nsl) & !is.na(str.rat.nsl)],G=2)$class
str.nsl.b=Mclust(str.rat.nsl[!is.na(epi.rat.nsl) & !is.na(str.rat.nsl)],G=2)$class

epi.sl.b=Mclust(epi.rat.sl[!is.na(epi.rat.sl) & !is.na(str.rat.sl)],G=2)$class
str.sl.b=Mclust(str.rat.sl[!is.na(epi.rat.sl) & !is.na(str.rat.sl)],G=2)$class

x=c(sum(epi.n.b==2 & str.n.b == 2),sum(epi.nsl.b==2 & str.nsl.b == 2),sum(epi.sl.b==2 & str.
n=c(length(epi.n.b),length(epi.nsl.b),length(epi.sl.b))
p1=prop.test(x,n) ## 10% vs. 38% vs. 45%
p1

##
##  3-sample test for equality of proportions without
##  continuity correction
##
## data:  x out of n
## X-squared = 123.9199, df = 2, p-value < 2.2e-16
## alternative hypothesis: two.sided
## sample estimates:
##    prop 1    prop 2    prop 3
## 0.1027837 0.3833333 0.4527363

 barplot(p1$estimate,beside=T,names=c("Normal","Cancer-Non-SL","Cancer-SL"),main="Figure 7.
```

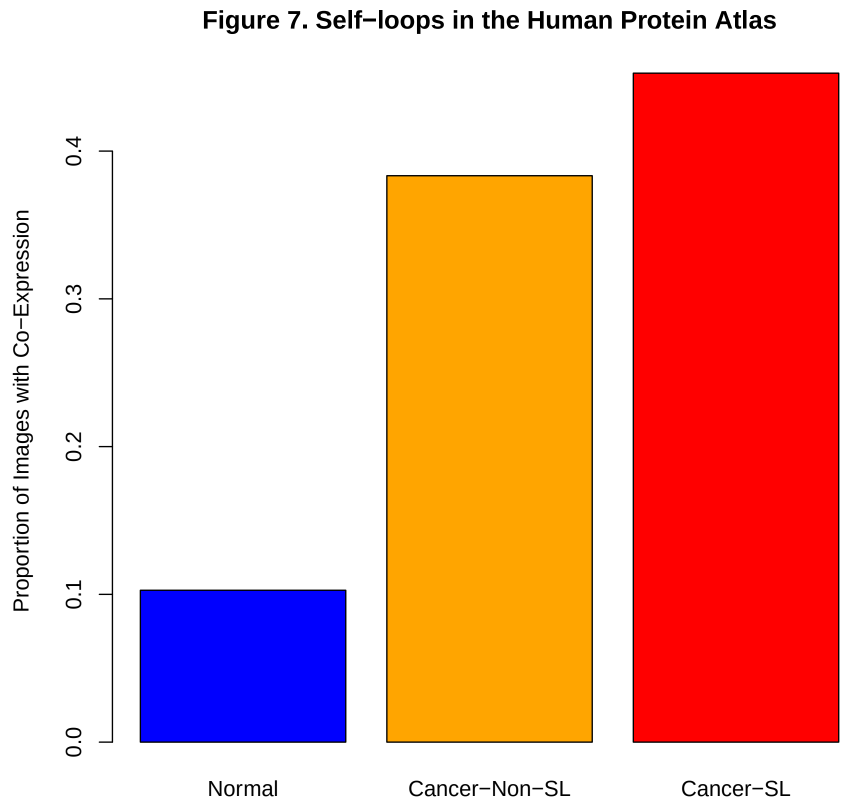


```
x=c(sum(epi.n.b==2 & str.n.b == 2),sum(epi.sl.b==2 & str.sl.b == 2))
n=c(length(epi.n.b),length(epi.sl.b))
p1=prop.test(x,n) ## 10% vs. 45%
p1

##
##  2-sample test for equality of proportions with
##  continuity correction
##
## data:  x out of n
## X-squared = 102.32, df = 1, p-value < 2.2e-16
## alternative hypothesis: two.sided
## 95 percent confidence interval:
##  -0.4276311 -0.2722741
## sample estimates:
##    prop 1    prop 2
```

```
## 0.1027837 0.4527363

x=c(sum(epi.nsl.b==2 & str.nsl.b == 2),sum(epi.sl.b==2 & str.sl.b == 2))
n=c(length(epi.nsl.b),length(epi.sl.b))
p1=prop.test(x,n) ##  38% vs. 45%
p1

##
##  2-sample test for equality of proportions with
##  continuity correction
##
## data:  x out of n
## X-squared = 2.2926, df = 1, p-value = 0.13
## alternative hypothesis: two.sided
## 95 percent confidence interval:
##  -0.15847146  0.01966549
## sample estimates:
##    prop 1    prop 2
## 0.3833333 0.4527363
```
